# Translational Applications of Hydrogels

**DOI:** 10.1021/acs.chemrev.0c01177

**Published:** 2021-05-03

**Authors:** Santiago Correa, Abigail K. Grosskopf, Hector Lopez Hernandez, Doreen Chan, Anthony C. Yu, Lyndsay M. Stapleton, Eric A. Appel

**Affiliations:** † Materials Science & Engineering, Stanford University, Stanford, California 94305, United States; ‡ Chemical Engineering, Stanford University, Stanford, California 94305, United States; § Chemistry, Stanford University, Stanford, California 94305, United States; ∥ Bioengineering, Stanford University, Stanford, California 94305, United States; ⊥ Pediatric Endocrinology, Stanford University School of Medicine, Stanford, California 94305, United States; # ChEM-H Institute, Stanford University, Stanford, California 94305, United States; ∇ Woods Institute for the Environment, Stanford University, Stanford, California 94305, United States

## Abstract

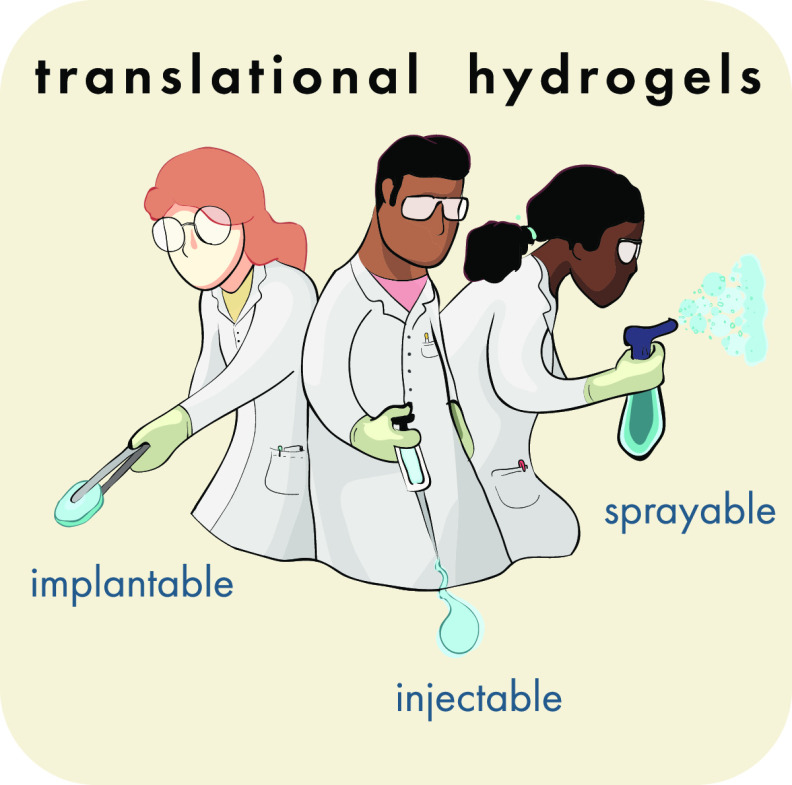

Advances in hydrogel technology have unlocked unique and valuable capabilities that are being applied to a diverse set of translational applications. Hydrogels perform functions relevant to a range of biomedical purposes—they can deliver drugs or cells, regenerate hard and soft tissues, adhere to wet tissues, prevent bleeding, provide contrast during imaging, protect tissues or organs during radiotherapy, and improve the biocompatibility of medical implants. These capabilities make hydrogels useful for many distinct and pressing diseases and medical conditions and even for less conventional areas such as environmental engineering. In this review, we cover the major capabilities of hydrogels, with a focus on the novel benefits of injectable hydrogels, and how they relate to translational applications in medicine and the environment. We pay close attention to how the development of contemporary hydrogels requires extensive interdisciplinary collaboration to accomplish highly specific and complex biological tasks that range from cancer immunotherapy to tissue engineering to vaccination. We complement our discussion of preclinical and clinical development of hydrogels with mechanical design considerations needed for scaling injectable hydrogel technologies for clinical application. We anticipate that readers will gain a more complete picture of the expansive possibilities for hydrogels to make practical and impactful differences across numerous fields and biomedical applications.

## 1 Introduction

**Figure 1:**
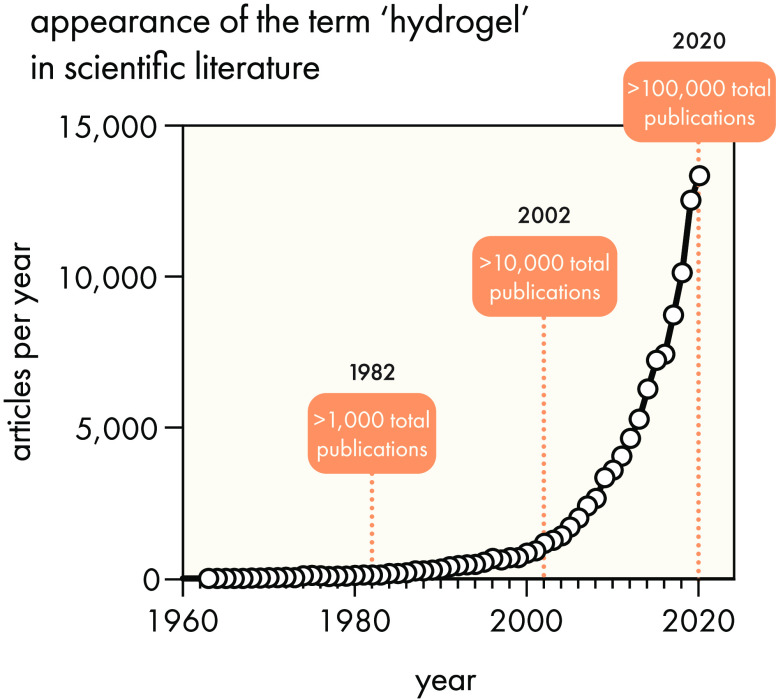
Publications referencing hydrogels have grown exponentially over time since the discovery of synthetic hydrogels in 1960. Data were obtained from a Web of Science search for the term “hydrogel”.

Since their discovery in the 1960s,^1^ synthetic hydrogels have become increasingly useful for engineering biological systems. The enthusiasm over this technology is evident in the explosion of research publications over the past 60 years (Figure 1): from just 1,000 total publications by 1982 to more than 100,000 total publications by 2020! The past three years alone have seen >10,000 publications per year, and with only a few months into 2021 there were already more than 600 new articles indexed in the Web of Science by the time of this publication. Here, we seek to provide a resource for researchers both new and familiar with this technology, delving into many of the fundamentals and open questions of the field and shining a spotlight on both developed and developing applications for these exciting materials.

**Figure 2:**
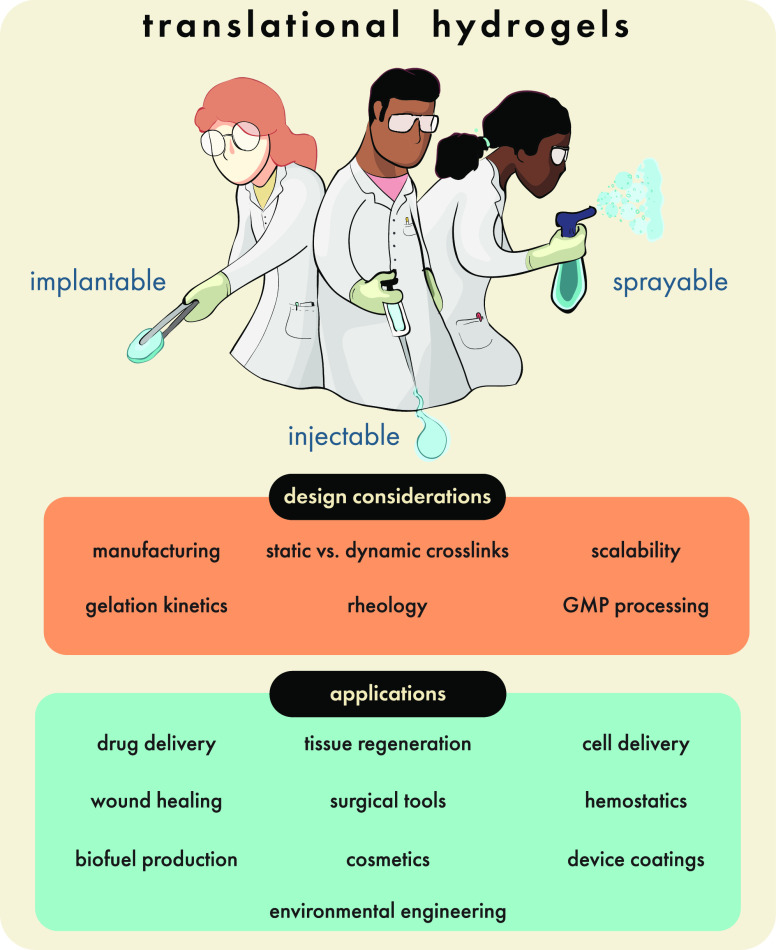
Translational potential of hydrogels has expanded considerably over the past 60 years, leading to implantable, injectable, and sprayable biomaterials with widespread clinical and societal implications. Critical design choices beginning in research laboratories (e.g., synthesis techniques and cross-linking methods) yield hydrogel technologies with distinct rheological properties, which can be applied toward numerous translational purposes. As a platform advances from the initial research and development phase, early design choices form a foundation for the eventual manufacturing challenges to produce a commercial product at scale that meets regulatory standards. Original illustration.

The explosion of hydrogel technologies has made significant contributions in biomedical applications that impact the day-to-day lives of millions of people. For example, hydrogels made one of the most visible (or perhaps we should say invisible?) contributions to modern life in the form of soft contact lenses, creating a new class of optically tunable soft materials and establishing what is today a multibillion dollar industry.^2^ Early studies also revealed the usefulness of engineered hydrogels for delivering diverse drugs,^3−5^ establishing a field for local controlled release of bioactive compounds.^6−10^ In the 1970s, surgeons recognized the utility of hydrogels for reconstructive surgeries,^11,12^ and by the 1990s, hydrogels were becoming a foundational technology for tissue regeneration.^13−16^ The history of hydrogel materials is well reviewed,^17−19^ and the consistent theme has been that hydrogels continue to find new and exciting applications as the underlying technology improves (Figure 2). Emerging applications for hydrogels today include device coatings,^20^ environmental engineering,^21^ soft robotics,^22^ and adoptive cell therapy.^23^

Hydrogels come in many flavors, with diverse capabilities and limitations, but in general these systems can all be described as cross-linked macromolecular networks that retain a significant amount of water. As much as 99% of the weight of a hydrogel can be water, which makes these materials quite friendly to water-enriched biological environments such as the human body. In earlier technologies, harsh mechanisms for macromolecular cross-linking (e.g., toxic agents, radiation, etc.)^24−28^ meant that gelation needed to occur prior to introducing gels to biological systems. Unsurprisingly, this limited the bioengineering applications of hydrogels to superficial environments such as the surface of the eye, an open wound, or an exposed surgical bed.

Subsequent work developed safer cross-linking mechanisms, which began a trend toward triggering gelation *in situ* after injection, providing a minimally invasive way of administering hydrogels to practically any organ or tissue.^29,30^ The most biocompatible iterations of these injectable *in situ* gelling platforms use specific cues from the body to trigger gelation: physiological temperature,^31^ pH,^32^ or ionic strength.^33^ Unlike earlier hydrogels that relied on covalent cross-links, some of these hydrogels have self-healing properties and possess mechanical properties akin to native tissue, capable of countering natural forces and stresses of a body in motion.

More recently, shear-thinning hydrogels were developed that are formed through dynamic and reversible cross-linking.^34^ For example, physical hydrogels use noncovalent interactions (e.g., supramolecular chemistries) between soluble building blocks in order to self-assemble into a dynamic, reversibly cross-linked network.^35,36^ Likewise, reversible covalent cross-linking strategies can yield dynamic networks with similar properties.^37,38^ These “dynamic hydrogels” assembled through reversible cross-links afford the unique property of being injectable even after having formed a gel, due to their shear-thinning and self-healing behaviors. Current research on dynamic hydrogels has revealed novel and useful capabilities that have opened new frontiers for this technology. For example, they can stabilize delicate protein and cellular cargoes to combat pharmaceutical cold-chain limitations,^39^ they can adhere strongly to tissues to form protective barriers and bandages,^40^ and they can be delivered through spray applications to coat complex biological geometries.^41^

While dynamic hydrogels are opening up new translational possibilities, significant progress is also being made to introduce unprecedented levels of functionality into biomaterials. This includes features such as nanoscale patterning of bioactive molecules,^42,43^ programmable drug release,^44,45^ and stimuli-responsive behaviors.^46,47^ As a consequence, much of the research in this space is trending toward increasingly interdisciplinary projects that recruit the expertise of nanotechnologists, chemists, protein engineers, and synthetic biologists to develop sophisticated multifunctional hydrogels. These novel systems include the rise of programmable behavior in hydrogels reminiscent to the behaviors we now associate with digital technology.^48^ For example, significant advancements have been made to transform simple PEG-based hydrogels into responsive systems based on Boolean-logic gating decisions (e.g., YES, AND, OR operations) by incorporating functional peptides and proteins into the hydrogel network.^45,49,50^ Programmable biotechnologies are already leading to smart injectable materials with the potential to degrade or release drugs based on either endogenous or exogenous triggers.^51,52^ As these capabilities continue to mature, multifunctional and programmable hydrogels may provide the technological foundation for platforms that can engage more effectively with the complex, multistage biological events that govern processes such as tissue regeneration and immunity.

As the capabilities of hydrogels have dramatically increased over time, they have unsurprisingly become useful tools for a wide range of fields and disciplines. Here, we primarily focus on the contributions of injectable hydrogel systems to a range of biomedical applications, with an emphasis on dynamic hydrogels. We begin with a discussion of mechanical considerations for injectable hydrogels and specifically how rheological characterization of these systems is critical for developing technologies with translational potential. From there, we provide a general discussion on strategies for delivering diverse therapeutic cargo, such as small molecule drugs, nucleic acids, and proteins. We focus subsequent discussion of drug delivery toward an emerging area of intense research—that of cancer immunotherapy—which presents highly complex and novel challenges for controlled multidrug delivery and engagement of immune cells. In the following section, we summarize key considerations for designing hydrogels meant to engage with and manipulate cells. We then review the extensive work on hydrogels for cellular therapies, spanning their use as both tissue scaffolds and cellular carriers for applications ranging from tissue regeneration to adoptive cell therapy. We then turn our attention toward emergent and promising biomedical frontiers outside of drug delivery and cellular therapies. We review how the capabilities of dynamic, shear-thinning hydrogels are now giving rise to a class of tools that prevent or mitigate complications that can arise from surgery. We also discuss new developments for hydrogels as coatings for implantable devices to improve biocompatibility and introduce novel functionalities. We round out our discussion of biomedical applications with a review of the current clinical landscape for injectable hydrogels, with a particular focus on hydrogels in active clinical trials and current limitations relating to manufacturing and scalability. We also highlight how the lessons learned from biomedical hydrogels are informing advances in new application areas in agriculture, water preservation, and cosmetics. Overall, we anticipate that readers will gain a greater perspective on the range of possibilities available for hydrogel technologies to make substantive contributions to society, as well as the need for vibrant interdisciplinary collaboration to fully translate this potential into real world change.

## 2 Mechanical Consideration for Designing Injectable Hydrogels

**Figure 3:**
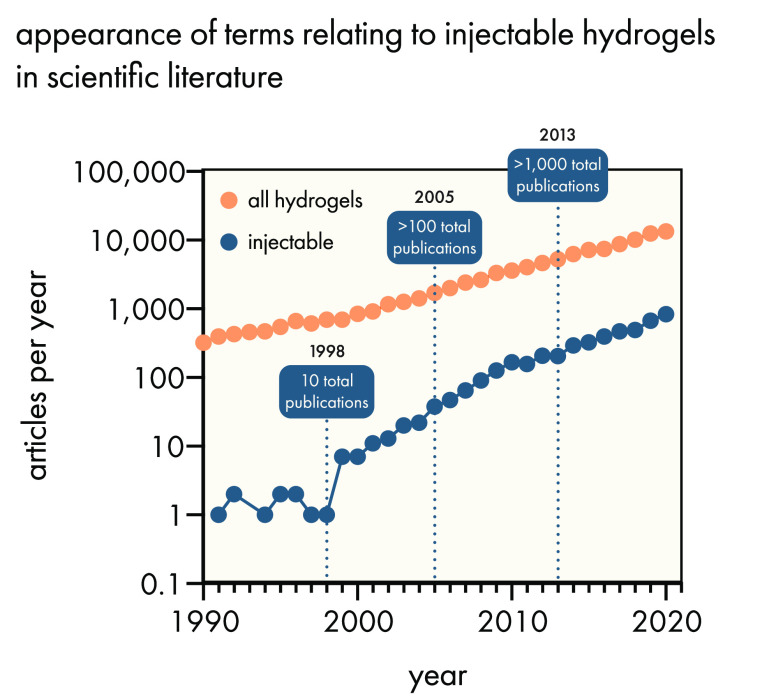
Terminology related to injectable hydrogels begins to appear in the scientific literature by the 1990s, where a variety of strategies were developed to introduce this key functionality. Data were obtained from a Web of Science search for the term “hydrogel” and “thixotropic” OR “shear thinning” OR “injectable”. While the incidence of “hydrogel” generally has grown with a power law exponent of 0.06, the incidence of “injectable” hydrogels has grown with an exponent of 0.11.

Hydrogels are a broad class of materials that exhibit mechanical and chemical properties that are especially useful for a variety of medical interventions. Noninjectable hydrogels represent the bulk of the literature as they were the first to be discovered and developed, and their usefulness for both drug and cell delivery led to broad enthusiasm for developing hydrogels for biomedical applications.^53−56^ However, static covalent cross-links ultimately introduced translational challenges for clinical implementation, since traditional covalent gels require invasive surgical implantation to access nonsuperficial tissues. Additionally, new manufacturing processes, such as 3D printing, require dynamic rheological properties during processing, disqualifying the use of traditional covalent hydrogels.^57^ Interest in further developing the translational potential of hydrogels led to innovative methods to implant them through minimally invasive means, of which the most clinically relevant is injection through a needle or catheter (Figure 3).

Initial success for injectable systems came about with systems that could gel *in situ*, which allowed liquid polymer solutions to be injected into tissues where they subsequently solidify. For example, dual-syringe devices can coinject two solutions that react to form a hydrogel when mixed.^58−60^ Similarly, microencapsulation of gel-inducing molecules could slow down gelation to provide an injection “window” after combining the components of the gel.^61^ Alternatively, stimuli-responsive polymers have been developed that undergo sol–gel transitions based on environmental factors such as temperature, pH, and ionic strength. These systems are engineered to remain liquid under nonphysiological conditions (e.g., room-temperature, acidic pH, salt-free) but solidify when introduced into the body (e.g., 37 °C, neutral pH, millimolar salt concentration).^62−64^ While these systems are injectable, many experience problems with gelation kinetics. For example, they may gel too quickly and solidify within the syringe or gel too slowly and prematurely release cargo *in vivo*, and poor mixing may further cause heterogeneous gelation.^62,65−67^

To overcome these limitations, significant attention has been devoted to dynamic hydrogels, which can seamlessly transition back and forth from solid-like to liquid-like during injection thanks to their shear-thinning and self-healing capabilities. These materials, which are gelled within the syringe before injection, additionally have the ability to stabilize drugs over broad temperature ranges and maintain homogeneously mixed cell solutions.^68−70^ Here, we define dynamic hydrogels as any hydrated polymer network cross-linked via reversible chemistries, which can include both covalent and noncovalent chemistries. Early reports of the unique rheology of dynamic networks emerged in the late 1980s with polysaccharide-based networks covalently cross-linked through boric esters, which identified intriguing self-healing capabilities.^71−73^ However, it was only in the early 2000s that noncovalent chemistries began to be leveraged to make shear-thinning supramolecular hydrogels based on cyclodextrins,^74^ engineered peptides,^75^ and the physical interactions resulting from biopolymer blends.^76^ Although they can be prepared through diverse chemistries, dynamic hydrogels share unique rheological properties that are directly related to their translational potential as injectable systems. In this section, we will review the principle rheological considerations that ought to be taken into account when designing an injectable dynamic hydrogel, as well as a range of techniques to properly characterize these complex systems.

### 2.1 Rheological Considerations for Injectable Dynamic Hydrogels

Injectable hydrogels have enabled minimally invasive strategies to deliver therapeutic drug and cellular cargo without surgical implantation. The applicability of hydrogels in clinical settings is seemingly limitless, from applications that require localization in different regions of the body to the delivery of a wide range of cargo. Importantly, the rheological properties of these hydrogels are constrained by the need for administration by direct injection or catheter delivery. Here, we focus on and discuss the rheological properties of existing injectable hydrogels and emphasize the need for determining property–function relationships to facilitate their design for clinical translation.

**Figure 4:**
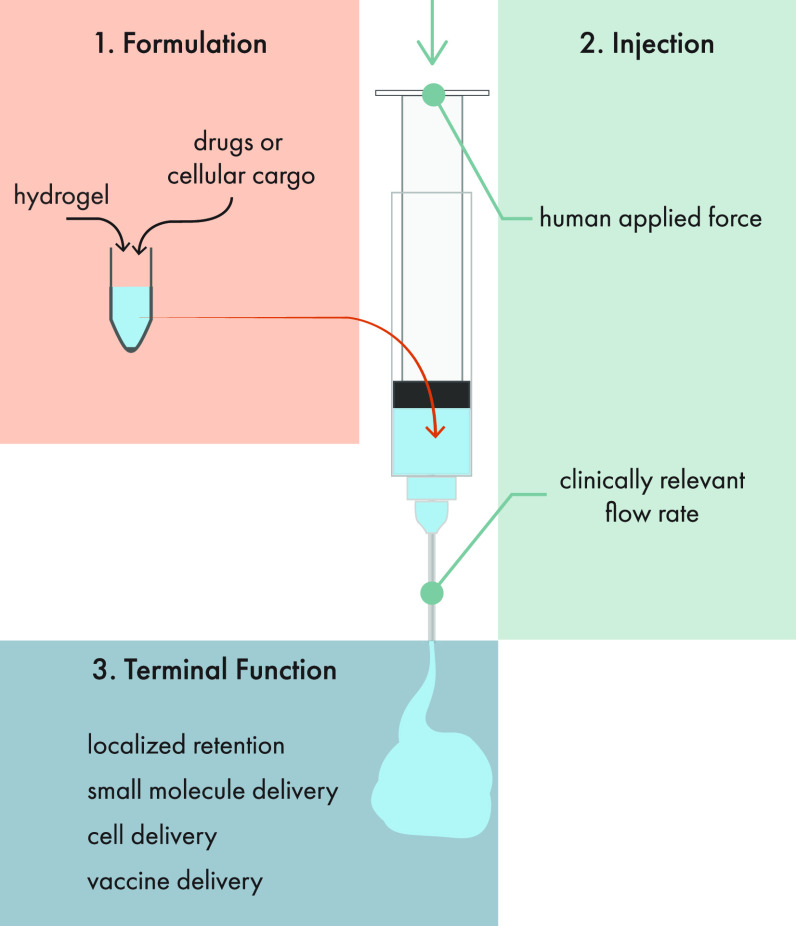
Injectable hydrogels have to accommodate formulation, injection, and terminal function constraints. When designing injectable hydrogels, design considerations have to be made for each stage of the process. The first stage involves formulating a hydrogel that is compatible with the therapeutic cargo of interest. For example, successful formulations generally rely on mild gelation conditions that will not chemically modify or degrade cells or sensitive biotherapeutics such as antibodies. The second stage is injection, where the hydrogel formulation must be injectable through geometries relevant for its final clinical application. So while syringe administration may be appropriate and feasible in a preclinical murine model, it may be that for clinical translation the hydrogel must be able to be injected through a catheter to reach the target tissues. In these cases, the hydrogel formulation must exhibit appropriate rheological behavior to be administered using the end-goal geometries, under forces which are practical and possible in hospital settings. Finally, hydrogels must exhibit mechanical properties suitable to the biomedical goal after injection into the body. For many local delivery applications, this often means the hydrogel must form a solid, biocompatible depot that degrades on a time scale relevant for the specific application, which for drug delivery can vary widely from days to months depending on the goal of the treatment. Original illustration.

Injectable therapeutic hydrogels must be compatible with a three-stage administration process (Figure 4). First, their formulation must be compatible with the incorporation of drug, cellular, or other therapeutic cargo (e.g., the hydrogel must not react with or otherwise compromise the bioactivity of cargo). Second, they must be injectable. Third, they should provide the desired terminal function within the body, which ranges broadly from cell expansion to controlled release of molecular cargo of diverse types. Typically, the terminal function within the body is the key target in the design process, yet the performance of the hydrogel during formulation and administration must not be neglected. From a translational perspective, the injectability of a particular formulation may change as the relevant dimensions and geometries of the injection process changes when moving from the lab to the clinic. Going forward, it is helpful to provide an explicit definition of “injectability”. Here, we define injectability as the capability of a formulation to flow at a clinically relevant flow rate through an administration needle, catheter, or autoinjector using clinically relevant applied pressures. According to this definition, injectability is necessarily dependent on the intended application and will vary depending on the needle gauge and length (i.e., subcutaneous vs catheter injections) and other processing constraints (e.g., administration volumes, syringe geometries, and desired flow rates).

The application-specific requirements imposed by each stage of the administration process can impose paradoxical constraints on the rheological properties of injectable materials, simultaneously requiring flowability for injection and solid-like retention at the injection site (e.g., sustained localized delivery). There have been several hydrogel compositions with varying chemistries and cross-linking modalities developed that address this paradoxical constraint and are capable of both injectability and solid-like retention after injection.

For a material to flow, it must demonstrate liquid-like behavior, whereby the constituent molecules are able to move past each other, under relevant processing conditions. Most covalent materials cannot flow because their covalent bonds prevent relative movement of their constituent molecules. Consequently, “static” covalent hydrogels require the injection of prepolymer systems that gel upon injection or stimuli-responsive polymers that cross-link in response to temperature, UV, pH, or other external stimuli. More recently, there has been an increased interest in the use of dynamically cross-linked hydrogels as injectable materials.^77−80^ The specific cross-linking strategies vary and include both dynamic covalent and noncovalent supramolecular cross-linking, but generally these approaches imbue hydrogels with dynamic, yielding, and self-healing rheological responses. The various cross-linking strategies and description of the hydrogels for the delivery of therapeutics have been outlined in several reviews.^36,81−83^ We highlight that although dynamic hydrogel compositions vary, they demonstrate similar rheological functions. In general, the physical cross-links create a hydrogel network with solid-like material properties under static conditions. Yet, when deformed, the dynamic cross-links can be disrupted, dissipating stress and resulting in liquid-like behavior. Since the cross-links are reversible, they can reassociate after deformation to restore the network structure and its solid-like behavior.

The rapid development of dynamic hydrogels for injectable material platforms has enabled new therapeutic strategies without the need for *in situ* chemical reaction strategies. However, our understanding of structure–property-function relationships (which relate a hydrogel’s rheological properties to their functional performance) for dynamically cross-linked hydrogels is still rather poorly developed.^84−86^ Dynamically cross-linked hydrogels are complex fluids, where their reversible cross-links result in bulk material behaviors that include yielding, shear-thinning, thixotropy, and viscoelasticity. To date, designing injectable hydrogels from dynamically cross-linked networks with the desired combination of properties for new applications remains challenging. Indeed, researchers in the rheological community have focused on creating engineering design strategies for dynamically cross-linked hydrogels.^87−91^ For injectable therapeutic applications, there is a desire to design hydrogel materials with tunable viscoelasticity to deliver stem cells and control their differentiation,^92−98^ a need for strategies to control the release of small molecular cargo,^36,77,81,83,99−101^ and a push toward materials that provide stabilization of pharmaceuticals.^102^

With structure–property–function relationships in place, it becomes easier to answer important design questions before heading to the bench. Questions such as how does one design a hydrogel’s terminal function (i.e., local depot formation for sustained release of molecular cargo) without compromising performance in formulation or during administration by injection? How can one identify if an existing hydrogel formulation would meet the demands of a new application, eliminating the need for starting anew with laborious and costly trial-and-error efforts? Unveiling property–function relationships facilitates the design process of injectable hydrogels. Knowledge of these relationships allows for rapidly identifying and satisfying the functional constraints across a broad variety of administration conditions while optimizing the performance of the injectable hydrogel *in vivo*. The following sections provide a concise review of key property-function relationships of dynamically cross-linked hydrogels for injectable therapeutic applications. We briefly discuss structure–property relationships in the context of the rheological properties that are introduced but leave a detailed discussion to other excellent reviews.^90,98,103,104^ Since cross-linking strategies and network structure result in similar rheological behaviors (i.e., shear thinning, yield stress), the property–function relationships shown here are useful across many hydrogel compositions. Next, we discuss rheological characterization strategies for complex fluids, such as physically cross-linked hydrogels, and provide information about best practices during the characterization process. We intend these sections to help scientists and engineers design future biomaterials and also highlight key areas where more investigations are needed.

### 2.2 Pre- and Postinjection Constraints of Injectable Hydrogels

The applications of injectable hydrogels dictate the requisite properties for the hydrogel during formulation and after injection. The details of the requirements for these applications are left to the other sections of this review. From a rheological perspective, the rheological modifications required by each application must be considered alongside the constraints of injectability. A common requirement is the localization of a hydrogel after injection, which depends strongly on the rate at which the hydrogel self-heals after injection. During injection, the high shear destroys the structure of the dynamic hydrogel. After injection, most dynamic hydrogels do not return to their initial viscosity immediately but rather demonstrate a recovery of viscosity over time.^92,96,104−110^ The transient recovery of viscosity after the cessation of flow (i.e., once in the implantation site after injection) is called thixotropy. Thixotropic behavior in dynamic hydrogels depends heavily on the cross-linking motif, whereby some motifs result in hydrogels that require a significant amount of time to recover (strongly thixotropic), while some show only mild thixotropy and recover their properties rapidly (weakly thixotropic). For injectable drug delivery applications, the thixotropy of a hydrogel provides valuable insight for the time scales over which a hydrogel will be susceptible to burst release or flowing away from the site of injection before establishing a depot.

### 2.3 Relevant Rheological Properties for Injectability

The viscosity of a hydrogel is related to its injectability, elucidating the constraints that injectability places on the viscosity of injectable biomaterials. For clinical applications, injectable hydrogels must be delivered through a needle or catheter to the site of injection. The injectability of a fluid depends on how much pressure is required to drive this process of injection over relevant time frames. This pressure is a function of the fluid viscosity, injection geometry, and desired flow rate. Here, we review how injectability constrains the allowable rheological properties of injectable hydrogels. To elucidate these constraints on rheological properties, it is important to understand the physical process of injection. Injection, in its simplest form, is the flow of a fluid through a circular tube of constant diameter and length. Often, there is a maximum pressure that can be applied and a minimum flow rate that is desired. A syringe injection, for example, would be limited to the amount of force the average healthcare personnel could comfortably apply to a syringe plunger.^111^ An autoinjector on the other hand would be limited by the maximum pressure the mechanism could generate. Intuitively, there is a limit to the viscosity (i.e., resistance to flow) of the materials which can be injected under a prescribed set of injection conditions and geometries. Therefore, it is critical to understand how viscosity—and its dependence on shear rate—affects injectability, enabling researchers to use simple rheological measurements to design their materials for injectability.

**Figure 5:**
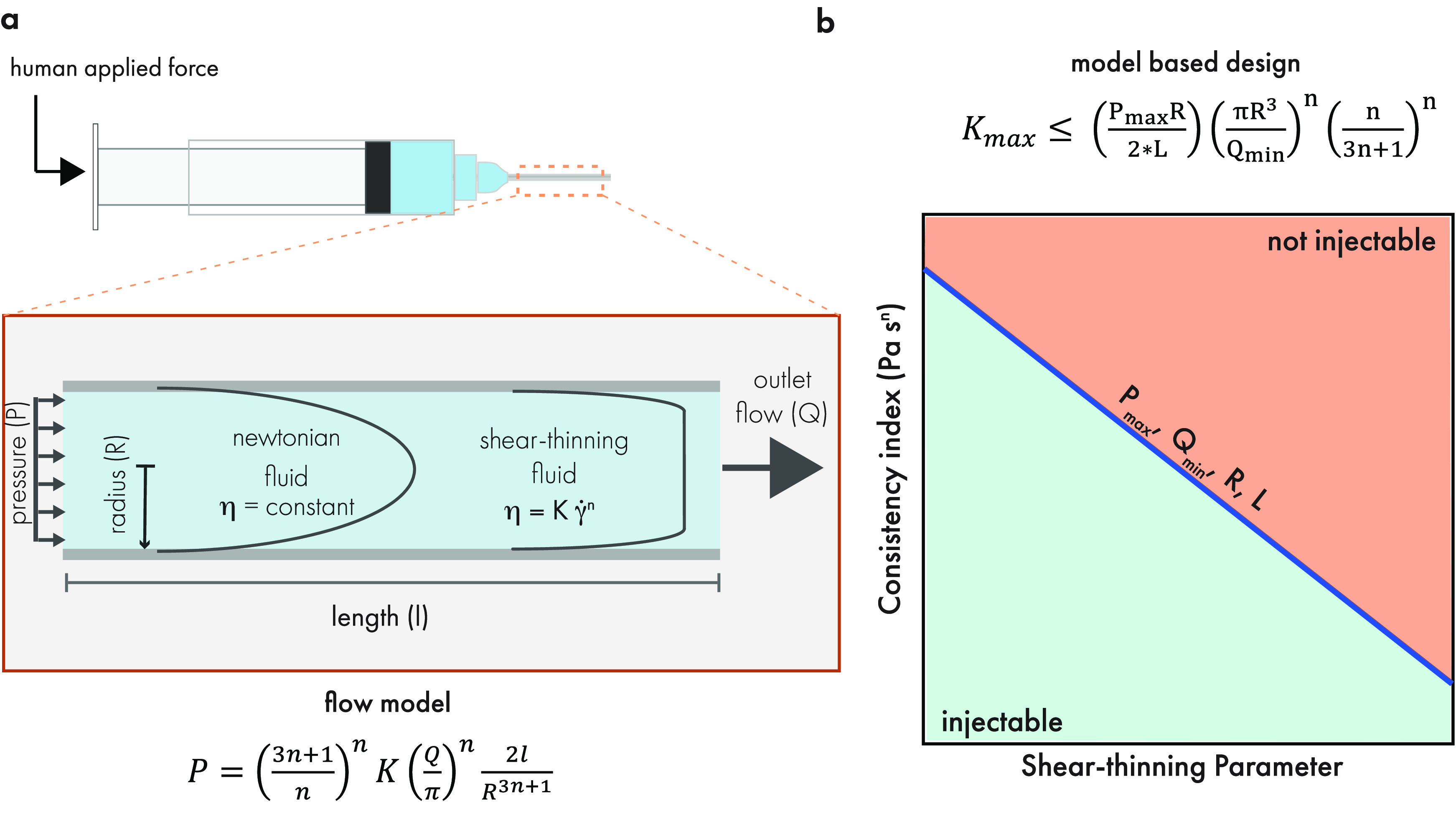
(a) Flow models relate the injection pressure (*P*), flow rate (*Q*), viscosity parameters (*K*, *n*), and geometry (*R*, *l*), allowing for calculation of injection pressures under a variety of clinical scenarios. (b) Flow model used inversely to identify the material parameters that would readily satisfy the desired process and geometrical constraints. The combination of parameters (*K* and *n*) that would satisfy the conditions are shown in an Ashby style plot, where combinations above the line would result in inaccessible pressures or flow rates that are too slow. Original illustration.

Steady state flow models are used to model the relationship between a hydrogel’s viscosity and the pressure required to inject it (Figure 5a).^112−114^ In the case of polymer solutions and physically associated hydrogel materials, the viscosity often obeys a power law (eq 1) that is described by the consistency index, *K*, and shear-thinning parameter, *n*.^115,116^ A shear-thinning parameter of *n* = 1 describes a Newtonian fluid with constant viscosity as the shear rate is increased. A value of *n* < 1 represents a shear-thinning fluid with a viscosity that decreases as the shear rate is increased. Assuming power law shear-thinning behavior, the constitutive relationship shown in eq 2 can be used to describe the relationship between the shear stress and shear rate on the fluid. The governing equation for steady state flow through a pipe (eq 3) is derived using this constitutive relationship to model the injection pressure (*P*) as a function of flow rate (*Q*), radius (*R*), length (*l*), and viscosity.^117−119^ This model has been used by Paxton et al. to predict the bioprinting window for a variety of 3D printing materials.^113^ Almendinger et al. validated the model for shear-thinning antibody solutions and used it to predict the extrusion pressure in a variety of injection scenarios.^112,120−122^ Our group validated the model for physically cross-linked hydrogels, demonstrating its applicability for two physical hydrogels with distinct cross-linking mechanisms (polymer–nanoparticle interactions and ionic cross-linking).^123^
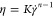
1
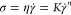
2
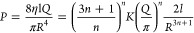
3

In addition to validating the model, our work also demonstrated the utility of using the model inversely for materials design to elucidate the consistency indexes and shear-thinning parameters that correspond to hydrogel injectability across applications with varying geometries or flow rate constraints. Ashby style plots of the consistency index vs shear-thinning parameter were used in combination with the model developed in eq 3 to reveal a parameter space for readily injectable hydrogels under typical process constraints for syringe injection (Figure 5b).^85,87−89^

**Figure 6:**
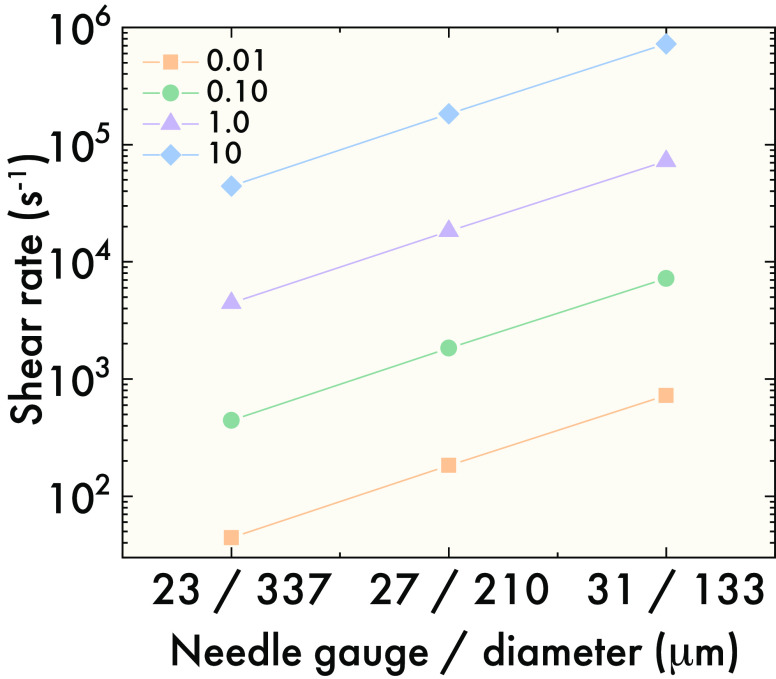
Shear rates vs flow rates for 23-, 27-, and 31-gauge needles at flow rates of 0.01, 0.10, 1.0, and 10 mL/min, showing that even moderate flow rates result in elevated shear rates during injection. Data are calculated using eq 4—which describes the shear rate of a Newtonian fluid—and common needle geometries and flow rates found in the clinic. Shear rates are conservative and would increase if the fluid is shear-thinning.

It is therefore critical that in the development of injectable biomaterials, researchers employ flow models accompanied by rheological characterization to avoid developing material platforms with properties that could never scale to the clinic. This is perhaps most notable for deep-tissue delivery of dynamic hydrogels, which can impose significantly different constraints in a preclinical model versus clinical practice. For example, the primary model for oncological research is mice, where delivery to any organ is possible through a short 0.5–1.0 in. syringe. In contrast, the equivalent in a human patient could require injection through a long catheter and necessitate injection forces that are impractical. To avoid these types of pitfalls on the road to translation, it is imperative that the flow properties of dynamic hydrogels be measured within application relevant shear rate/shear stress regimes to determine the appropriate constitutive relationship for each hydrogel. It is important to note that injections through small diameter needles can result in shear rates which are dramatically higher than the typical shear rates range used for characterization on a rheometer (Figure 6). For example, oncological treatments have improved patient comfort and are delivered at flow rates between 1 and 2.3 mL/min.^124,125^
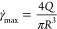
4

Using eq 4—which describes the maximum shear rate for a Newtonian fluid in a pipe—a flow rate of 2.3 mL/min in a 27-gauge needle (standard for subcutaneous injections) results in shear rates up to 42 × 10^3^ s^–1^. Researchers should be cautious of extrapolating constitutive relationships beyond the range of characterization, as this can lead to significant errors and often poor approximations for fluids as complex as dynamically cross-linked hydrogels.^123^

There are limitations to the model presented in eq 3, which simplifies the flow of these hydrogels by assuming a simple power law shear-thinning response (eq 2), steady state conditions, no slip, a negligible yield stress under the flow conditions, and negligible effects of fluid extensibility.^123^ While these assumptions help make the problem simpler to analyze, there may indeed be cases where these simplifications fail to capture the relevant phenomena necessary to describe the flow of more complex fluids. Good practice is to validate the flow model within the target flow regimes and with the appropriate rheological data measured within the correct shear-rates. In the rare cases where the simple model in eq 3 fails to adequately describe the flow behavior, there is extensive literature on the flow of non-Newtonian fluids that should be explored.^114,117−119,126−128^ Alternative models have been developed to account for slip, significant yield stresses, and nonconstant geometries, though these models should be validated with the target materials and desired flow regimes prior to broad utilization by the community.

### 2.4 Rheological Characterization of Injectable Hydrogels

As we have shown, the rheological properties of injectable hydrogels dictate the function and ultimately a significant fraction of the performance as injectable therapeutic strategies. Here, we provide a brief review of characterization methods for measuring the rheological behavior of injectable hydrogels. For an in-depth discussion of these methods, we point the reader to reports by Ewoldt,^129^ Larson,^115^ and Macosko.^116^ Dynamic hydrogels demonstrate rheological behavior that may comprise a combination of yielding, shear-thinning, thixotropic, viscoelastic, and extensible behaviors.^130−135^ Consequently, their characterization is nontrivial and requires a combination of rheological tests to characterize comprehensively. Here, we present the state-of-the-art rheological methods for measuring the viscoelastic and flow behaviors of injectable hydrogels.

#### 2.4.1 Viscoelasticity

**Figure 7:**
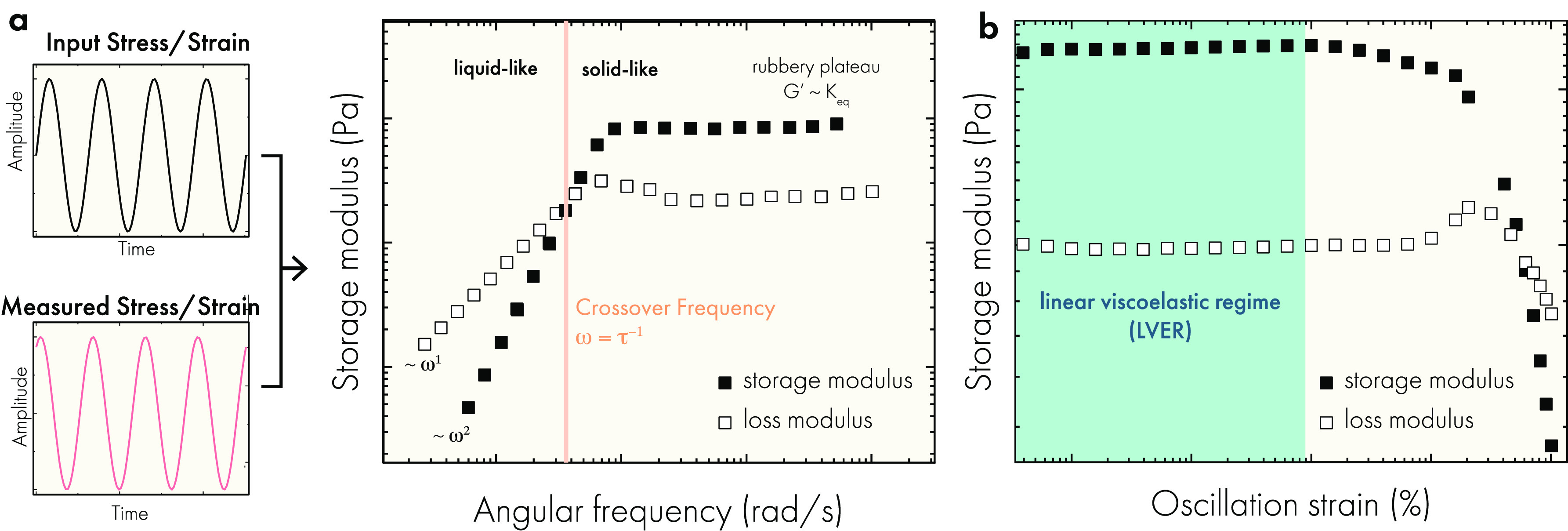
(a) Small amplitude oscillatory shear (SAOS) measurements impose a sinusoidal stress/strain and measure the sinusoidal response of the strain/stress, respectively. These data are typically represented using the storage and loss moduli. (b) SAOS measurements should be performed at a strain/stress amplitude within the linear viscoelastic regime, where the material response is constant. Data are original to this publication.

The viscoelasticity of a hydrogel is most often measured using dynamic mechanical analysis to measure the bulk elastic and viscous responses of a hydrogel to an imposed oscillatory shear strain or stress.^116^ Methods also exist to measure the viscoelasticity of hydrogels at various length scales, which may be important in cell-based applications where they interact with the hydrogel at different length scales than the bulk.^136,137^ In bulk oscillatory measurements, the amplitude and frequency of the imposed oscillations are varied, and the oscillatory response of the hydrogel is measured. Small amplitude oscillatory shear (SAOS) is the most common experimental method for measuring a hydrogel’s viscoelastic response. The oscillatory response measured in response to the oscillatory input is analyzed and typically represented through dynamic storage and loss moduli (Figure 7a), which describe the elastic and viscous responses of the hydrogel, respectively. When the storage modulus is greater than the loss modulus, the material is said to be solid-like. When the loss modulus is greater than the storage modulus, the material is said to be liquid-like. The storage and loss moduli are only well-defined when experiments are performed within the linear viscoelastic regime (Figure 7), where the hydrogel network responds linearly to the imposed strain or stress amplitude.

Frequency sweeps are performed at a constant strain or stress amplitude, and the frequency of the oscillation is varied to probe the material’s time-dependent viscoelasticity. For irreversible covalent hydrogels, solid-like behavior is observed for all frequencies without any significant frequency dependence due to the permanent cross-links in the network. For dynamic hydrogels, the response to oscillatory shear can be more complex, showing both solid and liquid like responses that depend on the frequency of oscillation. The point at which the storage modulus is equal to the loss modulus is the crossover frequency and denotes the transition between solid and liquid like states. In general, the viscoelastic response is a function of the thermodynamics and kinetics of the physical cross-link and network topology.^133,135,138−143^ Craig et al. have demonstrated for nonentangled physically cross-linked networks that the relaxation time (τ) of the hydrogel is equal to the dissociation rate of the physical cross-link.^142^ In a simple system, where the only cross-links originate from physical cross-links, the equilibrium constant (*K*_eq_) of the interaction describes the equilibrium concentration of cross-links and therefore the magnitude of the rubbery plateau. Though not discussed in detail here, stress relaxation experiments—where a constant deformation is applied and the temporal decrease in stress is monitored—are also a valuable experimental tool for measuring the relaxation time (τ) of dynamically cross-linked hydrogels.^115^ Stress relaxation experiments are especially useful when the relaxation time is longer than the measurable relaxation times in SAOS experiments.

Time sweep SAOS measurements—where the amplitude and frequency of oscillation are kept constant—are useful when measuring the transition of a hydrogel or its components from liquid to solid or vice versa, such as in the gelation of covalent and physically cross-linked materials. The oscillatory response is measured at a constant frequency and amplitude over an extended period of time. For irreversible covalent materials, mixing of two components can be performed immediately before measuring the materials viscoelastic response. The temporal evolution of the dynamic moduli reveals the kinetics of gelation, where the gelation point is assigned to the time at which the storage modulus surpasses the loss modulus at a crossover time. For dynamically cross-linked hydrogels, time sweep SAOS measurements are used to probe their self-healing behavior. The amplitude of the applied shear strain or stress is transitioned from low-to-high or high-to-low to probe the response of the dynamic cross-links. The temporal viscoelastic response of a dynamic hydrogel is measured to quantify the kinetics of recovery and degree of self-healing. This process is often alternated and repeated several times to demonstrate reversible self-healing of dynamically cross-linked hydrogels.

For injectable applications, dynamic hydrogels typically undergo transitions from a static equilibrium state to a nonlinear flow state and then return to a static equilibrium state. The properties of the hydrogels during and after these transitions influence their performance as injectable therapeutics. Measuring these properties, however, is challenging due to the transition from the linear to nonlinear regime. Nonlinear oscillatory shear measurements that go beyond the linear viscoelastic regime, such as large amplitude oscillatory shear (LAOS), have been a recent area of research focus.^86,144−150^ The storage and loss moduli become ill-defined in the nonlinear regime, and methods for quantifying a hydrogels’ response are more challenging. There have been recent advances in the analysis of nonlinear rheological data using Fourier transform analysis methods and a sequence of physical process methods, which provide more insight into the nonlinear properties of injectable biomaterials.

#### 2.4.2 Flow Rheology

**Figure 8:**
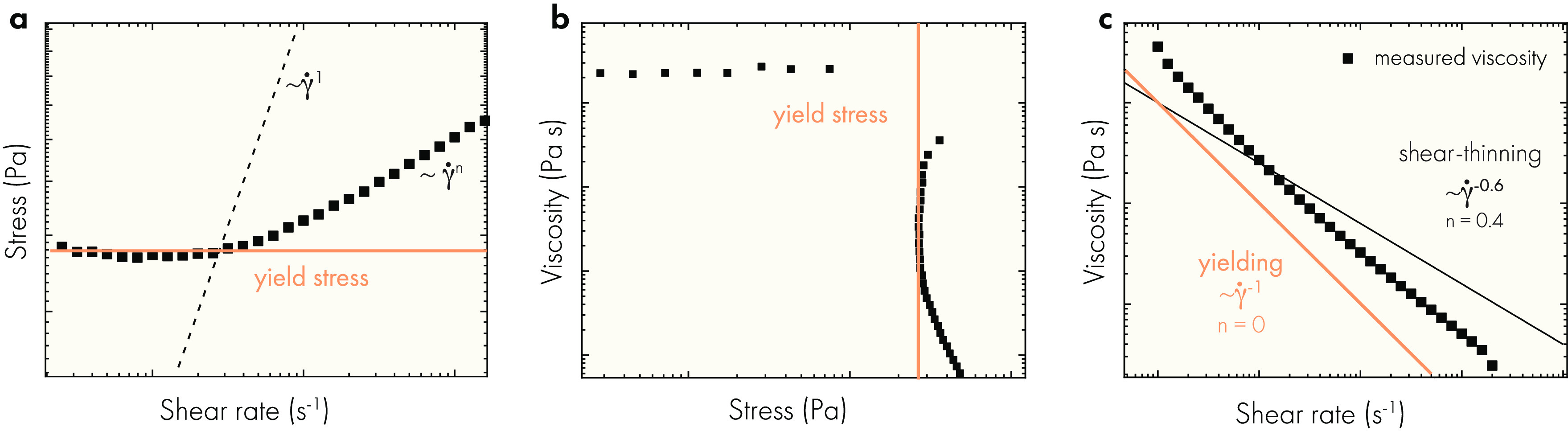
(a) Log–log flow curve for a shear-thinning yield stress fluid. A nonzero intercept with the stress axis is a fingerprint of yield stress fluids. Shear-thinning fluids scale according to a power-law (σ ∼ γ̇*^n^*) where *n* is between 0 and 1. A scaling exponent of one is the scaling of a Newtonian fluid. (b) Flow curve data plotted as viscosity versus stress readily reveals the yield stress where the viscosity drops by orders of magnitude for only a small increase in the stress. (c) Log–log plot of viscosity versus shear rate data shows the power law scaling of the viscosity with shear rate (η ∼ γ̇^*n*–1^). A scaling exponent of −1 (*n* = 0) is not shear-thinning but rather evidence of preyield behavior. Data are original to this publication.

The flow properties of a hydrogel are measured using a rheometer or capillary viscometer. In these instruments, a simple shear flow is applied to measure the relationship between the shear rate and shear stress of a fluid. This relationship is shown in a flow curve (and is extracted through an analysis of the imposed viscometric flows).^116^ A typical flow curve for a yielding, physically cross-linked hydrogel is shown in (Figure 8a). The viscosity is the ratio of the stress and shear rate and can be constant across shear rates (Newtonian) or be shear rate dependent (non-Newtonian). This section will discuss the acquisition of a steady state flow curve, introduce the important features of a flow curve for injectable hydrogels, and discuss the measurement transient thixotropic behaviors (time-dependent change in properties). Typical flow curves for physically cross-linked hydrogels show three distinct regimes: (1) preyield, (2) yielding, and (3) flow. We highlight that although discussion about a true yield stress has been a long contentious area of discourse in the scientific literature, the engineering reality of its effects is readily evident for injectable hydrogels.^130,151−153^

In a rheometer, an angular velocity is applied to a rotating geometry, and the resulting torque on the geometry is measured—or vice versa. With a known geometry, such as parallel plates or a cone-and-plate, the angular velocity is converted to shear rate and the torque is converted to stress. In a capillary viscometer, a constant flow rate is applied, and the pressure required to drive the flow is measured. The shear stress is determined from the geometry and pressure, and the shear rate is calculated from the geometry, flow rate, and pressure using the Weissenberg–Rabinowitsch–Mooney analysis.^116,154^ For Newtonian fluids, the shear rate is a function of the flow rate and channel geometry. For non-Newtonian fluids, the shear rate is also a function of the fluid’s viscosity in addition to the flow rate and channel geometry. Deciding between a rheometer and viscometer depends on the viscosity of the fluids being measured and on the shear rates that are of interest—outlined by Pipe et al.^154^ Generally, it is simpler to measure high-shear-rate flow curves using a capillary viscometer. In rheometers, there are significant challenges at high shear rates. The shear rate in rheometers is proportional to (gap size)^−1^ and is increased by decreasing the gap size between the two shearing surfaces. As the gap size is decreased to increase the shear rate, significant errors arise due to geometrical imperfections. Rheometers also suffer from radial migration of the sample and subsequent ejection of a sample at high shear rates. Capillary viscometers provide an alternative strategy for measuring the viscosity of fluids at high shear rates and use a closed capillary that is not prone to technical issues such as sample ejection. Regardless of the measurement technique used, the outcome is a measurement of the stress–shear rate relationship of a fluid.

The yield stress demarcates the minimum required stress necessary to induce flow for the fluid, and several strategies for measuring it have been developed.^153,155,156^ Here, we review the use of flow data to measure the yield stress. Using a stress vs shear rate curve (Figure 8a), the yield stress manifests as a nonzero intercept with the stress axis. The yield stress is then calculated using a Herschel–Bulkley model (eq 5) that is fit to the stress-shear rate data. Here, *σ*_*y*_ is the yield stress with units in Pascals, *n* is the shear-thinning parameter (unitless), and *K* is the consistency index with units in Pascal-seconds^*n*^. The modified Herschel–Bulkley model is often preferred because it yields fitting parameters with constant units and more intuitive meaning. The consistency index is replaced with γ̇_critical_, which is the critical shear rate with units in s^–1^ at which the flow stress is double that of the yield stress. Alternatively, some authors suggest plotting viscosity vs stress (Figure 8b), where a dramatic decrease of several orders of magnitude in the viscosity is observed for a small increase in the stress.^130,131,152,156−158^ Here, the stress at which the viscosity decreases is assigned as the yield stress. In plots of viscosity vs shear rate (Figure 8c), the preyield regime appears as shear-thinning. Before the yielding event, the stress is constant at increasing shear rates, resulting in a viscosity which appears to decrease.^115^ On a log–log plot of viscosity vs shear rate, this phenomenon is observed as a slope of −1. In practice, the visualization of rheological data showing flow data with plots of viscosity vs shear rate alone makes it challenging to understand important details about the rheological response of a dynamic hydrogel. For this reason, it is recommended that—at a minimum—both stress vs shear rate and viscosity vs shear rate data be shown when characterizing yield stress fluids. The flow curve (stress vs shear rate) shows yielding, while the viscosity versus shear rate plot of the flow regime more clearly shows the degree of shear-thinning for the hydrogel.
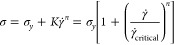
5

Postyield, physical hydrogels flow with shear-thinning behavior, where viscosity decreases as the shear rate is increased. Seen as a series of progressively decreasing slopes on a stress vs shear rate plot and as a negative, linear decline (slope = *n* – 1, where *n* is between 0 and 1) in a log-base plot of viscosity vs shear rate (Figure 8c).^115^ In a yielding hydrogel, the Hershel–Bulkley model provides information about the non-Newtonian viscosity of a hydrogel, where the consistency index and shear-thinning parameter describe the power law shear thinning of the hydrogel in flow. Alternatively, the flow portion of the viscosity versus shear rate curve can be fit to a power law (eq 1) to find the consistency index and shear-thinning parameter fits. It is critical that only the flow portion of the viscosity versus shear rate plot is used when fitting a power law to the rheological data of a dynamic hydrogel. In general, because it is difficult to distinguish between the preyield and flow regime, it is important to be cautious when demarcating the flow regime in viscosity vs shear rate plots before measuring the degree of shear thinning with a model fit (Figure 8c).

**Figure 9:**
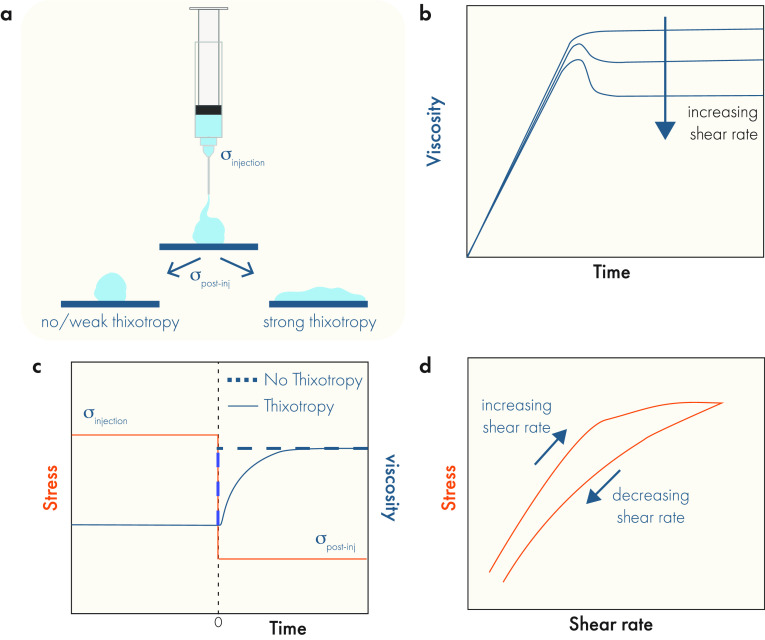
(a) Thixotropy affects the recovery of a material after being deformed. A weakly thixotropic material recovers internal structure and properties rapidly. A strongly thixotropic material has a delayed recovery in structure and properties. (b) Start up shear of a thixotropic fluid. Initially, thixotropic fluids show a similar response as the strain in the material accumulates, and it takes time to reach a steady state viscosity. For some structured materials, an overshoot is observed as the shear rate is increased. (c) Step-stress experiment, where the stress is instantaneously decreased. The viscosity for a thixotropic fluid increases slowly to its equilibrium value. A shear-thinning fluid without thixotropy would instantaneously reach its new equilibrium viscosity. (d) Flow curve hysteresis is often observed when measuring the flow curve for materials out of equilibrium.

When measuring a flow curve, it is critical to consider the effects of thixotropy and take appropriate precautions with test protocols. Intuitively, materials that are strongly thixotropic have a significant delay in restructuring, resulting in a transient response until equilibrium is reached during a deformation (Figure 9a). For dynamically cross-linked systems—which possess both solid-like and liquid-like behaviors—the dynamics of the cross-links and network often result in transient material response when changing the shear rate, especially before or near the yield point. It is common to observe an overshoot in the viscosity (Figure 9b) on the startup of shear as the network structure yields and breaks down to the new equilibrium state.^109,157^ At faster shear rates, the viscosity overshoot is more pronounced and depends on the yielding and relaxation behavior of the material. Figure 9c, shows the viscosity of a thixotropic material when the applied stress is instantly decreased (flow cessation). Instead of the viscosity increasing instantly, the viscosity slowly increases as the structure within the material rebuilds. The recovery of the viscosity can be fit to an exponential to determine the characteristic thixotropic time scales.^109^ The phenomena observed upon the sudden application or removal of shear shown in Figure 9b and c probe similar phenomena as described in self-healing SAOS experiments discussed above. Authors will often choose between either self-healing SAOS experiments or flow cessation experiments to demonstrate reversible self-healing.

Experimentally, thixotropy can significantly affect the acquisition of a flow curve, making it challenging to determine the equilibrium viscosities, shear-thinning, and yielding behavior of materials.^156,157^ Flow experiments that do not account for thixotropy are often irreproducible and can demonstrate significant hysteresis (Figure 9d). A simple strategy for measuring the equilibrium flow curve is to perform flow experiments using stepwise changes in the stress or shear rate and not ramped protocols.^109^ Stepwise experiments can be designed to apply a deformation until equilibrium is reached before taking a measurement.^115^

### 2.5 Outlook for Rheological Characterization of Injectable Hydrogels

In this section, we’ve highlighted the importance of functional constraints on the rheological behavior of dynamically cross-linked hydrogels. The constraints are often paradoxical, requiring higher yield stresses or viscosities for localization upon injection, yet also demand low viscosities to allow for facile injection. Here, we’ve reviewed the property–function relationship between the rheological properties of power-law shear-thinning fluids and the pressure required for injection. These relationships elucidate materials design targets for future injectable material platforms, specifically target viscosities which allow for facile injection at the shear rates relevant to the clinic. Designing these materials requires careful and rigorous characterization of viscoelastic and flow properties, which include viscoelasticity, shear-thinning, yielding, and thixotropy. We’ve briefly provided a survey of the more standard characterization techniques and point the reader to some reliable resources that provide a more rigorous description of the techniques. Most notably, stress relaxation and creep experiments are critical for understanding the long-time relaxation behaviors of materials and are not suitably characterized using SAOS. Together, this section provides the reader with a foundation to understand how the rheological behavior of existing hydrogels may translate to a desired function within their application. As more hydrogels are developed in the field of therapeutic delivery and new challenges arise, the property–function relationships shown here will enable more effective materials selection strategies to down-select materials and create rheological targets for new applications.

## 3 Hydrogels for Drug Delivery

Shortly after Wichterle and Lim described the first synthetic hydrogel,^1^ researchers began engineering hydrogels to deliver drugs locally and for extended periods of time.^159−161^ Hydrogel platforms for drug delivery have steadily evolved in their sophistication, expanding beyond synthetic, covalently cross-linked systems toward a hugely diverse set of biomaterials platforms. Alongside these exciting materials developments, the rise of mathematical models that describe the release of drugs from hydrogels and other biomaterials has become an important aspect for designing these systems and has been reviewed in depth.^162−164^ In particular, these models can guide the design of drug carriers so that they can meet the requirements of a particular application or they can help researchers elucidate the transport mechanisms that govern release kinetics from novel formulations. Many empirical, semiempirical, and numerical methods have been developed to describe transport from biomaterials. In particular, power law approaches, such as the Ritger–Peppas and Korsmeyer–Peppas models,^165,166^ have proven to be very useful for modeling controlled release of drug cargo from hydrogels.

By locally drugging target tissues, hydrogel drug carriers provide compelling safety benefits by reducing drug exposure in off-target tissue. Cancer therapies in particular stand to benefit considerably from this type of highly focused drug exposure.^167^ While hydrogels can locally focus drug exposure, they can also sustain a steady release rate of drugs over a prolonged period of time (e.g., hours, days, weeks, or months depending on the formulation). This sustained release of drugs is especially beneficial for reducing the number of doses required to treat a patient over time, which is promising for treating chronic diseases requiring lifelong medication, such as diabetes. Sustained release kinetics also appear to provide specific opportunities to enhance the efficacy of certain therapies, such as vaccines for infectious disease.^168^ Finally, the mechanical properties and overall biocompatibility of hydrogels allows them to integrate well into soft tissues and serve as the eventual scaffolding for endogenous cells as they degrade—traits that enhance their utility for a range of regenerative applications.

Overall, the value of carefully designed hydrogel drug carriers in biomedical applications is expansive and likely to be quite impactful. In particular, we focus on injectable hydrogel systems in this section, which for our purposes includes hydrogels that gel *in situ* as well as shear-thinning hydrogels. There are several recent reviews that cover this area in depth from either the materials or clinical perspective,^78,167,169−171^ and here we provide a hybrid view with an emphasis on the interdisciplinary nature of this type of research, in particular, the need for thoughtful materials design to be coupled with robust biological rationales and preclinical evaluation.

### 3.1 Foundations of Hydrogel Drug Delivery

**Figure 10:**
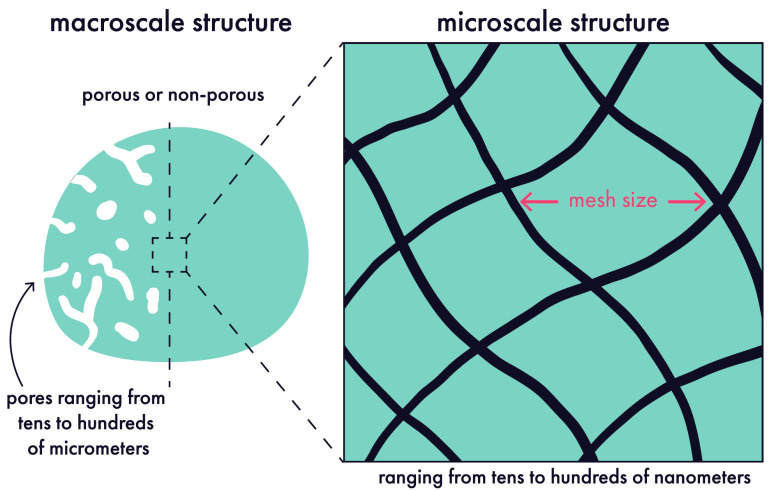
Important architectural features of hydrogels. Depending on the formulation, hydrogels can be highly porous or nonporous. Regardless of porosity, the polymer network that forms the hydrogel will exhibit a characteristic mesh size that has important implications for drug delivery. Original illustration.

**Figure 11:**
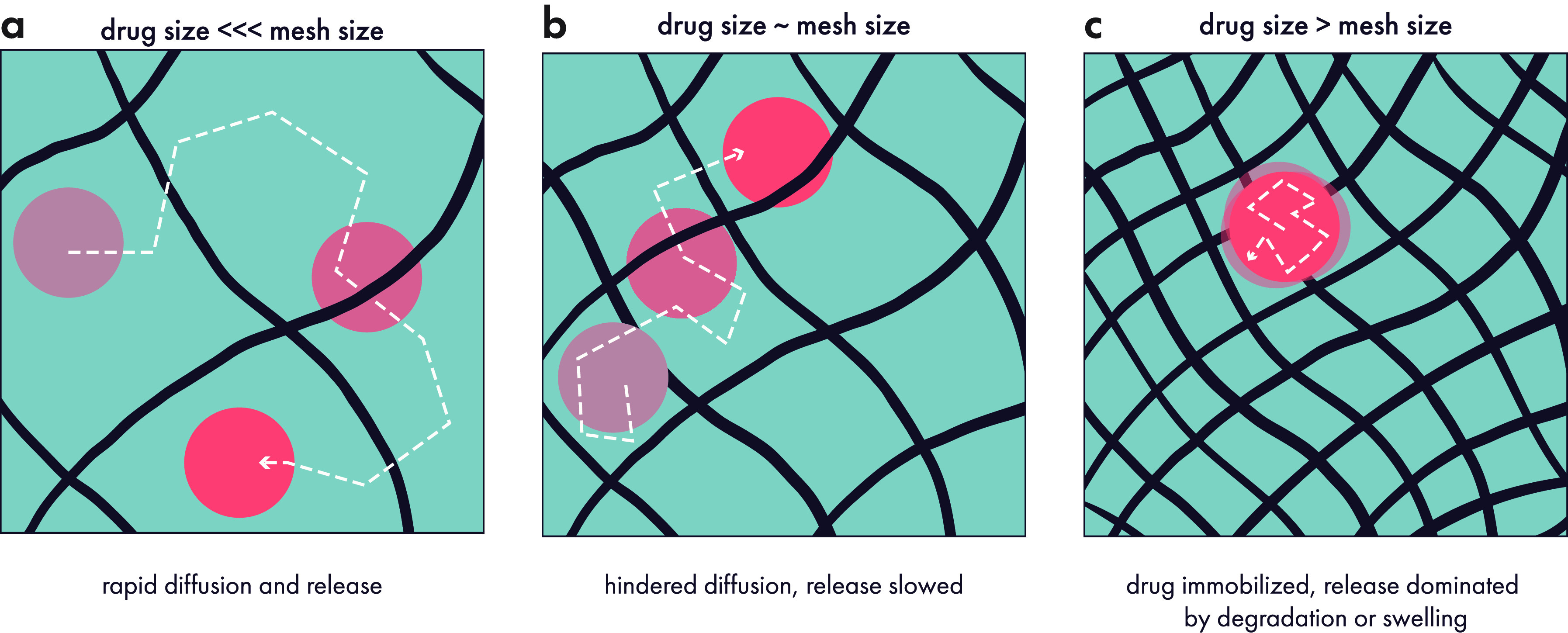
Drugs can be encapsulated into hydrogels and then passively released over time, with release kinetics dictated in large part by the ratio between drug size (hydrodynamic diameter) and hydrogel mesh size. (a) Drugs much smaller than the mesh size can freely diffuse in the free volume of the gel, and they usually rapidly exit the gel after administration with a characteristic “burst” release. (b) Drugs that are similar in size to the mesh will experience slowed diffusion. (c) Drugs much larger than the mesh are immobilized until the mesh size increases due to degradation, swelling, or mechanical forces. Original illustration.

The process of mass transport through hydrogels is essential for understanding how drug (and even cellular) cargo will move through these materials. Various properties influence mass transport, with some of the most salient being whether the hydrogel exhibits macroscopic architecture, such as porosity, and how tightly cross-linked the polymer network is, which gives rise to the hydrogel mesh size (Figure 10). Critically, the movement of cargo inside of a hydrogel depends strongly on the relationship between the cargo’s hydrodynamic diameter and the hydrogel mesh size (Figure 11). Depending on the ratio between these two features, cargo release from hydrogels may be diffusion-dependent, erosion-dependent, or dependent on both mechanisms. In general, diffusion-dependent release occurs over shorter timeframes, ranging from hours to days, while erosion-based approaches can extend release out to weeks or months.^172^ Prior to delving into cargo-specific considerations, we will briefly review the behavior of cargo in hydrogels as a function of hydrogel mesh size and erosion kinetics.

In many hydrogel systems, the mesh size can be tuned to regulate the release of cargo, with reported sizes ranging from single-digit to hundreds of nanometers.^172^ Intuitively, if the mesh size is much larger than a drug’s hydrodynamic diameter, then the drug can freely diffuse through the network with minimal steric hindrance. Under these conditions, diffusion is the dominant mechanism for release, and the diffusivity of the cargo inside the gel may be similar to its diffusivity in bulk solution. When this is the case, drug release can be quite rapid, with the cargo releasing completely from the hydrogel in hours or days. However, the diffusion of molecules can be strongly affected by nearby surfaces and substrates, which has led to active research on modeling and understanding the concept of hindered diffusion.^173,174^ As a result, even if a drug is smaller than the effective mesh size of a hydrogel, it may very well diffuse more slowly than it would if it were simply in free solution. If the hydrogel mesh size is comparable to the hydrodynamic diameter of the drug, then diffusion can be slowed down considerably, but it still remains an important contributor to release, alongside erosion kinetics (Figure 11b).

In contrast, if the mesh size is smaller than the hydrodynamic diameter of the cargo, then the cargo is sterically hindered and cannot move through the hydrogel—leaving it essentially trapped (Figure 11c). In these cases, drug release is dominated by mechanical disruption, gel erosion, or swelling behaviors.^175,176^ Only when the surrounding gel has sufficiently broken down, effectively increasing the mesh size in the vicinity of the cargo, can the drug diffuse away from the carrier. In these cases, erosion kinetics become a critical determinant of drug release kinetics, and there are several ways that erosion mechanisms can be engineered to control drug release.^172^

Biomedical hydrogels are generally designed to erode or degrade under physiological conditions, through mechanisms such as hydrolysis or enzymatic digestion, to break down the polymer network into resorbable, metabolizable, or excretable base components. If a hydrophilic hydrogel is susceptible to hydrolytic degradation (e.g., polyesters), then it generally undergoes bulk erosion—that is that the network throughout the gel is simultaneously degrading at a similar rate.^177,178^ Bulk erosion could also occur through other mechanisms, such as with an enzymatically degraded gel, provided the mesh size of the hydrogel permits rapid penetration of the enzyme from the exterior. Alternatively, hydrogels can also undergo surface erosion when the exterior of the gel breaks down more quickly than the interior bulk.^177,178^ This can occur when the molecular agents that degrade the gel (e.g., water, enzymes) diffuse into the bulk slowly relative to the rate of surface erosion. This can occur with hydrogels bearing hydrophobic components that slow down the rate of water penetration into the bulk or when the mesh size is much smaller than the size of the enzymes that are responsible for breaking down the network. Regardless of the mechanism of erosion, these behaviors can be readily modeled to predict erosion-dependent release kinetics.^179^ Overall, the mechanisms of hydrogel erosion are important considerations when designing a drug carrier, in particular when delivering large cargoes such as nano- or microparticles.

**Figure 12:**
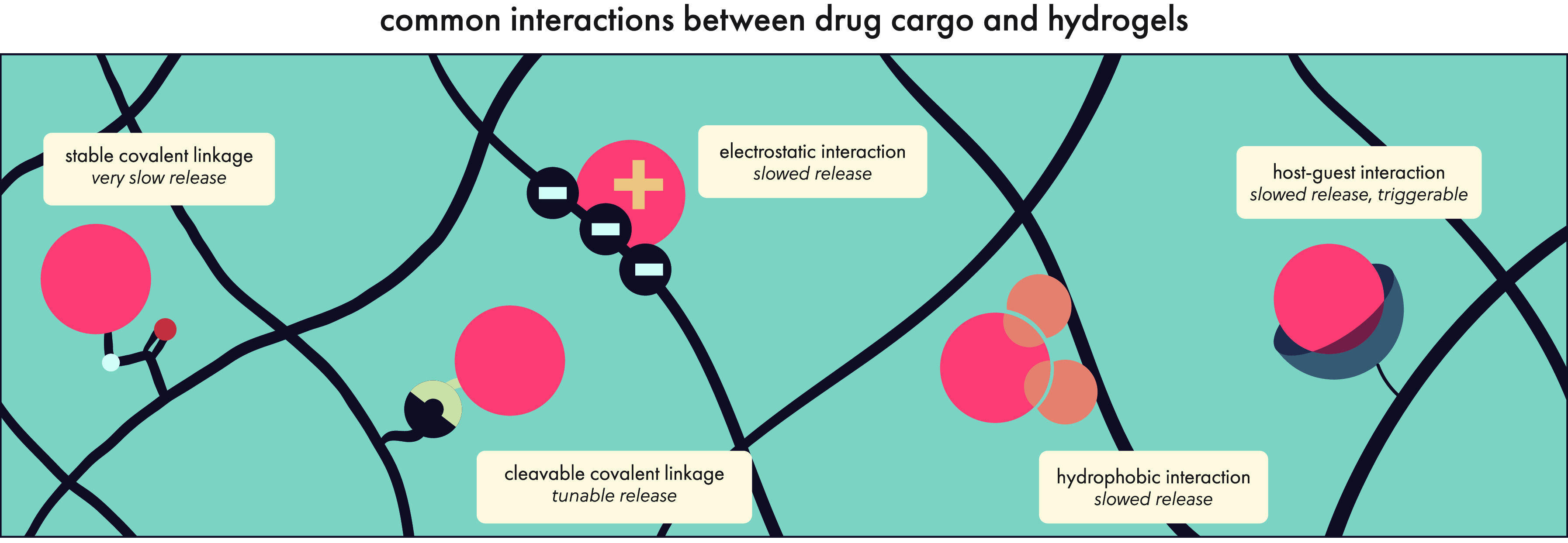
Drugs can have both intentional and unintentional interactions with the hydrogel network based on the physical and chemical characteristics of each, and these interactions can have important consequences for drug release kinetics. In later sections, we will discuss how these interactions can be leveraged for controlled release of specific cargo. Original illustration.

In many instances, the cargoes that are delivered for biomedical applications are drugs smaller than 15 nm—small molecules or compact proteins.^180^ We will delve into the specific considerations for the different types of cargoes in the following sections, but in many cases hydrogel delivery of biomedical drugs through passive means (e.g., physical encapsulation and subsequent release) will be strongly diffusion-dependent. Therefore, there is considerable benefit to being able to predict or model the diffusion of cargoes within different types of hydrogels. There are numerous models that capture important aspects of this behavior, such as hydrodynamic theory,^181^ free volume theory,^182^ and obstruction theory.^183^ The literature on these models is extensive,^162−164,184^ and here we provide basic summaries of their underlying assumptions. In general, these theories assume the drug (or solute) is a perfect hard sphere in the aqueous bulk phase of the hydrogel. Most general models assume negligible hydrophobic, electrostatic, or van der Waals forces. However, it should be noted that in certain drug/hydrogel combinations these interactions can strongly impact mass diffusion (Figure 12).^185^ Hydrodynamic theory is focused on the effects of friction between cargoes and the hydrogel, which is considered to be a fluid (instead of a solid) in this particular model. The free volume theory looks to model diffusion by assuming cargo is transported through the dynamic open spaces between the molecules that form the hydrogel.^186^ Obstruction theory models the polymer mesh as a physical barrier that hinders the diffusion of the drug through the bulk phase.^187^ More recently, our group developed a model that combines these three approaches, which we call the Multiscale Diffusion Model (MSDM).^188^ The MSDM notably reconciles both theoretical and experimental inconsistencies between the prior three models,^184^ providing a more accurate prediction of cargo transport in a variety of PEG and alginate hydrogels.^188^ Overall, theoretical models provide researchers with a critical tool for designing hydrogels before coming to the bench, helping to minimize costly trial-and-error optimization. Nevertheless, there is an outstanding need for models that fully capture the complexities of drug delivery, including drug–hydrogel interactions and drugs of complex shape (e.g., elongated biopolymers such as DNA). For readers interested in further details, we refer them to several excellent and comprehensive reviews on the physics and modeling of drug diffusion through hydrogels.^162−164,184^

So far, we have seen that the relationship between drug size and mesh size can help predict a great deal about drug release kinetics. If taken into account during the design stage, hydrogels can be formulated to feature a mesh size that is more likely to yield desirable release kinetics. To this end, hydrogel mesh size can usually be made smaller by increasing the polymer content and/or cross-linking density and vice versa. However, while tuning the mesh size can be relatively straightforward in more traditional, covalently cross-linked systems, certain precautions must be taken when looking to leverage these concepts in dynamic hydrogels. This is due to the reversible nature of the cross-links in dynamic hydrogels, which can essentially lead to the reversible opening and closing of paths for entrapped macromolecules diffusing through the hydrogel. In these instances, the time scales for the formation and dissociation of these cross-links may play an important role in governing diffusion. In addition, noninjectable systems can increase or decrease polymer concentration and/or cross-link density to tune the mesh size, generally without jeopardizing the downstream applications of the hydrogel (unless those applications depend on mechanical properties, which can be altered by these changes). In contrast, changes to cross-link density may have a detrimental impact on the injectability of dynamic hydrogels, particularly when scaled to clinically relevant geometries, as discussed previously. As a result, novel strategies for regulating drug release, and in particular small molecule drugs, have been important for designing injectable hydrogels for sustained release applications.

### 3.2 Considerations for Small Molecule Delivery

Drug carriers aim to improve the efficacy of their cargo by delivering more of the active drug to its site of action within target tissues. Simultaneously, these carriers should reduce the exposure to the drug in off-target tissues, where it can cause toxic side effects. Nanoparticle carriers, another exciting materials approach to solving biomedical challenges, are designed to accomplish this by encapsulating small molecules through various passive and chemical strategies.^189^ Nanoparticles then must protect their cargo while navigating the body to reach a target tissue. This is a considerably complex task, and for now nanoparticle drug delivery still leads to extensive accumulation of drug in filtration organs such as the liver and spleen and has had limited success in specific tissue targeting.^190^ In contrast, hydrogels sidestep the challenge of navigating through the body by being administered directly at the target site. This is now easily achievable with injectable hydrogels, which can be administered to diseased tissues using a minimally invasive approach. While this local drug delivery has limited utility for the treatment of a disseminated disease, such as metastatic cancer, it has a great deal of potential in treating localized disease or injured tissues. As we will detail in later sections, hydrogels offer some unique advantages for locally interfacing with the immune system to orchestrate systemic immune responses.

All this is to say that the design of contemporary hydrogel drug carriers is heavily focused on two properties: (i) injectability, either through shear-thinning or shape-memory properties or triggered *in situ* sol–gel transitions, and (ii) tuning parameters that govern the release of cargo. Obtaining a high degree of control over drug release is particularly challenging with small molecule drugs, the focus of this section. Due to their small size, small molecule drugs present a challenge for sustained release strategies, many of which are based on passive diffusion approaches that involve tuning hydrogel mesh sizes. As a result, a rapid burst-release of drug is a common problem when delivering this class of drug.^191^ Large bursts introduce safety concerns by potentially increasing drug exposure to dangerous levels in the target tissue. Burst release can also saturate local tissues with drug, allowing excess drug to escape into systemic circulation where it can affect off-target organs. The subsequent steady state release of the remaining drug after a burst may also be too little to maintain the target tissue within the drug’s therapeutic window.

Overall, the field has worked diligently to exert greater control over the release of small molecule drugs. Because mesh sizes cannot generally restrict the diffusion of small molecules through hydrogels, the effect of polymer–drug interactions with the matrix can make a significant impact on release kinetics. These interactions can consist of electrostatic, hydrophobic, hydrogen-bond, van der Waals, or other specific and nonspecific interactions. Nonspecific interactions, for example, may explain why increasing the polymer content of certain hydrogels can attenuate the burst release of hydrophilic small molecules,^192^ despite the fact that the mesh sizes remain much larger than the size of the cargo. Incorporating polyelectrolytes into hydrogels can similarly slow down the release of oppositely charged small molecules, greatly reducing burst release of the cargo.^193^ Although hydrophobic small molecules exhibit very slow steady state release kinetics from hydrogels, they still exhibit a burst release (albeit smaller than their hydrophobic counterparts).^192^ Hydrogels that feature hydrophobic pockets enabling host–guest interactions within the polymer network can reduce the extent of this burst release, as has been demonstrated by cyclodextrin-functionalized polymer networks.^194,195^ In a similar vein, hydrophobic nanoparticles can be used to encapsulate these drugs and entrap them within a hydrophilic hydrogel network.^196^

**Figure 13:**
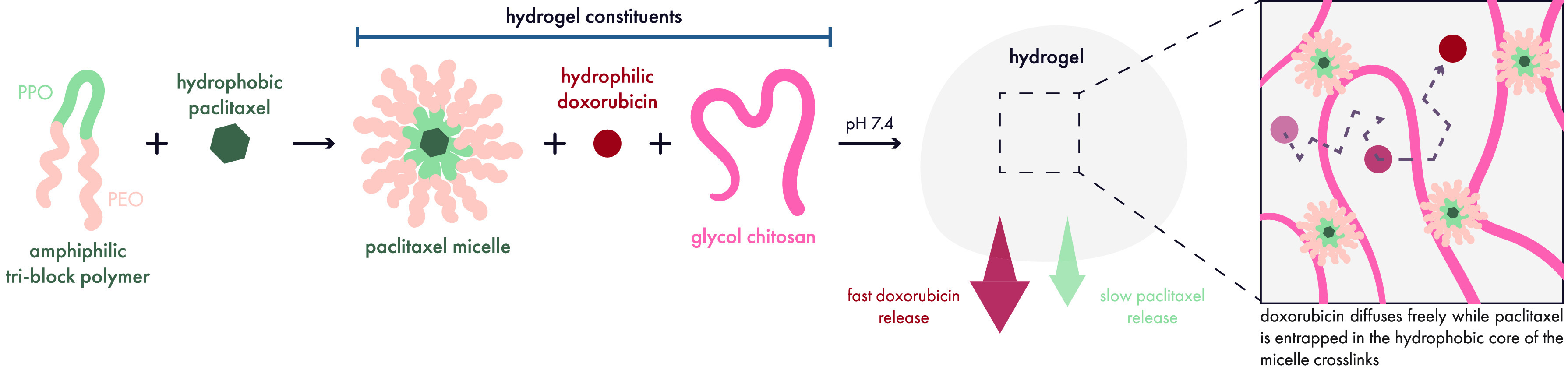
Strategies for sustained release of small molecule drugs: leverage differences in solubility. When delivering multiple small molecule drugs, it can be advantageous to have them release at different rates, especially in cases where their synergy depends on staggered or scheduled release. Researchers used this approach using a hydrogel with distinct hydrophobic and hydrophilic compartments that could house paclitaxel and doxorubicin, respectively. The water-soluble doxorubicin releases through diffusion-dominated kinetics, while the paclitaxel releases more slowly as the hydrogel erodes. Original illustration inspired from the work of Yang and co-workers.^197^

These technologies have important implications for the clinic, as hydrogels that locally deliver small molecule drugs and avoid systemic exposure have been shown to maintain efficacy while reducing toxicity. Along these lines, Yang and co-workers demonstrated that a pH/temperature-sensitive hydrogel could reduce the toxicity of a common chemotherapy regimen that combines hydrophilic doxorubicin and hydrophobic paclitaxel (Figure 13).^197^ This study highlights how the solubility of small molecule cargo directly influences its release profile, with the water-soluble doxorubicin following a typical burst-release followed by a slower sustained release. In contrast, the hydrophobic paclitaxel was released quite slowly, possibly on the time scale of gel degradation. An important consideration from this study is that the cargo influenced the gelation time and the mechanical properties of the resulting gels, and the effect was also cargo specific. Doxorubicin drove quicker gelation and resulted in higher modulus gels while paclitaxel slowed gelation and decreased the modulus of the resulting gels. In the end, the effects tended to cancel each other out when the drugs were combined, but the impact of cargo on the mechanical properties of the hydrogel can nonetheless affect critical factors such as *in vivo* erosion and release kinetics.

While the release of drugs from this hydrogel was primarily driven by simple passive diffusion, the effect was sufficient to provide a considerable improvement in off-target side effects. Mice treated with doxorubicin-gels had ca. 40-fold lower peak drug concentrations in the blood when compared to mice receiving bolus treatments. Potentially due to the lower systemic exposure, gel-treated mice were protected from treatment associated acute weight loss and cardiotoxicity. In addition to the improved safety profile, gel-delivery made this treatment regimen even more effective in a murine model of melanoma, compared to bolus treatments. This outcome may be partially due to schedule-dependent synergy between doxorubicin and paclitaxel, where optimal efficacy requires doxorubicin release to precede paclitaxel.^198,199^ Due to the intrinsic differences in solubility between these cargo, doxorubicin naturally released much more quickly from the hydrogel than hydrophobic paclitaxel, creating a rudimentary but effective staged-release effect. The capacity for hydrogels to confer this kind of improvement in safety and efficacy is likely to continue growing as materials scientists develop platforms with increasingly sophisticated controlled release mechanisms.

Not all small molecule drugs will possess useful physicochemical properties that can be taken advantage of by strategies seeking to increase drug–polymer interactions. In these cases, there are limited options for designing a hydrogel carrier, but one powerful technique has been to use molecular imprinting to fabricate hydrogels that are tailor-made to bind to a specific molecule.^200,201^ With this approach, hydrogels are synthesized in the presence of a “template molecule” that can later be removed. The cavity left behind from the template exhibits a high degree of affinity for the actual cargo molecule, in a sense creating an artificial binding pocket. Unfortunately, the need to retain this specific shape has precluded this technique from being implemented in injectable hydrogels so far.^202^ However, the ability to molecularly imprint nanoparticles might provide an avenue for incorporating this technology into injectable hydrogels in the future.^203^

Leveraging intrinsic drug–-polymer interactions can be a powerful tool in developing hydrogel carriers for a variety of small molecules, reducing burst release and at times extending the period release to achieve particular biomedical goals. Nevertheless, the release kinetics of these systems is often diffusion-dominated and can lead to faster release than what is desired, which has led to alternative strategies that go beyond passive release mechanisms. One technique is to tether drugs directly to the hydrogel network, either through irreversible covalent attachment or a labile linkage.^204−207^ This type of approach can significantly extend drug release and minimize burst effects, with release kinetics governed primarily by the degradation kinetics of the hydrogel. In some instances, it can also introduce stimuli-responsive release of drugs, if, for example, the linkages are cleavable by specific environmental factors such as pH or expression of a specific enzyme.^45^ Alternatively, light and heat-cleavable linkages open the door to exogenously triggered drug release.^207,208^ Overall, these strategies provide significantly more control over the release rate of small molecules, but they do introduce their own complexities. For example, chemical modification of small molecule drugs may affect their biological properties in ways that are challenging to predict. The kinetics of stimuli-responsive labile linkages may also be difficult to predict, especially if it is based on the endogenous expression of a particular enzyme or protein. For exogenously triggered cleavage of linkers, stimuli such as light and heat may be difficult to apply in a translational setting (e.g., penetration depth limitations of light). These are issues that highlight the interdisciplinary nature of this endeavor, which would benefit from collaboration between clinicians, biologists, medicinal chemists, and materials scientists.

**Figure 14:**
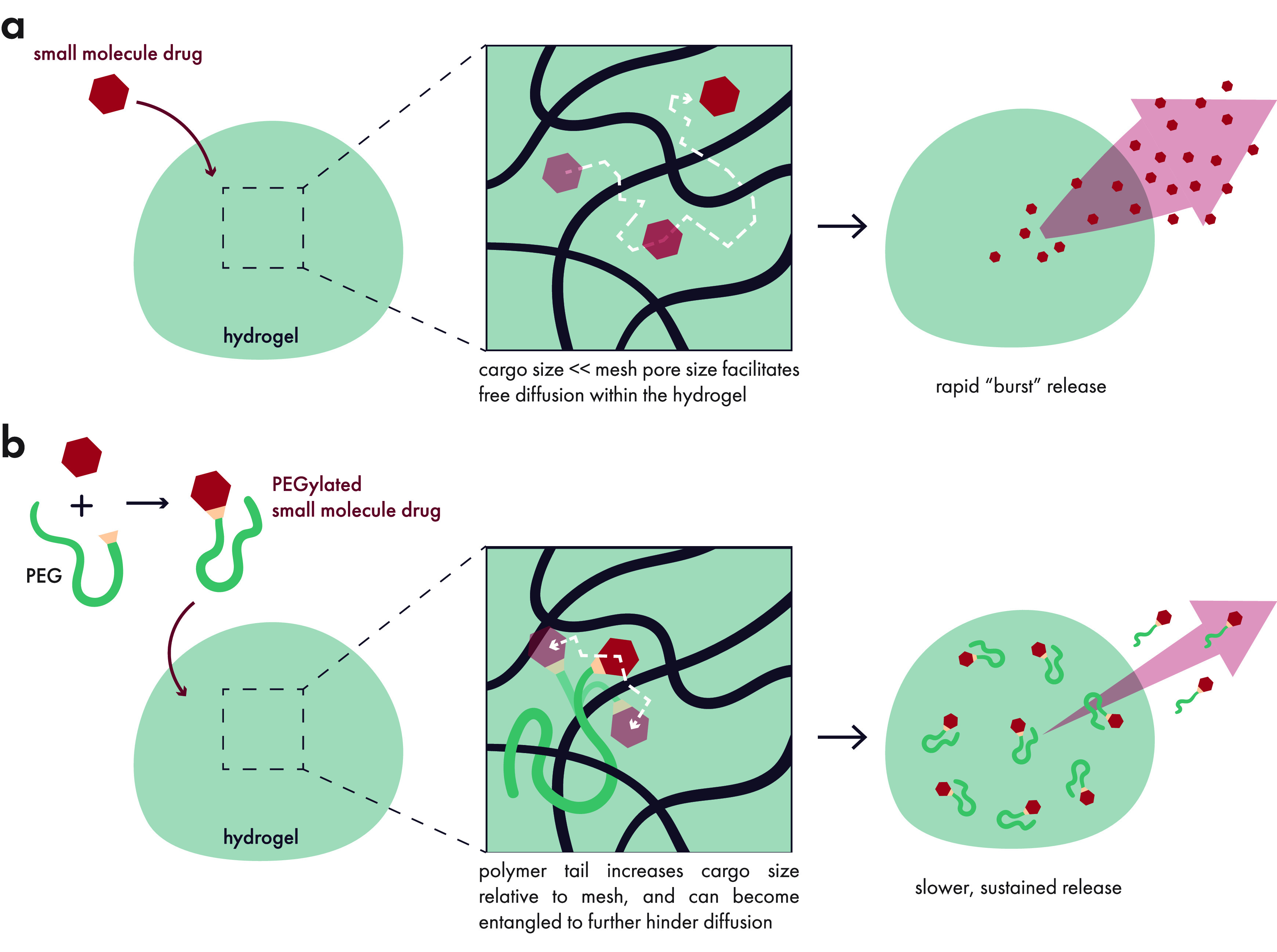
Strategies for sustained release of small molecule drugs: make it bigger. Attaching PEG to small molecule drugs and other therapeutic cargo can improve drug pharmacokinetics and solubility, but it also can make cargo significantly larger. Taking advantage of these approaches, researchers can modify their cargo to hinder its diffusion through a hydrogel vehicle. Original illustration inspired from the work of Ding and co-workers.^209^

An alternative approach to governing small molecule drug release has been to engineer the molecules into a form that interacts with the hydrogel either physically, chemically, or supramolecularly (Figure 14). For example, Ding and co-workers took advantage of methods for conjugating poly(ethylene glycol) (PEG) to camptothecin, a chemotherapy drug, which considerably increased the size of the cargo.^209^ This process, commonly referred to as PEGylation, is a well-documented approach for improving the solubility of small molecule drugs and proteins and increasing their size.^205,210^ It provides significant benefits for a variety of drugs, particularly in extending the circulatory half-life of systemically administered drugs and altering their biodistribution.^211^ In this study, mixing a PEGylated form of camptothecin with a triblock copolymer comprising poly(lactic acid-*co*-glycolic acid) and PEG (PLGA-PEG-PLGA) generated an injectable solution that gelled at physiological temperatures. The resulting hydrogel provided a depot for release of PEGylated camptothecin, which could be tuned by changing the relative size of the polymer blocks and their relative concentrations. When injected subcutaneously in S-180 sarcoma-bearing mice, the hydrogels slowed tumor growth despite being distant from the actual tumor, likely by maintaining the therapeutic levels of PEGylated camptothecin in the blood. Assuming that maintaining these levels of camptothecin is tolerable, this and similar hydrogels could replace the long infusions characteristic of chemotherapy and have efficacy against widely disseminated cancers. Future studies should evaluate the efficacy of peri or intratumoral hydrogel injection, which may achieve therapeutic effects at lower doses and mitigate toxic side effects.^212^

In this system, the long bulky PEG arm of the camptothecin was entangled with the polymer mesh of the hydrogel, slowing its diffusion out of the gel. Interestingly, the inclusion of PEGylated camptothecin had a considerable impact on the mechanical properties of the PEG-PLGA-PEG hydrogels. Namely, the drug lowered the sol–gel transition temperature and increased the viscosity of the sol. This finding introduces an important consideration for how the cargo itself may interfere (potentially beneficially or detrimentally) with the dynamic self-assembly behaviors that drive gelation. For example, Shim et al. reported how the chemotherapy drug paclitaxel decreased the sol–gel transition temperature of another temperature-sensitive hydrogel formulation in a dose-dependent manner,^213^ possibly due to a salting-out effect.^214,215^

**Figure 15:**
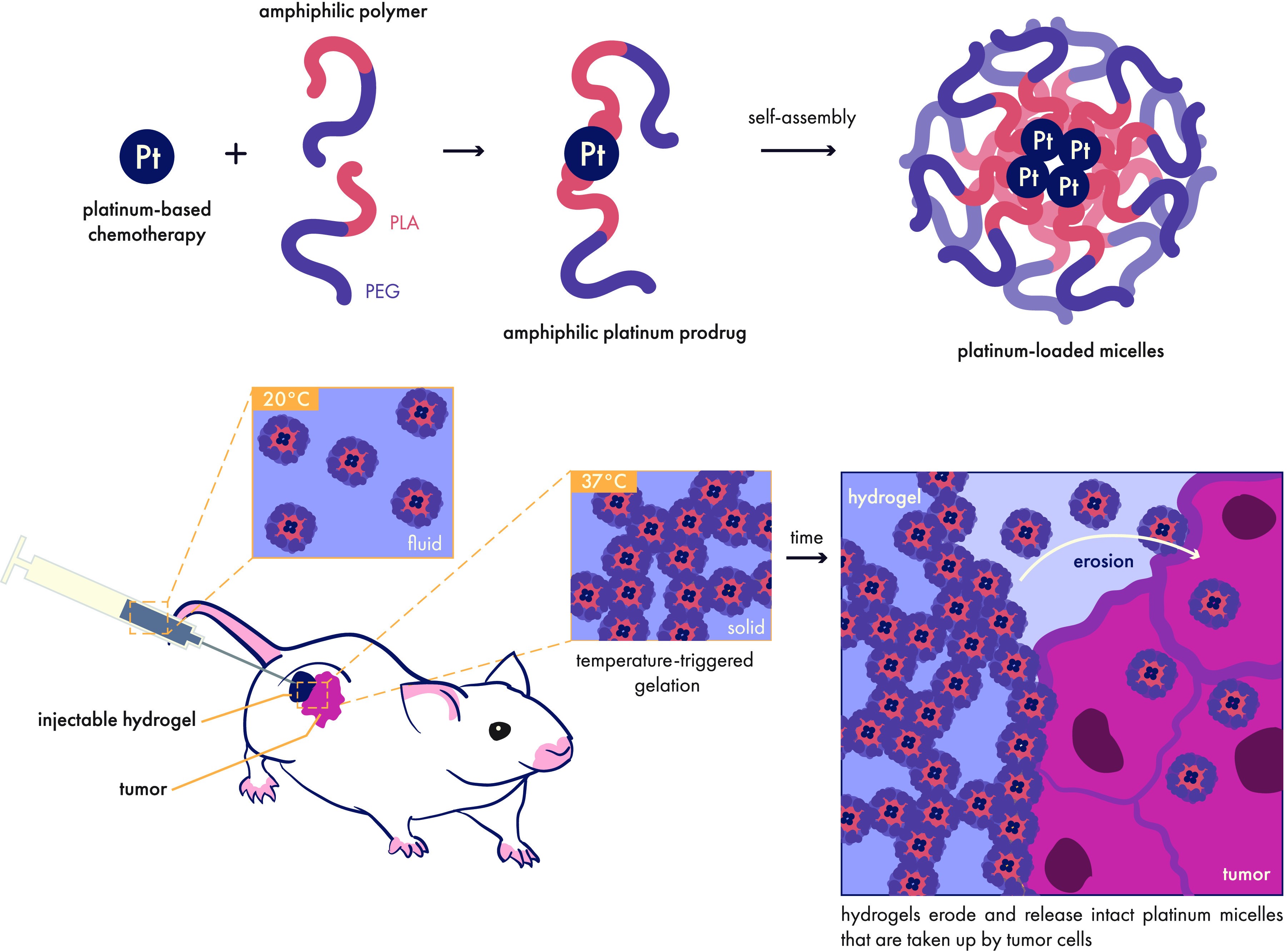
Strategies for sustained release of small molecule drugs: incorporate it into the cross-linked network. Certain small molecule drugs can be modified into pro-drug forms by attachment to polymers. Taking this a step further, attaching amphiphilic block copolymers to cargo can create building blocks that self-assemble into useful nanostructures. Researchers used this principle to generate a platinum drug derivative that self-assembled into a temperature-sensitive micelle. At physiological temperatures, these micelles formed a network and spontaneously generated hydrogels that released platinum-loaded nanoparticles over the course of a month. Original illustration inspired from the work of Ding and co-workers.^216^

The impact of cargo on the mechanical properties of a gel is especially critical in supramolecular hydrogel systems that incorporate their cargo into their building blocks, a strategy which Ding and co-workers used to develop a sustained release system for a derivative of cisplatin, another common chemotherapy drug (Figure 15).^216^ The Pt(IV) derivative of cisplatin was conjugated to two PEG-*b*-poly(d,l-lactide) (PEG-PLA) block copolymers to form a triblock macromolecule. This amphiphilic polymer self-assembled into micelles with the platinum prodrug contained within the hydrophobic core. At physiological temperatures, the micelle solution undergoes a sol–gel transition, yielding a long-lasting depot of platinum–drug loaded micelles. As the gel degrades, intact micelles are released which can be broken down into the active Pt(II) drug by intracellular reducing agents. By incorporating the platinum prodrug into the building blocks of this hydrogel, this hydrogel significantly slowed the release of a very small molecule drug (∼75% released *in vitro* in 40 days), which would otherwise rapidly diffuse out of a passive-release system (75% released *in vitro* in <5 h). However, inclusion of the drug into the building blocks of this supramolecular system had significant effects on hydrogel formation. In this case, the effect was quite beneficial—the inclusion of the Pt(IV) into the hydrophobic region of the triblock decreased the sol–gel transition sufficiently to form gels under physiological conditions. In contrast, drug-free triblock PEG-PLA-PEG systems gelled at ∼50 °C, which would hardly be useful for biomedical applications. While this approach is promising for extending the release of a fundamental chemotherapy drug, *in vivo* studies on release rates and efficacy still need to be carried out. In particular, it will be important to determine if such a drug delivery system might mitigate the traumatic side effects that accompany platinum-based chemotherapies.^217^

While the prior approaches focus on delivery of encapsulated small molecules, one innovative approach by Kibbe and co-workers uses a hydrogel system to generate therapeutic small molecules *in situ*.^218^ By incorporating nitric oxide (NO) donors into a peptide nanofiber hydrogel platform, this group produced an injectable depot of NO to locally inhibit neointimal hyperplasia, a condition which complicates treatment of cardiovascular disease. Notably, in developing this hydrogel, four unique NO-donor candidates were tested, two of which prevented gelation. Again, we see that the properties of cargo can exert potent and difficult-to-predict effects on the forces driving gelation, which led to the discontinuation of two donors. And while the remaining two NO donors yielded hydrogels, only one of the donors (PROLI/NO) could inhibit neointimal hyperplasia in a rat model. Importantly, the PROLI/NO formulation was successful *in vivo* despite underperforming in earlier *in vitro* assays, highlighting the need to carry forward these studies into relevant preclinical models.

### 3.3 Considerations for Nucleic Acid Delivery

Nucleic acid-based therapies include DNA and RNA that encode beneficial proteins,^219^ as well as microRNA and siRNA, which can silence expression of specific genes.^220^ Synthetic biologists have further advanced the capabilities of nucleic acid cargoes with stimuli-responsive and self-replicating constructs known as replicons.^221^ More recently, gene therapies have grown to include CRISPR-based systems which can precisely edit the host genome.^222,223^ Short DNA and RNA oligomers known as aptamers have also been developed, with the capability to bind to and regulate specific targets with specificity and affinity comparable to antibodies.^224^ Additionally, several immunogenic nucleic acids that are agoinsts for toll-like receptors (TLR), including CpG (TLR9 agonist), poly(I:C) (TLR3 and RIG1 agonist), and ssRNA (TLR7/8 agonists), have become important adjuvants for a variety of immunotherapies.^225^ In general, all of these biopolymers share similar physicochemical traits due to the conserved phosphatidyl backbone of nucleic acids, so in general these cargo are relatively stiff (particularly in the double-stranded form) and negatively charged species,^226−228^ which complicates their delivery through cell membranes. They are also susceptible to numerous endogenous enzymes that quickly degrade extracellular or otherwise “out of place” nucleic acids. Because the information stored in nucleic acids is variable and length-dependent, the overall size of therapeutic cargo can vary from the tens of kilodaltons (e.g., siRNAs) to the megadalton range (plasmid DNA or poly(I:C)). As a result, nucleic acid therapeutics are sensitive and difficult to deliver to their site of action, major obstacles for their clinical translation.

**Figure 16:**
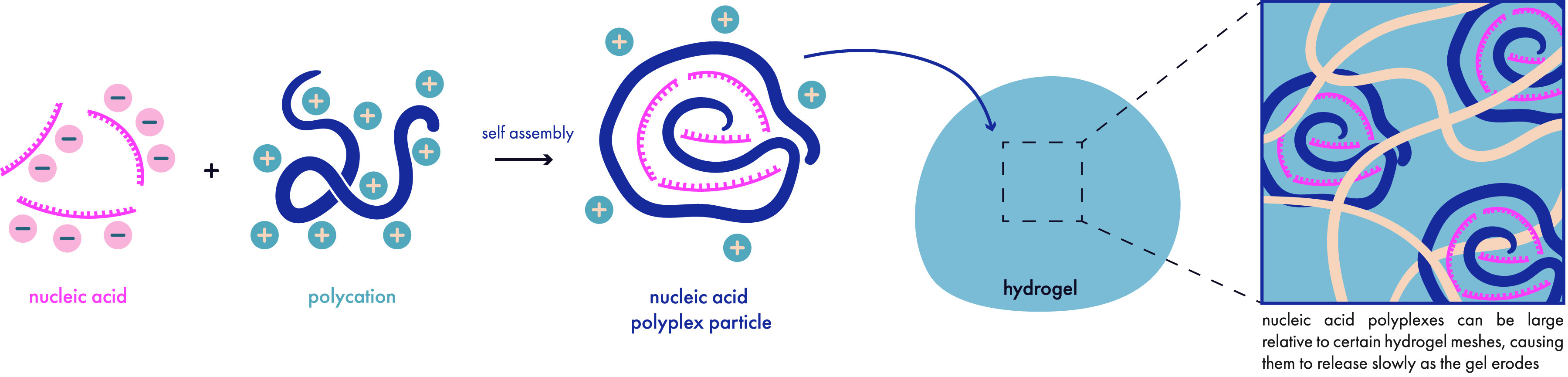
Strategies for sustained release of nucleic acids: preload them into protective polyplexes. While therapeutic nucleic acids can have variable sizes and mechanical properties, they are all strongly anionic. As a result, complexation with a suitable polycation can yield nano to micron scale polyplex particles that not only protect nucleic acid from premature degradation but also generate larger structures that are more easily retained by hydrogels. Once released, the cationic constituent of the polyplex can also facilitate entry into cells and cytoplasmic release of cargo. Original illustration.

Fortunately, electrostatic interactions with polycations have proven to be a useful and effective way to condense nucleic acid cargo into nano- and microsized particles that protect cargo from premature degradation (Figure 16).^229,230^ Electrostatic complexation with supramolecular building blocks (e.g, peptide amphiphiles and phospholipids) can also yield self-assembled particle carriers.^231,232^ In addition to protecting nucleic acids from premature degradation, particle carriers also have cell-penetrating capabilities that can help to deliver cargo to the cytoplasm or nucleus, where they can carry out their therapeutic function. While electrostatically assembled polyplexes can be effective, without further modification they have overall poor pharmacokinetic properties and poor tissue-targeting, as well as issues with toxicity and safety.^189,233^ But by formulating hydrogel carriers of nucleic acid polyplexes, these therapeutic particles can be delivered locally to target tissues to resolve both of these issues.

Chen and co-workers used this approach with a PLGA-PEG-PLGA thermosensitive hydrogel that safely delivered shRNA against the tumor oncogene PLK1.^234^ PLK1 shRNA was complexed with a polylysine-modified polyethylenimine, producing a roughly 100 nm particle,^235^ which is sufficiently large to tune the release rate based on the porosity of the hydrogel carrier. The local release of shRNA led to a decrease in tumoral PLK1 expression. The authors also explored simultaneous delivery of the chemotherapy drug doxorubicin and found that the combination therapy led to synergistic antitumor effects via activation of a G2/M cell cycle checkpoint. Importantly, hydrogel combination therapy avoided off-target cardiotoxicity typically associated with doxorubicin and saw no toxic effects from the shRNA polyplexes. As these results suggest, hydrogels can be developed to deliver combination therapies composed of diverse cargoes without provoking systemic toxicity. And these types of multidrug systems have become an intense area of research in the biomaterials community. These studies considerably amplify the complexity of preclinical studies, but it is essential that future studies directly compare hydrogel delivery against bolus administrations to determine to what extent benefits in safety and efficacy can be attributed to the inclusion of the hydrogel carrier.

Hydrogel delivery of electrostatically assembled nucleic acid particles locally alters gene expression,^236,237^ presenting opportunities for healing highly localized injuries or tissue damage. Khademhosseini and co-workers used this approach with an injectable methacrylated hydrogel to deliver DNA encoding the pro-angiogenic growth factor VEGF.^238^ The DNA cargo was first electrostatically adsorbed onto PEI-functionalized graphene oxide, which was released slowly from the gel depot to promote cardiac tissue repair after myocardial infarction. This group directly compared the effect of using precomplexed DNA particles versus naked DNA in their hydrogel system and determined that hydrogels delivering particles achieved superior tissue regeneration, namely smaller myocardial scar area and improved heart function, as measured by changes in ejection fraction. The hydrogels used in this study did not provoke an immune response or changes in systemic inflammatory markers, which bodes well for future studies aimed at hydrogel-mediated gene therapy for tissue regeneration. In particular, it will be important to see how future iterations of this technology fare with the delivery of mRNA, which has become the preferred approach for expressing exogenous genes.^239^

**Figure 17:**
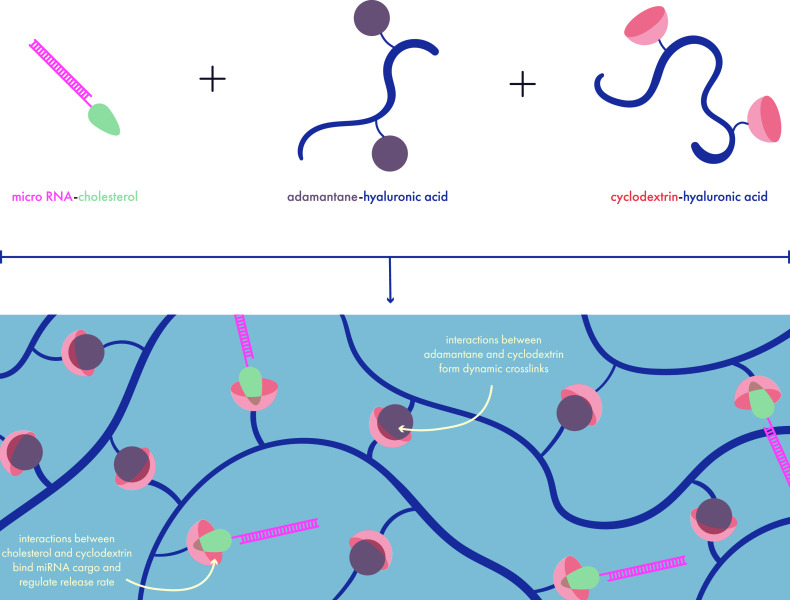
Strategies for sustained release of nucleic acids: engineer affinity interactions between cargo and the network. Nucleic acid cargo can be chemically modified, a routine process to improve its stability and functionality. Chemical modification can also be used to introduce molecular motifs that can engage in supramolecular interactions with partner molecules in a hydrogel network. Researchers used this strategy to deliver microRNA from a supramolecular hyaluronic acid (HA)-based hydrogel. By mixing HA modified with admantane with HA modified with cyclodextrin, a hydrogel forms through the dynamic cross-linking of adamantane and cyclodextrin motifs. Introducing a cholesterol-modified microRNA into this system leads to stable association between the cholesterol and excess cyclodextrins, which allows these hydrogels to slowly release their delicate cargo over the order of weeks. Original illustration inspired from the work of Burdick and co-workers.^246^

A promising alternative to delivering nucleic acid-loaded particles is to use chemically modified nucleic acids (Figure 17).^240,241^ Several phosphatidyl backbone modifications have been identified to improve the stability of delicate RNA and DNA therapeutics, for example.^242,243^ There are also interesting opportunities presented by nucleic acids conjugated to polymers or other biomacromolecules.^244,245^ Burdick and co-workers recently leveraged the capabilities of a cholesterol-conjugated miRNA in an injectable supramolecular hydrogel to locally alter gene expression in heart tissues over the course of several weeks.^246^ By combining two hyaluronic acid derivatives, one modified with pendant cyclodextrins and the other with pendant adamantanes, the authors form a gel from the resulting host–guest network. In addition, the ability for cyclodextrins to form host–guest complexes with cholesterol allows the gel to form high affinity interactions with cholesterol-modified miRNA, which considerably slowed its release rate from the gel. *In vitro*, the release was sustained out to 20 days, and *in vivo*, elevated miR302 could be detected out to 14 days. Remarkably, this approach led to a robust clonal expansion of terminally differentiated cardiomyocytes in a mouse model of myocardial infarction, which corresponded to improved heart function (decreased end-diastolic and end-systolic volumes as well as increased ejection fraction and fractional shortening). One especially exciting aspect of this study is that the use of a confetti mouse model allowed the researchers to provide answers to an open question in cardiac tissue regeneration—whether new cardiomyocytes arise from a progenitor cell type or from pre-existing cardiomyocytes—highlighting how carefully planned biomaterials studies can be used to simultaneously probe unanswered biological questions while advancing clinically relevant technologies.

**Figure 18:**
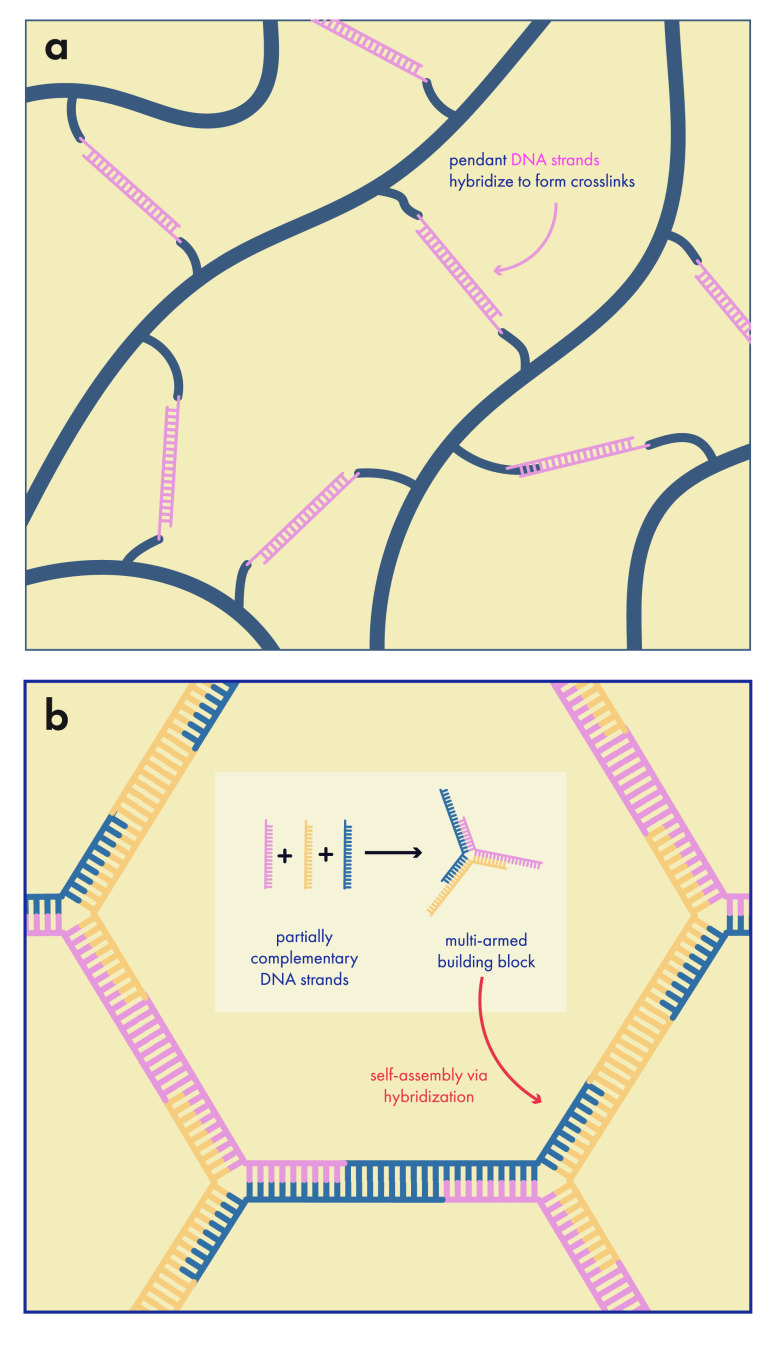
Strategies for sustained release of nucleic acids: make the nucleic acids structural components of the hydrogel itself. The ability for a strand of nucleic acid to hybridize with its complementary strand provides opportunities to develop novel hydrogels based partially or wholly on DNA or RNA. (a) Nucleic acids can be used to cross-link other polymers by attaching specific, complementary sequences along synthetic or natural polymer backbones. Thanks to the commercial availability of nucleic acids with chemically reactive 5′ or 3′ functional groups, a variety of chemical strategies exist to install these macromolecules as pendant chains on polymers. Original illustration inspired from the work of Nagahara and Matsuda.^253^ In addition to cross-linking, these pendent groups can also act as affinity ligands for unmodified therapeutic nucleic acids, such as antisense oligonucleotides or CpG-modified DNA. (b) Alternatively, the hydrogel network can be entirely composed of nucleic acids. Partially complementary oligonucleotides can self-assemble into multiarmed nanostructures. These multiarmed building blocks can be engineered to have single-strand overhangs, or “sticky ends”, that allow them to self-assemble into a highly tunable network. This strategy is particularly useful for delivering immunogenic CpG-modified DNA over sustained periods of time. Original illustration inspired from the works of Nishikawa, Takakura, and co-workers.^250,254^ For both systems, erosion can occur through enzymatic degradation of the nucleic acid cross-links which can simultaneously release entrapped cargo.

Incorporating nucleic acids as cargo is not the only strategy to deliver or use nucleic acids in hydrogels. Extensive work has established a field of DNA-based hydrogels, where the macromolecular network is either partially or entirely formed from nucleic acids (Figure 18).^247,248^ Initial reports of these systems used enzymatic processes to ligate complementary strands of DNA, creating systems similar to the covalently cross-linked synthetic polymer hydrogels.^249^ More recently, supramolecular approaches have taken center stage with these materials, leading to thixotropic hydrogels suitable for creating injectable drug depots *in vivo*.^250,251^ Many DNA hydrogels have been used to deliver immunogenic nucleic acids for applications in immunotherapy, which will be discussed in greater detail in later sections. Hybrid synthetic polymer–DNA hydrogels are recruiting the considerable functionality of engineering nucleic acids into a versatile biomaterials platform. For example, Collins and co-workers demonstrated the coupling of CRISPR-responsive elements into hydrogels provided novel functionalities that included triggered drug release, cell release, and biosensing.^252^ It is particularly interesting that these hydrogels could be used to develop highly sensitive detectors of viral ssRNA, in this case for the Ebola virus. Thanks to the catalytic nature of the Cas12a system used in this study, the virus-sensing hydrogels were able to detect RNA concentrations as low as 11 aM. Considering the capabilities of nucleic acid-based nanotechnologies, we expect that DNA- or RNA-based hydrogels will continue to provide exciting new functionalities to this space. Future work will need to evaluate how stable functional nucleic acid elements are under physiological conditions, as the delicate nature and short half-lives of many of these constructs may be at odds with the prolonged time frames targeted with biomedical hydrogels. Additionally, the extent to which hydrogels can target transfection to a particular cell type is an open question.

### 3.4 Considerations for Protein Delivery

Therapeutic proteins and peptides comprise a major portion of the biopharmaceuticals industry^255^ and have demonstrated extensive value for treating a number of conditions ranging from diabetes, cancer, infectious disease, and arthritis. In general, the stability of protein drugs has been an obstacle, particularly for storing and transporting these drugs, leading to the highly expensive cold-chain transport system that makes these drugs difficult to supply to the rest of the world.^102,256,257^ Often, protein drugs need to be administered repeatedly to maintain their benefit, with treatment frequencies ranging from monthly dosing for certain antibodies to daily dosing for peptide hormones such as insulin.

Hydrogel carriers may provide useful innovations for stabilizing protein drugs during shipment and storage,^258^ as well as minimizing treatment frequency by providing long-lasting sustained release of proteins after they are administered. Typically, proteins larger than 100 kDa are large enough to design hydrogels to regulate their release primarily though gel degradation and diffusion and are reasonably successful in providing a sustained release of the cargo.^259,260^ This approach is promising for important classes of therapeutic proteins, such as antibodies, bulky enzymes, and engineered proteins. For example, Yang and co-workers observed that a physically cross-linked injectable hydrogel was able to deliver a high dose of Avastin, a clinically approved antibody that antagonizes the aberrant angiogenesis in tumors.^261^ Compared to a control treatment regimen, which followed a weekly bolus administration out to 4 weeks, the single hydrogel injection achieved similar efficacy in the HCT116 murine model of metastatic colon cancer. Notably, the hydrogels had a strong impact on the pharmacokinetics of Avastin, reducing the *C*_max_ in circulation ca. 4-fold.

Passive release strategies to focus drug exposure in target tissues could have benefits for a variety of protein therapies with problematic off-target effects. However, for smaller protein drugs (e.g., hormones, peptides, growth factors, or cytokines), passive release runs into many of the same issues facing small molecules, such as burst release and short release windows. So, for smaller cargo and for applications looking at very prolonged release windows, alternative approaches are required to adequately control protein release. Increasing the effective size of the protein cargo can be complicated due to their highly diverse structures and compositions, and proteins are generally not as easy to complex or load into larger particulate systems as, say, nucleic acids. Nevertheless, clever strategies have emerged to generate slow-release systems from injectable hydrogels.

As in prior sections, one approach to slow the release of proteins is to increase their effective size through direct modification (e.g., PEGylation) or through encapsulation into a particle system. Liposomal encapsulation of proteins and subsequent loading into a hydrogel can significantly slow down cargo release. This also creates an opportunity to tune release kinetics and program multidrug release from hydrogels. For example, Hartgerink and co-workers developed a peptide nanofiber hydrogel that encapsulated liposomes within the matrix.^262^ Growth factors could be loaded in the bulk aqueous phase as well as in the liposomal compartment, and in general the protein in the bulk phase was released before the proteins entrapped within the liposomes. Similar particle encapsulation approaches include loading proteins into PLGA,^263^ mesoporous silica,^264^ and calcium carbonate (CaCO_3_) particle systems.^265^ Leveraging nanoencapsulation techniques for protein delivery in this way has clear advantages, but the translational potential of these technologies remains limited by the difficulty of developing scalable and generalizable encapsulation techniques. This is further complicated by the relatively low encapsulation efficiency of proteins compared to other classes of drug.^266^

An alternative approach to increasing the effective size of protein cargo is to introduce interactions between cargo and the hydrogel matrix. This could be done chemically through covalent attachment of proteins to the hydrogel matrix as described in prior sections,^204−207^ but this runs the risk of impacting the bioactivity of the cargo. In contrast, engineering in noncovalent interactions (e.g., hydrophobic, hydrogen-bond, and electrostatic) between proteins and the hydrogel matrix has the potential to greatly slow protein release kinetics without modifying the cargo. Electrostatic interactions have been especially useful to tune the release rates of proteins that carry sufficient net charge under physiological conditions.^267^ For example, this approach appears to be helpful for regulating the release of smaller cationic enzymes, such as lysozyme (Mw ∼ 15 kDa), from injectable hyaluronic acid hydrogels.^267,268^

**Figure 19:**
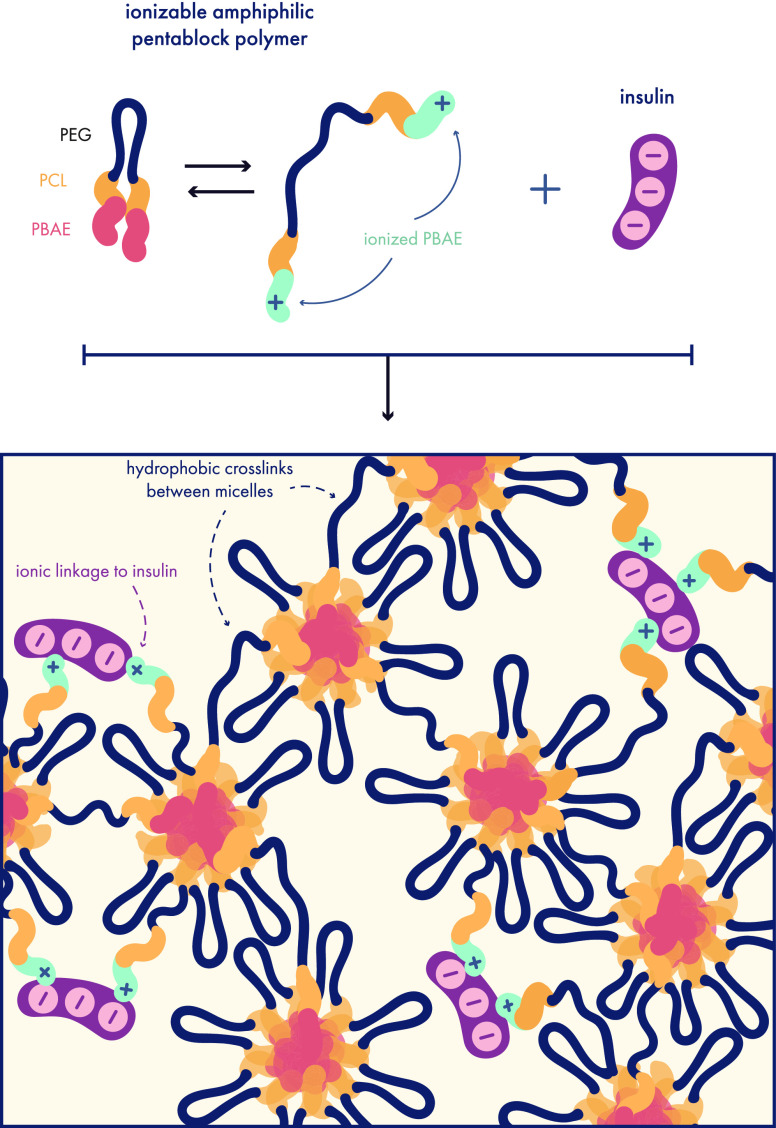
Strategies for sustained delivery of proteins: electrostatic interactions between cargo and network. Some protein cargo exhibit a net charge, which can be taken advantage of to slow their release from a hydrogel carrier. Researchers developed a temperature-sensitive ionizable pentablock polymer that self-assembled into micellar structures. Under physiological conditions, these micelles cross-linked to form a hydrogel but also maintained their electrostatic interactions with anionic insulin cargo. As a result, these hydrogels sustained the *in vitro* release of insulin for over a month. Original illustration inspired from the work of Lee and co-workers.^269^

Electrostatics-mediated affinity seems to be useful for delivering smaller peptide hormones as well, as Lee and co-workers demonstrated with a cationic hydrogel to prolong the release of insulin (Mw ∼ 5.8 kDa), which is net anionic (Figure 19).^269^ As with other hydrogel platforms that directly engage their cargo, these systems also observed that inclusion of the protein drug could alter certain mechanical properties, including gelation behavior. Electrostatic affinity was sufficient to extend the release of insulin out to ca. 35 days *in vitro*, compared to a 5-day delivery window for an analogous, but charge-neutral, hydrogel. *In vivo*, the cationic hydrogel eliminated burst release and maintained steady insulin levels in the blood of rats for ca. 20 days. In contrast, the neutral hydrogel showed a considerable burst release characterized by a spike in blood insulin, which disappeared by 24 h. Yet, even the neutral hydrogel was beneficial compared to bolus administration of insulin, which led to a short-lived (<5 h) spike of insulin in the blood.

While leveraging electrostatic interactions is promising, it does require accessible, charged residues on the protein cargo, and it is unclear just how many charged residues are needed to sufficiently slow down cargo diffusion. For proteins that are insufficiently charged, there are options to attach charged motifs through protein chemistry or protein engineering, but this poses a risk of altering the bioactivity of the protein. At the very least, it introduces additional manufacturing steps that will eventually introduce logistical challenges during scale up of production. Nevertheless, cleverly taking advantage of electrostatic interactions between proteins and the hydrogel offers a straightforward approach to extending release for a select class of cargoes that are small and intrinsically charged under physiological conditions.

The extracellular matrix intrinsically regulates the diffusion of a host of signaling proteins through noncovalent interactions between those proteins and the ECM.^270^ Taking this cue from nature, extensive research into ECM-mimetic hydrogels has led advances in controlled protein delivery.^271,272^ Hydrogels made with biopolymers derived from the ECM have the innate capability to bind to a variety of proteins including soluble growth factors and cell surface receptors. This intrinsic affinity can be leveraged to extend the release of a number of naturally occurring proteins (e.g., fibroblast growth factors, neurotrophins, and bone morphogenic proteins) that are useful for tissue regeneration applications.

Some of the best studied biopolymers for ECM-mimicry have been heparin and heparan sulfate, which are anionic linear polysaccharides that are natural constituents of the ECM.^273^ Heparin exists in diverse states in the body and carries out similarly diverse roles that include growth-factor signaling, chemokine signaling, cellular adhesion, and coagulation.^274^ While ionic interactions are a major contributor to heparin–protein interactions, there are also important contributions from hydrogen bonding and hydrophobic interactions, leading to *K*_d_ values as low as 10^–9^ M.^273,274^ Heparin’s interaction with growth factors, such as bone morphogenic protein-2 (BMP-2),^275^ fibroblast growth factor,^276^ and vascular endothelial growth factor (VEGF),^277^ among others,^202^ has been the basis for several sustained release platforms for tissue regeneration.

**Figure 20:**
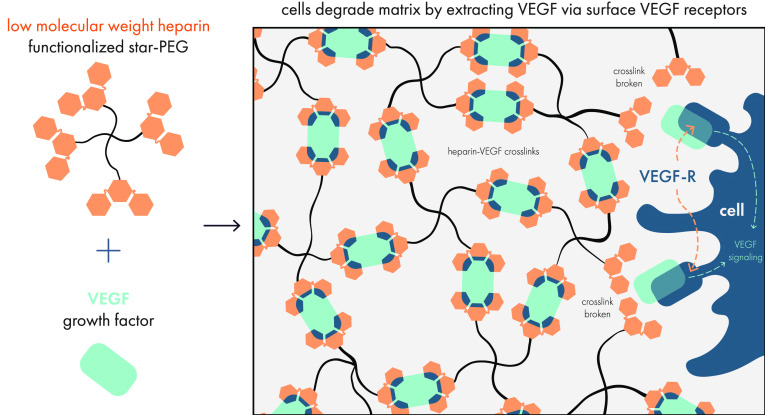
Strategies for stimuli-responsive release of proteins: endogenous cell activity drives both degradation and protein release. Biopolymers such as heparin possess intrinsic growth-factor binding capabilities that can be leveraged to control release of those factors. Taken a step further, engineered heparin derivatives can be used to create innovative dynamic hydrogel formulations with stimuli-responsive behaviors. Researchers functionalized multiarmed star PEG polymers with low-molecular weight (LMW) heparin. When combined with the growth factor VEGF, the LMW heparin ends of the star polymers intrinsically bind the growth factor, giving rise to a dynamically cross-linked network. Cells expressing the VEGF receptor (VEGF-R) can pry the growth factor out of the network, breaking cross-links and slowly degrading the hydrogel. Original illustration inspired from the work of Kiick and co-workers.^51,280^

There have been several strategies to incorporate heparin into synthetic hydrogel platforms, with the development of modified heparin derivatives providing a good deal of design flexibility. Heparin can be readily modified through its carboxylic acid groups without compromising the prominent anionic characteristics from its abundant sulfate groups. Heparin derivatives include cross-linkable heparin and cross-linkable hybrid polymers with useful properties that include *in situ* gelation and injectability.^278,279^ One especially innovative approach for an injectable and stimuli-responsive heparin hydrogel was developed by Kiick and co-workers, which functionalized a star PEG polymer with low-molecular weight heparin molecules (Figure 20).^280^ The resulting multiarmed building block could be cross-linked into a hydrogel by the addition of free VEGF, thereby directly incorporating the therapeutic cargo as a structural component of the hydrogel itself. Erosion of the gel could then be triggered by a ligand-exchange mechanism; that is, when cells expressing the receptor for VEGF (VEGFR) came in contact with the gel, they could harvest the VEGF cross-links to slowly degrade the hydrogel.^51^ In addition to heparin/heparan sulfate, other constituents of the ECM have demonstrated specific interactions with growth factors and other soluble signaling proteins, including collagen,^281^ fibronectin,^282^ and vitronectin,^283^ among others. These biopolymers provide a broad armamentarium for developing ECM-mimetic hydrogels capable of delivering specific growth factors.^284^ Nevertheless, it is worth pointing out that these materials are highly multifunctional, with many promiscuous binding sites capable of engaging diverse binding partners. As a result, it may be difficult to fully anticipate how the release behaviors of these systems will respond to the presence of endogenous factors after implementation or if these biopolymers will mediate unanticipated functions beyond regulating cargo release.

**Figure 21:**
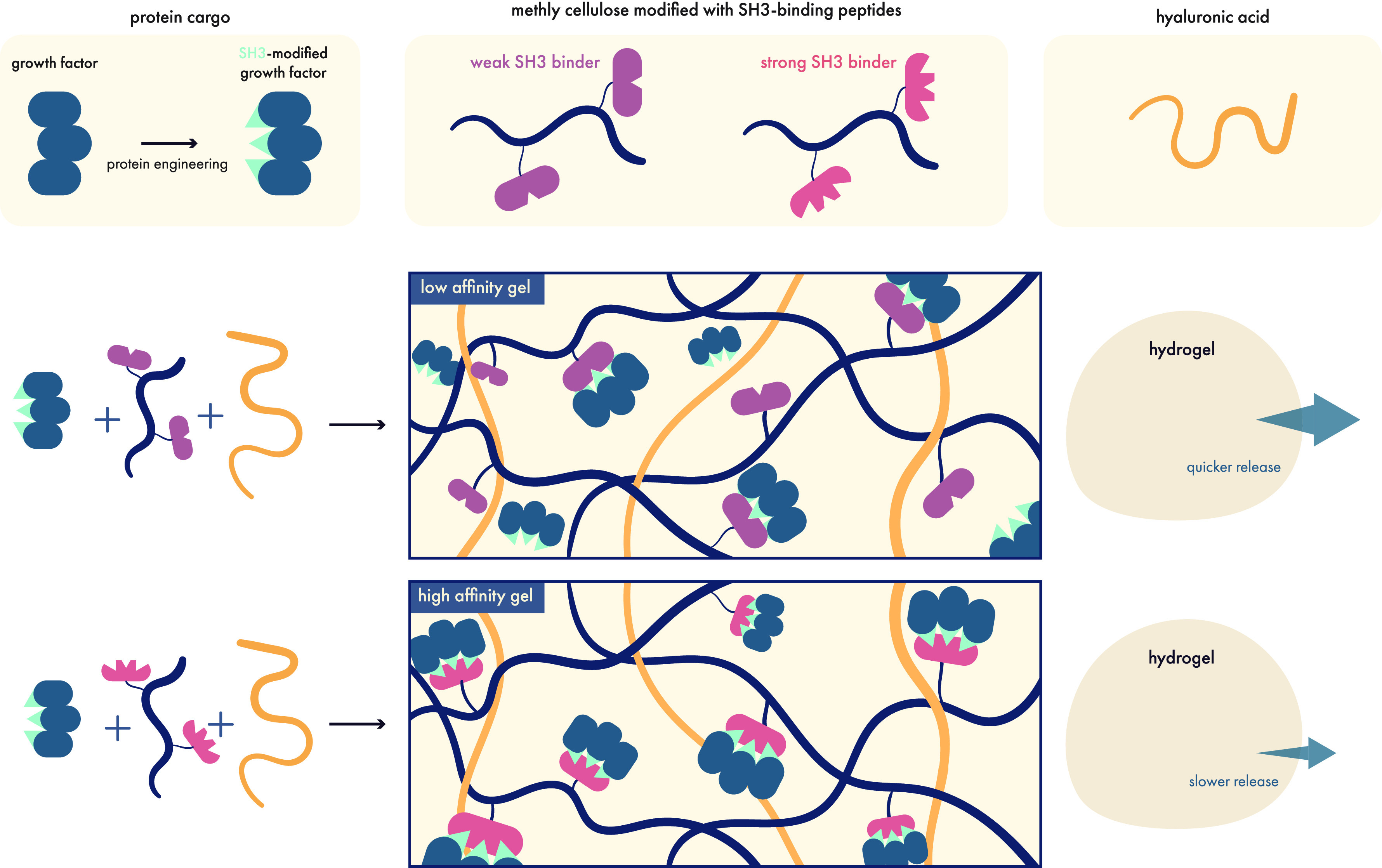
Strategies for sustained delivery of proteins: engineering affinity interactions between proteins and hydrogels. Macromolecules with specific affinity for therapeutic cargo (e.g., peptides, antibodies, or aptamers) can be chemically introduced into hydrogel networks to yield affinity release systems. Researchers exploring the capabilities of affinity release systems have shown that by incorporating low- or high-affinity motifs, the release rate of cargo can be modulated. Likewise, varying the stoichiometric ratio of binding motifs and cargo can further fine-tune release. Original illustration inspired from the work of Shoichet and co-workers.^286^

More sophisticated methods to govern the release of proteins from hydrogels are being developed that introduce affinity interactions between the hydrogel and cargo. These approaches rely on highly specific supramolecular interactions between the cargo and the hydrogel matrix that can include host–guest and ligand–receptor interactions.^285^ This often takes the form of engineering cargo (via protein engineering or chemical modification) to exhibit a binding domain that can be specifically recognized by another molecular motif attached to the hydrogel matrix. For example, Shoichet and co-workers developed an injectable, peptide-modified, polysaccharide-based hydrogel to control the release kinetics of fibroblast growth factor (FGF).^286^ In this work, FGF was fused to a Src homology 3 (SH3) domain which introduced a “handle” for a supramolecular interaction with SH3-binding peptides conjugated along the hydrogel network (Figure 21). By using low-affinity or high-affinity SH3-binding peptides, the authors were able to tune FGF release kinetics, and in subsequent work they have developed a mathematical model to elucidate key parameters in programming a specific drug release profile in these systems.^287^

**Figure 22:**
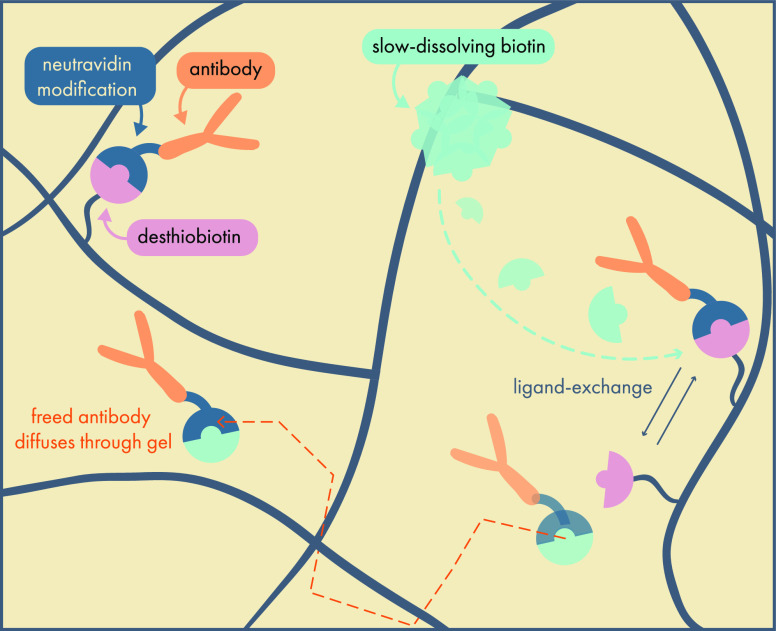
Strategies for tuning affinity release of proteins: coencapsulation of competitive ligands. Researchers engineered a desthiobiotin-conjugated hydrogel for the sustained release of neutravidin-modified antibodies. To tune release kinetics, a slow-dissolving biotin derivative could be coencapsulated to introduce free biotin ligands over time. As the amount of free biotin ligands increased in the gels, they drove ligand exchange to liberate antibodies from the hydrogel network, allowing them to diffuse through the hydrogel. The rate of release could ultimately be tuned by the total amount of slow-dissolving biotin that was coencapsulated in the hydrogels. Original illustration inspired from the work of Wylie and co-workers.^288^

In a similar approach, Huynh and Wylie used poorly soluble biotin derivatives to tune the release rate of desthiobiotin-modified antibodies from a neutravidin-modified injectable hydrogel (Figure 22).^288^ Without the slow-dissolving biotin derivative, these hydrogels release antibodies extremely slowly (∼5 ng per day) due to the high-affinity interaction between desthiobiotinylated antibodies and neutravidin. To disrupt that interaction, a poorly soluble derivative of biotin could be coencapsulated into the hydrogels. As this biotin derivative slowly dissolves over time, it introduces free biotin to compete for the neutravidin binding sites within the gel. This strategy leads to a ligand-exchange-based release mechanism that can be tuned by the amount of biotin derivative coencapsulated within the gel.

These types of approaches provide impressive control over protein release, but they also require modification of the protein cargo with molecular “handles” that can interact with binding motifs tethered to the hydrogel. This can be challenging for certain proteins or have unintended consequences on bioactivity, and from a translational point of view, it introduces additional processing steps that complicate fabrication. An alternative to this is to use binding motifs that recognize the native protein cargo. Apart from antibodies, this type of specific interaction has been hard to incorporate into biomaterials until somewhat recently. And while high-quality antibodies exist for numerous therapeutic proteins, their high target affinity (which is not easily tuned) can lead to extremely slow release. In these systems, release is generally strongly erosion-dependent and can limit certain applications. That being said, Zhao et al. reported that anti-BMP-2 antibody-modified collagen gels could improve bone regeneration *in vivo*, potentially due to extended release of coencapsulated BMP-2.^289^ Nevertheless, alternative mechanisms, such as antibody-gel mediating a locally elevated concentration of endogenous BMP-2, cannot be discounted based on this study, especially in light of reports that other growth-factor binding hydrogels can mediate tissue regeneration in the absence of exogenous growth factor.^290^ Delivery applications aside, antibody-based hydrogels are also interesting from a stimuli-responsive perspective, such as antigen-induced swelling materials.^291^

More recently, directed evolution techniques have unlocked the ability to generate novel binding motifs such as aptamers and peptides toward diverse targets.^202^ These new ligands are especially promising for hydrogel carriers thanks to their rapid development (relative to antibodies), small size, ease of synthesis, and options for site-specific bioconjugation. As a brief summary, directed evolution uses the principle of natural selection to develop novel proteins, peptides, or nucleic acids able to carry out user-defined catalytic or binding functions. This process generally involves the introduction of random mutations in a precursor biomolecule to generate a library that can be screened for improved functionality. In terms of screening techniques, numerous options are available for assessing binding capabilities, including displaying candidates on phage, bacteria, and yeast. The highest performing mutants are then selected for amplification to use as the template for subsequent rounds of mutations and selection. Overall, this approach has been instrumental in the development of new proteins (particularly antibodies) and peptides with high affinity toward specific targets. Going beyond protein engineering, the directed evolution technique known as systematic evolution of ligands by exponential enrichment (SELEX) has led to the development of new nucleic acid-based targeting moieties, known as aptamers, that can provide target affinities comparable to antibodies. Importantly, these approaches allow researchers to develop ligands for a specific target and also provides them with a library of candidates ranging from low to high affinity for that target. By incorporating novel, cargo-specific ligands into hydrogels or other biomaterials, excellent and specific control of cargo release is possible, without the need to modify cargo in any way.

Recent work leveraging aptamers in hydrogels indicates the promise for engineered ligands for protein delivery. Wang and co-workers developed aptamer-functionalized polyacrylamide gels to sustain the release of antiplatelet derived growth factor-BB (PDGF-BB).^292^ In this system, anti-PDGF aptamer was modified at its 5′ terminus with an acrylamide functional group, which allowed the aptamer to be directly incorporated into the hydrogel during the free radical polymerization of acrylamide. Notably, release rates of the growth factor could be tuned by using low- or high-affinity versions of the aptamer. In a follow-up study, this group demonstrated that the aptamer affinity approach allowed for two release modalities—an extended slow release (governed by the aptamer binding kinetics) and a rapid triggered release (governed by introduction of complementary oligonucleotides). Introduction of complementary oligos outcompetes the aptamer–target interaction, which can drive a ligand-exchange mechanism.^293^ Notably, this approach can be used to independently control the delivery of multiple protein drugs from the same hydrogel.^52,294^

Overall, affinity hydrogel approaches may form the basis for highly programmable protein drug release, which will be essential for directing multidrug delivery aimed at shaping complex biological outcomes such as tissue regeneration. These exciting improvements in delivering protein directly should also be considered alongside alternative routes for protein therapy, such as the delivery of DNA or mRNA that encodes therapeutic proteins, which we discussed in the prior section. In particular, the possible time scales from “direct protein delivery” should be compared to gene delivery platforms. For example, with direct delivery of protein, the therapeutic molecules are immediately available until the hydrogel reservoir is exhausted. In contrast, gene therapies will see a lag before protein is manufactured from the genetic templates, and then the duration of protein expression will depend on a complex mix of factors including the immunogenicity, the half-life of the nucleic acid cargo, and the permanence of any genome modification. As synthetic biology continues to introduce novel constructs such as RNA replicons, the time scales for protein delivery may begin to favor gene delivery approaches for long-term, sustained delivery. That being said, many CRISPR-based approaches rely on the codelivery of protein and guide RNA, which indicates a need for sophisticated carriers of both types of cargo.

### 3.5 Injectable Hydrogels for Cancer Immunotherapy

The prior sections summarized important considerations for the delivery of a variety of therapeutic cargoes. In this section, we use the application of hydrogels for immunotherapy as a case study to continue discussing the delivery of small molecule, protein, and nucleic acid cargoes. The recent successes of cancer immunotherapy have led to an explosion of research in this area, which has led to exciting innovations in drug delivery technology to overcome the challenges associated with delivering multiple, diverse therapeutic cargoes that include small molecule adjuvants, antibodies, and antigen-encoding mRNA, to name a few. Given the unique spatial compartmentalization and variable time scales involved in mounting and manipulating the immune response, immunomodulatory hydrogels are pushing the boundaries on skillful delivery of all three categories of drugs. We expect that these advances will prove impactful on the emerging field of immunoengineering and that the drug delivery concepts developed by these materials will also prove useful for drug delivery applications outside of immunotherapy.

**Figure 23:**
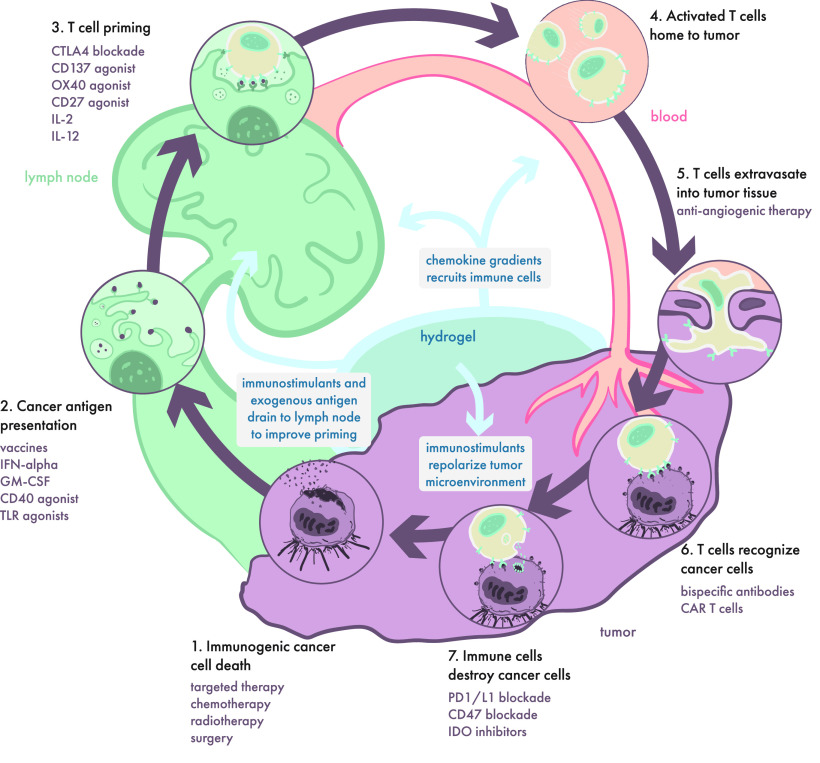
Hydrogels meet the cancer immunity cycle. Cancer immunotherapies follow a cyclic process that can be thought of as beginning with (1) immunogenic cancer cell death. This death releases cancer antigens which can be (2) taken up by antigen presenting cells, which can (3) display them to naïve T lymphocytes in the lymphatic tissues. Activated anticancer T cells then (4) migrate to cancerous tissues via the blood, where they (5) extravasate into tumors and (6) lock onto the specific tumor cells presenting their cognate antigen. After recognizing their target, these T cells can then (7) directly kill the cancer cell, starting the cycle anew. Tumors evolve mechanisms that can combat this cycle at every step, and effective immunotherapy can selectively disable those adaptations. In this figure we include therapeutic strategies for each step which could drive cancer immunity. We illustrate how a hydrogel can directly influence stages of this cycle, in particular through enhancing immune cell recruitment, as well as through local drugging of the tumor and the draining lymph node. Original illustration inspired from the work of Chen et al.^295^

Hydrogels are especially well suited to simultaneously address the weaknesses and bolster the strengths of current immunotherapy strategies (Figure 23). Importantly, in the context of immunotherapy, hydrogel drug delivery becomes a viable approach for addressing metastatic cancer. Previously, the localized therapy that hydrogels provide had limited utility for treating metastasized disease, because even with injectable systems it becomes impractical to locally inject hydrogels near tumors that are widely disseminated across variable organ systems.

So, in the context of metastasis, hydrogel drug delivery strategies were limited to treatments following surgical resection or to depots that could provide sustained elevated drug levels system-wide. The first of these strategies benefited little from the advances in injectable hydrogels, and the second strategy missed out on the potential for hydrogels to minimize side effects and toxicity. The advent of immuno-oncology, however, shifted the paradigm for treating metastatic disease. If a hydrogel could be administered to one tumor, and successfully mount an immune response, the systemic nature of immunity could lead to the clearance and elimination of distant tumors. This capacity to affect distant, untreated malignancies is referred to as the abscopal effect, a term coined for the observation that radiotherapy of one tumor could surprisingly inhibit the growth of untreated tumors—which we now know is attributable to the immunogenic cell death caused by radiation therapy.^296−298^

Using hydrogels for local immunotherapy has tremendous potential, as one of the major barriers for advancing new cancer immunotherapies has been dose-limiting toxicity.^299−301^ By limiting the stimulation of the immune system to the tumor microenvironment, hydrogels are likely to make immunotherapies more tolerable,^302,303^ as has been observed in prior research using chemotherapy drugs. This potential is supported by early studies that aimed to curb the toxicity of immunostimulants, which used simple viscous fluid carriers to locally administer immunotherapeutics and markedly reduce immune-related toxicities.^304−306^ Compared to these viscous fluids, hydrogels are much better regulators of drug release, and we expect that they will offer even greater benefits. Additionally, the ability for hydrogels to serve as a tissue scaffold allows them to be more than just a drug depot; when designed carefully they can actively recruit and host endogenous immune cells to cultivate an immunogenic niche.^307,308^ For cancer, this is a major benefit, as it serves as a direct foil to the immunosuppressive environment within the tumor microenvironment.^309^

The utility for hydrogels in immunotherapy extends beyond cancer, and we expect hydrogel vaccines to become of significant interest in the coming years. This is in part because hydrogels can orchestrate release kinetics that better mimic the dynamics of a natural infection. Several studies indicate that extended release kinetics are especially beneficial for mounting potent and highly desirable humoral responses for diseases such as HIV.^310^ And just as hydrogels can be designed to foster immunogenic niches, they can be engineered to establish immunosuppressive, or tolerogenic niches, to combat autoimmune disease.^308^ This capacity to specifically “train” an immune response is particularly interesting for developing novel immunotherapies, but it may also prove to be useful for studying the immune response through highly tunable immuno-interfaces.

#### 3.5.1 Stimulating Innate Immunity

The innate immune system constantly surveils the body for signs of infection or dysfunction, using numerous toll-like receptors to detect highly conserved macromolecules associated with pathogens or pathogen-associated molecular patterns (PAMPs).^311^ These include the detection of certain lipids, nucleic acids, and proteins that are conserved in microbiol pathogens but not in vertebrates. Triggering these signaling pathways is critical for a sustained immune response, and integration of these signals by antigen presenting cells (APCs) governs critical decisions that ultimately determine the type of immune response generated. Because cancer cells bear significant resemblance to healthy cells, these innate immune signals are often not present during oncogenesis,^309^ but by providing exogenous signals it is possible to reinvigorate pre-existing, but suppressed, immune responses or even generate entirely denovo immune responses to cancerous tissue.^312^

Several PAMPs include nucleic acids and their derivatives, and as discussed previously, there are several strategies to deliver anionic nucleic acids from cationic hydrogels leveraging electrostatic interactions. Hartgerink and co-workers used this approach to deliver a cyclic dinucleotide agonist of the cGAS-STING pathway,^313^ a potent immunostimulatory sensor of cytosolic DNA. In nonimmune cells, activating this pathway can trigger senescence and cell death. In innate immune cells, such as DCs, this pathway leads to maturation, the production of cytokines, and improved T cell priming.^314^ STING agonists have shown significant promise for cancer immunotherapy, but they pose serious toxicity concerns due to their potency.^315^ Controlled delivery of these small dinucleotides is challenging even with local injections, and their ability to rapidly diffuse through tissues and into systemic circulation leads to poor pharmacokinetics and therefore frequent dosing. To address some of these challenges, these researchers used an injectable supramolecular hydrogel composed of multidomain peptides, which self-assemble in the presence of multivalent ions.^313^ By engineering the peptides to bear additional cationic lysine residues, the hydrogels could electrostatically associate with cyclic dinucleotides. *In vitro*, this approach extended release ca. 3-fold (5 to 14 h) compared to neutral collagen hydrogel controls.

While this release window is still relatively short, these gels led to significant improvements in a murine model of head and neck cancer compared to either local injections or collagen control gels. This improvement may derive from the longer release of STING agonist, but the authors also observed robust immune cell infiltration into the multidomain peptide gels that implies that the formation of an immunogenic niche is critical for efficacy. Future studies that disentangle the relative contributions of controlled release from the niche effect will hopefully direct efforts into developing whichever mechanism is more important. Granted, the design of such studies will be challenging since the formation of these niches appears to depend just as much on the identity of the cargo as it does on the identity of the hydrogel.

Immunomodulatory cytokines provide another powerful means to engineer the strength and type of immune response.^316^ This class of secreted proteins govern much of the paracrine and autocrine cellular signaling involved in initiating, maturing, maintaining, and finally resolving immune responses. Because the receptors for cytokines are fairly ubiquitous, most cytokines are captured and “used up” relatively quickly within the body.^317^ This presents a challenge for typical administration routes such as systemic infusion, where the majority of administered exogenous cytokine may be captured by healthy tissues prior to reaching the target tissue. This behavior also contributes to elevated risk for toxicity, particularly notable with the failed clinical translation of highly potent cytokines such as IL-12.^318^ Unsurprisingly, the pharmacokinetics of exogenous cytokines is generally poor and has required clever engineering solutions to extend their half-lives *in vivo* (e.g., the fusing of IL-2 to Fc domains).^319−321^ Hydrogel carriers are able to address these issues by sustaining the release of cytokines within or next to the target tissue. The close proximity of the depot allows high therapeutic concentrations of cytokine over an extended time window to achieve robust changes in the local immune microenvironment. Toward this end, there have been promising studies using injectable hydrogels to deliver IL-2,^322,323^ IFNa,^324^ and IL-12,^325^ which are all critical mediators of a type I immune response. Overall, these studies indicate that hydrogels can extend release of their cargo out to around 1 week using passive approaches, with the ability to tune release somewhat by tuning the stiffness or solids content of the hydrogel carrier. Leveraging electrostatic interactions appeared to further extend that release of protein cargo, generating hydrogels that could sustain release of “model” cargo over 2 weeks *in vitro*, although this was not directly confirmed with the target cytokine.^325^ From these early studies, a major challenge for cytokine delivery with hydrogels may be maintaining the bioactivity of unreleased cargo, as some studies report a precipitous drop in function of encapsulated IL-2 after about 1 week.^323^ Future studies ought to explore next-generation techniques, such as affinity mechanisms and mRNA delivery, to evaluate the benefits of longer term release kinetics. At the same time, it will be important for future work to leverage the multiplexed cytokine profiling technologies, such as Luminex, to better elucidate cytokine dynamics and cross-talk in specific microenvironments (e.g., draining lymph nodes, tumor tissue, and the hydrogel).

An alternative to delivering PAMPs or cytokines is to use tumor-specific antibodies to stimulate antibody-dependent cellular cytotoxicity (ADCC).^326^ This approach takes advantage of the ability for certain Fc domains of antibodies to engage with Fc receptors on certain innate immune cells, such as macrophages. With the right Fc domain, this triggers phagocytosis and destruction of antibody-decorated tumor cells.^327^ It is worth noting that there are numerous Fc domains, and engineering this region of antibodies can lead to variants that can promote or prevent ADCC, among other things.^328^ It is also worth noting that while certain Fc regions may function one way (e.g., promote ADCC) in mice or nonhuman primates, the analogously named Fc domain in humans can have an entirely different function due to interspecies differences in Fc receptors.^329−331^ Certain antibodies can also initiate the complement cascade, a part of the innate immune system which rapidly perforates cell membranes and causes immunogenic cell death.^332^ Overall, ADCC allows innate immunity to attack tumor cells, which can potentially lead to the generation of a *de novo* endogenous adaptive immune response, especially if paired with other immunostimulants.^333^

**Figure 24:**
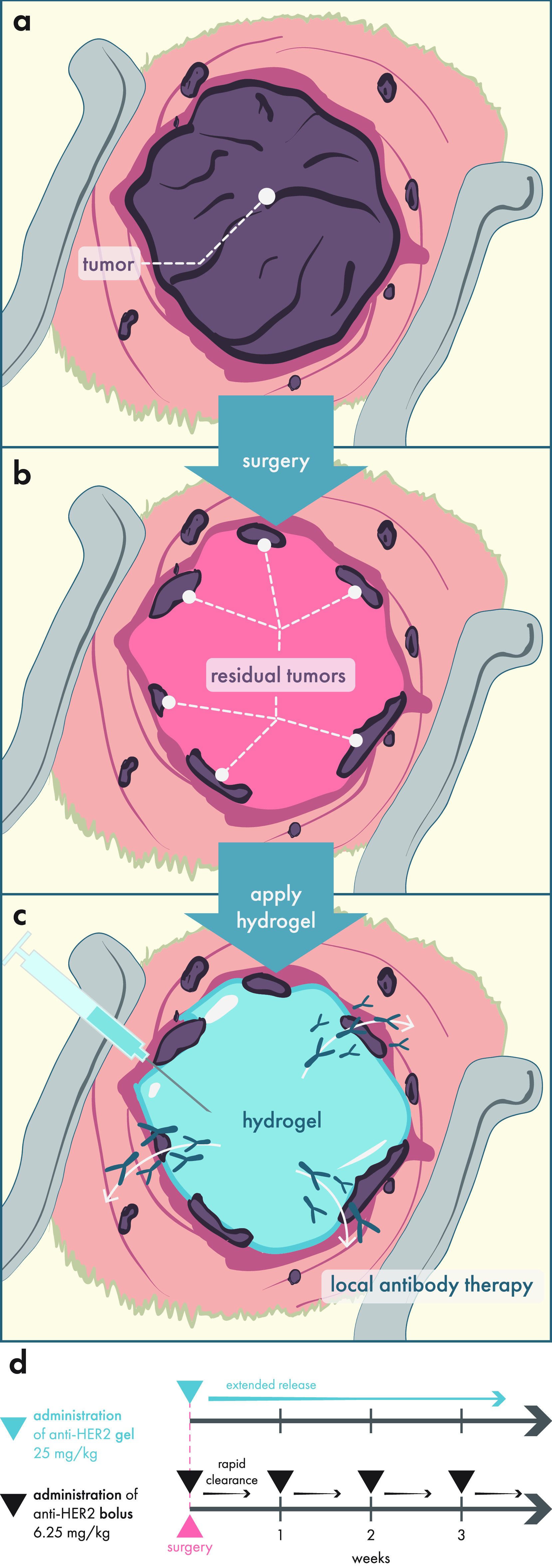
Local, sustained delivery of tumor-targeting antibody prevents relapse in a model of breast cancer. Researchers developed an injectable hydrogel that can be applied to the site of tumor resection surgery. A single application of this hydrogel could contain 4 times the dose used for weekly systemic administration and yet caused fewer toxic side effects. In addition to the improved safety profile, the HER-2 hydrogels were more effective at preventing tumor recurrence. Original illustration inspired by the work of Ding and co-workers.^334^

Several studies have shown the utility of hydrogel depots of tumor-targeting antibodies. For example, Ding and co-workers used a temperature-sensitive injectable hydrogel to deliver anti-HER2 monoclonal antibodies (Herceptin) to the surgical cavity following primary tumor resection in a murine model of HER2+ breast cancer (Figure 24).^334^ For this type of cancer, Herceptin forms the backbone of nearly all frontline therapies and is therefore essential for treating these patients.^335^ Unfortunately, the wide expression of HER2 on healthy cells leads to toxic side effects, in particular, cardiotoxicity which can range from subclinical to fatal cardiac failure, especially when treatment is combined with chemotherapy.^336,337^ By injecting Herceptin-loaded hydrogel into the surgical cavity after resection, high local concentrations of Herceptin were achieved which appeared to prevent tumor recurrence. Critically, the hydrogels kept the antibody from significantly spilling into systemic circulation, which eliminated the cardiotoxicity observed with the control treatment—4 weekly intravenous antibody administrations. Notably, a single hydrogel injection could deliver the total amount of antibody that was spread across the 4 weekly infusions yet still prevented cardiotoxicity.

In addition to improved safety, the hydrogel formulation had better overall efficacy, likely due to maintaining high Herceptin levels within the resection cavity. These results are consistent with a prior study by Yang and co-workers that used an injectable hydrogel carrier of Herceptin to treat a primary breast tumor in the murine BT474 model.^338^ Namely, these authors also observed improved efficacy with hydrogels compared to dose-matched bolus injections, despite using a system with a significantly faster release rate (5-days to 50% *in vitro* release versus ca. 25 days for the temperature-sensitive system). From this, it appears that any depot effect is beneficial, but more work will be needed to determine the extent to which release rate ultimately impacts efficacy and safety—preferably though direct comparisons within a single study. In both of these studies, use of a human cancer xenograft necessitated using immunocompromised murine hosts, which provides an incomplete picture of the broader immune response for this type of therapy. Future studies that evaluate tumor-targeting antibodies within immuno-competent hosts will be useful for determining how local tumor-targeted antibody therapies might synergize with immunotherapeutics such as checkpoint inhibitors.

#### 3.5.2 Localized Combination Immunotherapy

An important frontier for immunotherapy is the development of safe combination therapies. This is particularly critical in immuno-oncology, where a subset of patients responds remarkably well (some patients, such as former President Jimmy Carter, have seen complete remission of even metastatic disease), but the majority of patients do not benefit from current approaches. One theory is that multiple redundant immunosuppressive pathways must be targeted in the tumor and that combination approaches could sufficiently disable these layered defenses to illicit a potent antitumor immune response.^339,340^ Combinatorial immunotherapy is challenging in several ways, with the most notable perhaps being significant increases in toxicity seen in clinical trials.^341^ Combining immunomodulating agents is also complicated by schedule-dependent effects that are only beginning to be understood but that have a clear and significant effect on both safety and efficacy.^342−344^ Initial studies into combination immunotherapy are also revealing that dosing strategies might not need to resemble the conventional approaches established by chemotherapy, such as multiple cycles of drug. For example, clinical trials that combined PD-1 and CTLA-4 checkpoint antibodies reported that patients who ultimately discontinued the trial due to toxicity saw similar benefits to patients who completed the trial.^345,346^ These data provide a provocative basis to explore different dosing frequencies and strategies.

Hydrogel delivery of combination immunotherapy is local and may avoid stimulating distant lymphatic organs, which could reduce the frequency or strength of side effects such as cytokine release syndrome^347^ or spare sensitive organ systems, such as the gut, that must maintain an exquisite balance of stimulatory and inhibitory signals to maintain peaceful coexistence with the microbiome.^348^ The use of injectable systems is also able to facilitate minimally invasive treatments, which ideally would leave a resorbable depot to treat the area for days or weeks after a single injection. As these depots degrade, they can serve as scaffolds for immune cells to create an immunogenic niche to further support the immune response.^349^ Next-generation hydrogels are being developed that can release different drugs at specific times or after specific cues,^350^ which offers controlled scheduling of drugs in a local context. Overall, this is a research area with great promise and initial studies have revealed compelling data.

Early studies combined immunotherapy with chemotherapy, a strategy based on the ability of certain chemotherapy drugs to induce immunogenic cell death—that is cell death that leads to an immune response.^351^ It is not entirely clear which chemotherapy drugs can induce immunogenic cell death based on their pharmacological mechanisms alone, but this can be determined empirically as was demonstrated by Son and co-workers, who used an injectable chitosan hydrogel to test the combination of different chemo drugs (doxorubicin, cisplatin, or cyclophosphamide) with the inflammatory GMCSF cytokine.^352^ They found that cyclophosphamide synergized best with GMCSF and that inclusion of GMCSF was important for durable anticancer responses in the TC1 murine model of cervical cancer. This study used an intratumoral injection, which appeared to foster an increase in CD8+ killer T cells within the tumor. None of the combination regimens induced weight loss in the mice, suggesting the treatments were relatively safe—although it is difficult to be certain without additional data on toxicity biomarkers or histology. Similar results have been reported for hydrogels delivering doxorubicin, camptothecin, and cisplatin with diverse cytokines.^353−356^

Therapies combining different, specific immunomodulators are the next frontier for local therapy, which can entail delivery of compounds with considerable physicochemical differences. While this can complicate traditional infusion, recent studies are highlighting the ability of hydrogel carriers to simultaneously deliver versatile classes of immunotherapy drugs. Irvine and co-workers demonstrated this capability with an injectable alginate system loaded with calcium-containing microspheres.^357^ The mechanical properties of the gel were tunable by the calcium content of the microspheres, and the cytokine IL-2 could be loaded within the aqueous phase of the hydrogel where it was slowly released by passive diffusion. Meanwhile, short nucleic acid fragments of CpG, a well-characterized and potent stimulator of APCs,^358,359^ could be loaded through electrostatic adsorption onto the calcium-containing microspheres. This combination led to cellular infiltration of the hydrogels *in vivo* and sustained the release of both drugs *in vitro* for about a week.

**Figure 25:**
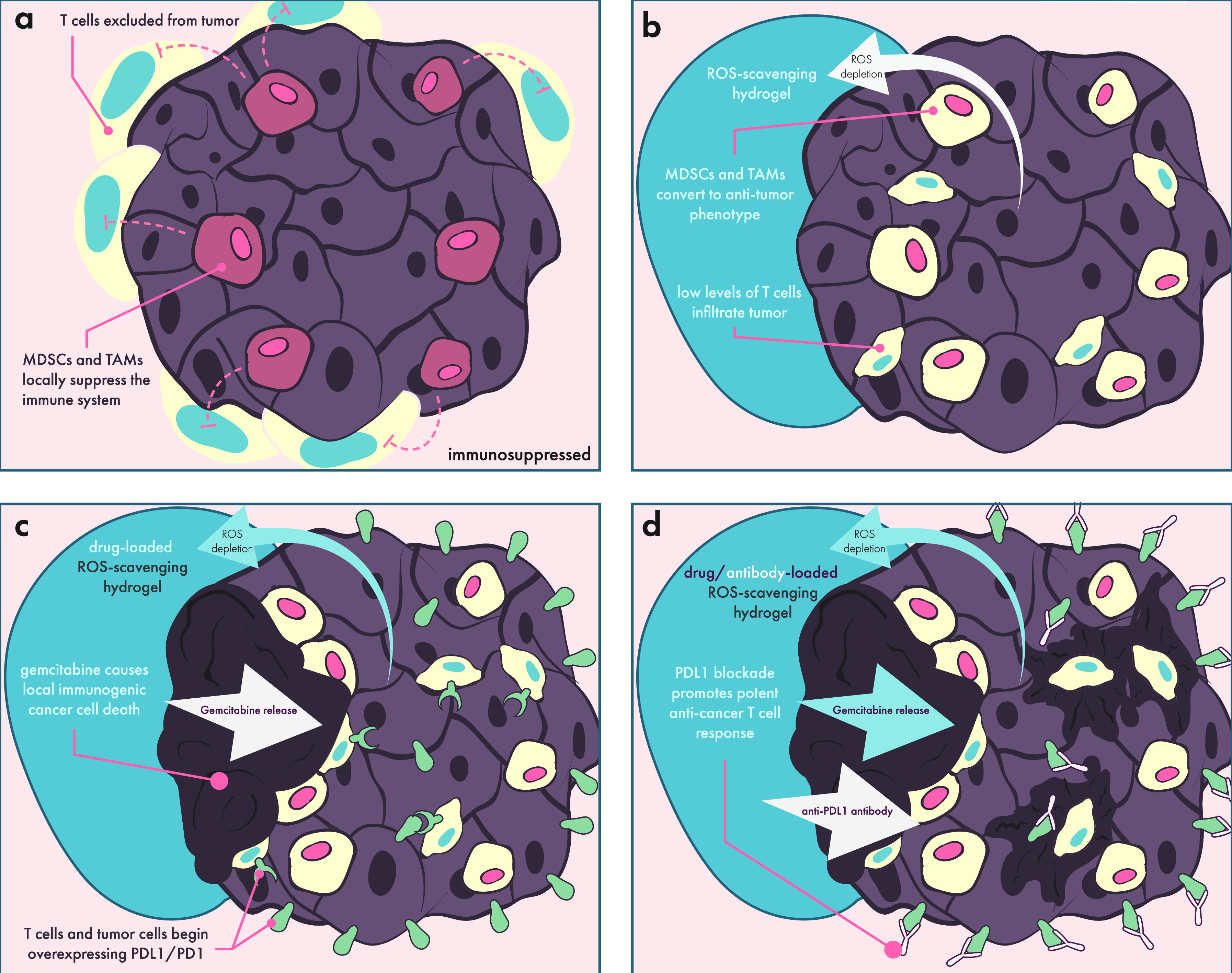
Layered strategies to overcome redundant immunosuppressive mechanisms. (a) In a typical immune-desert or “cold” tumor, T cells are excluded from the bulk of the tumor. Meanwhile, pro-tumor myeloid cells such as myeloid-derived suppressor cells (MDSCs) or tumor-associated macrophages (TAMs) create a tolerogenic microenvironment within the tumor. (b) Researchers developed a hydrogel that scavenges reactive oxygen species (ROS) as it degrades. By depleting the tumor microenvironment of the ROS, the protumor myeloid compartment is repolarized to an antitumor phenotype and facilitates tumor penetration of T cells. (c) Release of the chemotherapeutic gemcitabine causes local immunogenic cancer cell death, driving an immune response and further increasing killer T cell infiltration. However, this immune response triggers expression of PD-1 and PD-L1 checkpoint proteins on T cells and cancer cells, respectively. (d) Inclusion of PD-L1 antibody into the hydrogel disables the checkpoint defense mechanism and drives complete tumor eradication. Original illustration inspired by the work of Gu and co-workers.^361^

More recently, Gu and co-workers have been developing ROS-reactive hydrogels for combination immunotherapy (Figure 25).^265,360,361^ Central to this platform is the use of the hydrogel scaffold itself as a therapeutic element. By reacting with, and therefore scavenging, ROS, this hydrogel has the ability to repolarize the inflammatory state of the tumor microenvironment—where high levels of ROS are thought to promote protumor myeloid cell phenotypes.^362^ In theory, this approach may be helpful for treating “cold” tumors, or tumors that are overall poorly immunogenic.^363^ In an initial study, Gu and co-workers evaluate this platform using the chemotherapy drug gemcitabine, which has well documented immunogenic properties.^361^ In the B16F10 and 4T1 models of cancer, local delivery of gemcitabine using the ROS-scavenging hydrogel suppressed local immunosuppressive cells (MDSCs and TAMs) while increasing intratumoral CD8+ and CD4+ T cells. These shifts in immune cell numbers corresponded to an increase in type 1-associated systemic cytokine levels, consistent with the activation of an immune response. Importantly, flow cytometry analysis also revealed that tumor cells and T cells had increased expression of PD-L1 and PD-1, respectively, strongly indicating that inclusion of a checkpoint inhibitor would further improve the immune response. Physical encapsulation of PD-L1 antibody (aPD-L1) within the hydrogel led to a significant improvement in overall survival, which correlated with more abundant tumor infiltrating lymphocytes than were seen with the gemcitabine gels. One interesting outcome for this therapy is that due to the size difference between gemcitabine and aPD-L1, the chemotherapy drug is released substantially quicker than the antibody. This order of release would in theory better support the natural progression of immunity, namely where immunogenic cell death is later followed by a T cell response, but future studies will need to evaluate to what extent the difference in kinetics matters in this therapy.

In a companion study, Gu and co-workers used their ROS-reactive hydrogel to codeliver a PD-L1 and D1MT, a small molecule inhibitor of indoleamine-pyrrole 2,3-dioxygenase (IDO).^360^ IDO is a secreted enzyme that exerts strong immunosuppressive effects on T cells and is overexpressed in many tumors. By incorporating D1MT directly into the ROS-sensitive polymer backbone of the hydrogel, this platform achieved very slow release of the inhibitor as gels eroded in ROS-rich environments. This combination therapy led to improved outcomes in the B16F10 model, which corresponded to more TILs and less ROS within the TME. Importantly, the hydrogel version of the therapy appeared to be safer than the free drug version, which was associated with histopathologic irregulates. In addition to this apparent safety benefit, the efficacy of the treatment was significantly improved when using the hydrogel carrier. Whether this improved efficacy is due to the unique ROS-scavenging capabilities of the gel or to the sustained release kinetics remains an open question.

Gu and co-workers later applied their ROS-reactive hydrogel platform to target a significant barrier in immuno-oncology, namely mounting an immune response against cancers with low levels of neoantigens. The availability of neoantigens is related to the overall number of mutations in the cancer genome (it is also described as tumor mutational burden or TMB). It is strongly correlated to the immunogenicity of different types of cancer, though recent studies are painting a more complex picture.^364,365^ Nevertheless, high levels of neoantigens are thought to explain why immunotherapy has had success with malignancies that arise from highly mutagenic processes such as melanoma (UV radiation) and lung cancer (smoking). Quite simply, fewer neoantigens implies that the immune system will have a harder time identifying and ultimately clearing cancer cells.

To combat this issue, Gu and co-workers deliver a hypomethylating agent (Zeb HMA) alongside PD-1 checkpoint antibody.^265^ Zeb HMA induces epigenetic changes to broadly activate expression of otherwise silenced genes, increasing the chance that cancer cells will begin manufacturing potential neoantigens. Local delivery of Zeb HMA to B16F10 tumors *in vivo* increased the amount of matured/activated DCs and decreased the number of immunosuppressive MDSCs within the TME. Flow cytometry also revealed that tumor cells began to up-regulate PDL-1 expression after Zeb HMA delivery with the ROS-scavenging hydrogel. This observation led to the inclusion of PD-1 checkpoint inhibitor antibodies (aPD-1) to further support the nascent immune response. However, rather than passively encapsulate aPD-1 within the gels, the antibodies were loaded first into pH-responsive CaCO_3_ nanoparticles that could then be physically entrapped within the hydrogel. The pH-sensitive nature of these particles would in theory both reduce acidosis within the TME and mediate selective intratumoral antibody release after the NPs released from the degrading hydrogel. Once combined, this triple-therapy gel was able to significantly increase the number of intratumoral CD8+ T cells, which correlated with superior overall survival. Notably, local combination therapy again resulted in improved outcomes compared to controls where Zeb HMA was delivered as a local bolus injection. This is perhaps in contrast to studies of local monotherapy, where the local sustained therapy is generally safer but provides comparable efficacy to local bolus administration. It will be important to see if future controlled local combination therapies see similar improvements in efficacy or whether this effect is due to the TME-modulating ability of this particular hydrogel platform.

Two of these studies provided critical data on the ability for local hydrogel therapy to illicit an abscopal effect on distant, untreated tumors.^265,361^ In both the B16F10 and 4T1 models, tumor growth is inhibited for both the treated and distant tumor. Increases in infiltrating T cells and APC activation occur in the distant tumor in both studies, supporting the mechanism of a systemic immune response to local immunotherapy. In addition to the abscopal effect, Gu and co-workers report that their therapy elicits immune memory and can protect previously cured mice from relapse after rechallenging them with fresh tumor cells.^361^ These data corroborate an earlier report by Wang and co-workers, which explored alginate hydrogel mediated codelivery of aPD-1 and the anti-inflammatory drug celecoxib in the B16F10 and 4T1 models.^366^ The combination of these two drugs proved to be synergistic, driving increased CD4+ and CD8+ effector T cell infiltration into tumors while depleting MDSCs and regulatory T cells. In the case of the 4T1 model, where spontaneous metastasis is common, hydrogel treatment mitigated the occurrence of distant metastases. Although alginate hydrogels do not confer any therapeutic benefits in themselves, in contrast to the ROS-reactive platform discussed previously, this study reported that hydrogel delivery improved the efficacy of aPD-1/celecoxib combination therapy compared to injection of the free drugs. This may support the notion that local therapy may provide greater benefit in the context of combination therapy, as opposed to local monotherapy, where the benefits are most often increased safety. Overall, these studies provide provocative data that supports the utility of highly local combination therapy for treating metastatic cancer.

#### 3.5.3 Hydrogels as Cancer Vaccines

Delivery of more ambitious combination therapy using injectable hydrogels has led to increasingly sophisticated and complex treatment regimens. Among the more comprehensive strategies has been to use hydrogels to deliver cancer vaccines, generally multicomponent therapies designed to kick-start *de novo* and durable immune responses by providing the key elements of antigen (sometimes preloaded into adoptive APCs) and one or more adjuvants.^367^ In this context, adjuvants are drugs that provide essential danger signals to the innate cells of the immune system (we have already discussed several adjuvants, including CpG and STING agonists). Cancer vaccines generally fall into two categories, those that mount a response to exogenous antigen and those that mount responses to endogenous antigen.^368^ The first case is perhaps the simpler approach in the context of preclinical research, where researchers can use a known neoantigen or a lysate of the tumor. However, the drawback here is in clinical translation, as this approach requires prior knowledge of a patient’s neoantigen repertoire or at least a biopsy of the tumor to generate the lysate or target antigen. The alternative strategy is to induce immunogenic tumor cell death *in situ* in order to release endogenous neoantigens to immune cells. While this approach is less biased and more easily applied in a clinical context, it introduces another layer of complexity (inducing productive immunogenic cell death) and it has proven to be quite difficult to successfully mount an effective, wholly endogenous immune response. Studies on schedule-dependency indicate that one major barrier here is that immunogenic cell death and antigen release may need to occur before innate cells encounter adjuvants.^344^

Mooney and co-workers have led pioneering studies into the use of injectable gels for cancer vaccines and have specifically leveraged the ability for these gels to recruit critical immune cells to maximize efficacy. By loading their hydrogels with chemokines that attract DCs, Mooney and co-workers recruit this critical APC and establish an immunogenic niche where DCs can preferentially engage with codelivered tumor lysate and CpG. The initial studies establishing the efficacy of this approach relied on noninjectable PLG scaffolds, which required surgical implantation.^369,370^ While not injectable, these systems facilitated extensive optimization of the components needed to generate a functional immunogenic niche. For example, the amount of the chemokine GMCSF determined whether enough DCs would migrate into the hydrogel, but too much and the DCs would not be able to return to regional lymph nodes to present antigen to resident lymphocytes.^369,371−373^ Likewise, optimization to evaluate different types of adjuvants provided helpful insights for mounting specific immune responses, identifying CpG and poly(I:C) as especially useful for mounting anticancer cellular immunity.^374^ This optimization facilitated the development of a new generation of injectable gels/scaffolds composed of alginate, mesoporous silica microrods, or gelatin to deliver the same therapeutic components (GMCSF, tumor lysate, and adjuvants) in a minimally invasive way.^375−377^ These extensive studies have revealed that the ability to recruit DCs into the gel is critical for hydrogel vaccine efficacy and can depend strongly on the microporous architecture of the hydrogel system being used.^378^ Further supplementing these cancer vaccines with systemic checkpoint inhibitors appears to be a facile and tolerable way to further boost efficacy and response rates,^379^ and future studies ought to compare whether including CPIs in the gel alongside the vaccine components is beneficial.

As hydrogel vaccines continue to become more sophisticated, greater control over the release of some or all of the vaccine components may unlock further benefits which would be inaccessible to traditional bolus administration routes. To mediate independent release of different drug cargo will likely involve the development of affinity hydrogels, which rely on supramolecular interactions with specific cargo to regulate differential release rates. In particular, DNA-based hydrogels provide a promising avenue toward this type of design, as DNA components can be individually engineered to introduce specific interactions via hybridization or other engineered affinities (e.g., aptamers).^293,294,380^

In the context of immunotherapy, DNA-based hydrogels can incorporate a number of nucleic acid adjuvants directly into the building blocks of the hydrogel itself. Nishikawa et al. demonstrated this capability with an injectable DNA hydrogel, which is self-assembled from DNA strands that contain immunogenic CpG repeats.^250^ The assembly of these gels arises from a two-stage self-assembly process. First, DNA strands hybridize into a DNA nanoparticle, termed a polypodna. Depending on the DNA sequence, polypodna of variable number of “arms” can be formed, and the arms can be engineered to feature overhanging single-stranded DNA, termed sticky ends. Under certain conditions (e.g., DNA polypodna concentration, ionic strength, and presence of a DNA cross-linker) the polypodna self-assemble into a network, thereby forming a hydrogel. This system offers a distinct advantage—that the effect of CpG delivery can be studied as delivered by a nanoparticle (nongelling polypodna) or as a hydrogel (gelling polypodna).

The initial study on this platform indicates that hydrogel formation was critical for the highest level of activity (e.g., type 1 cytokine induction and antigen specific antibody titers) *in vivo*. Moreover, this system can directly compare nonimmunogenic versions of the gel by replacing CpG with nonimmunogenic GpC motifs. As a result, this approach allows for a very thorough study of the role of adjuvant and its intersection with delivery vehicle. The inclusion of P32 radiolabeled nucleotides allowed for a highly quantitative assessment of CpG pharmacokinetics in the injection site and the blood compartment. Notably, the DNA hydrogels release ca. 90% of CpG locally over 36 h, while free CpG and polypodna released 90% of their payload in under 6 h. Consistent with the injection site data, the hydrogel formulations drive sustained accumulation of CpG into the blood over 48 h, while free CpG and polypodna exhibit early elevation in the blood compartment, which rapidly decays and flatlines by 12 h. This study highlights a very attractive mechanism for regulating the release rate of nucleic acid-based adjuvants—a broad class that includes drugs such as CpG (TLR 9 agonist), poly(I:C) (TLR3 and RIG1 agonist), and ssRNA (TLR7/8 agonists). It could be fruitful to also explore systems that can transfect cells locally, which could induce the *in situ* production of many more immunomodulatory proteins.

DNA gels offer a straightforward way to incorporate and regulate the release of functional nucleic acids but also offer opportunities based on their intrinsic negative charge. Nishikawa and co-workers reported that cationized ovalbumin could be loaded into these gels and retained over a longer time frame than unmodified ovalbumin (ca. 24 h vs 3 h *in vivo*).^254^ Mice treated with hydrogels made of CpG-containing DNA and loaded with cationized antigen were more effective at slowing the growth of established tumors expressing the target antigen, compared to free formulations or CpG gels loaded with unmodified antigen.

These data were later corroborated by Li and co-workers in a similar study, which also described DNA hydrogels capable of mediating controlled release of CpG and cationic antigen.^381^ In this system, DNA strands self-assemble into Y-shaped building blocks that are cross-linked by a linear DNA linker composed of CpG. In this case, a short 20-aa peptide for the MUC1 tumor-associated antigen was fused to another short 21-aa P30 peptide, which has been reported to be a T-helper cell epitope. Importantly, the linkage between the two peptides is a string of lysine residues, providing an overall cationic charge which provides the electrostatic affinity to the DNA gel and thus a slow and sustained release profile. This hydrogel vaccine is effective at slowing growth in the challenging B16F10 melanoma model and outperforms the same drug cocktail provided as a bolus injection. While the cellular response was not fully explored, interesting data regarding antibody generation was gathered. In particular, antibody class-switching and dominant immunoglobulins were characterized for the CpG gel loaded with antigen versus the free antigen. While this comparison only provides limited insight, it did reveal a skew toward IgG1 and IgG2 isotypes in the gel vaccine, as opposed to an IgG3/IgM dominated response with antigen alone. This difference is likely due to the lack of an adjuvant in the control, but future studies ought to compare how free and gel-based vaccines may reshape class-switching during the humoral response.

Toward cancer vaccines that mount *in situ* or endogenous immune responses, researchers have taken a second look at the combination of specific chemotherapeutics and complementary immunotherapeutics.^382^ The initial studies described above typically focused on chemotherapy in conjunction with cytokines or CPIs, leaving a critical part of the immune cycle unsupported—the activation of APCs through adjuvants. Liu and co-workers recently reported that local therapy with immunogenic chemotherapy and TLR7 adjuvant produces a robust immune response, which can then be augmented with an additional immune checkpoint blockade.^383^ In this study, injectable alginate hydrogels were loaded with R837 (aka imiquimod), a small molecule agonist of the TLR7 pathway. In addition, either doxorubicin or oxaliplatin, both of which induce immunogenic cell death, were coloaded into these hydrogels. Flow cytometry analysis of CT26 tumors treated with this chemoimmunotherapy hydrogel showed significant increases in DCs, TAMs, and T cells (both CD4+ and CD8+). As expected, the inclusion of the R837 adjuvant into the hydrogel led to a considerable increase in activated APCs (ca. 50% increase in frequency). Interestingly, inclusion of the adjuvant also led to an increase in PDL-1 expression on cancer cells, DCs and TAMs, as well as an increase in PD-1 in T cells, which justified the subsequent inclusion of a PD-L1 antibody. The addition of anti-PDL-1 led to a robust immune response, including the formation of memory T cells, regardless of whether the antibody was included in the hydrogel or administered systemically. Impressively, this triple-combination therapy was broadly effective in the CT26 colon, 4T1 breast, and the P5 C57 glioma cancer models, leading to long-term survival. Critically, the local therapy was also able to mediate abscopal effects on distant tumors in all three models. This study provides a very promising approach for driving *in situ* or endogenous immune responses to tumors, without the need to deliver a known neo-antigen or tumor-specific cell lysate. In general, these types of approaches are exciting from a clinical perspective, as they might be suitable to treat a variety of tumors using a combination of drugs that clinicians are already quite familiar with.

#### 3.5.4 Hydrogels as Adjuvant Therapies

Local immunotherapy is potentially very useful as an adjuvant therapy, a medical term used to describe a treatment which is given in addition to a primary treatment. Somewhat confusingly, it has no relation to the concept of the adjuvant class of drugs used in formulating vaccines. In the clinic, adjuvant therapy is often a medical treatment that supplements surgical resection or complements radiotherapy. Traditionally, adjuvant therapies have been regimens of chemotherapy provided before (in which case it is called neoadjuvant therapy) or after the “primary” treatment.^384^ But since surgery and radiotherapy can cause a great amount of immunogenic cell death (thereby making neoantigen available) and can mitigate or eliminate immunosuppression, complementing these treatments with immunotherapy has become an area of intense interest.^385−387^

In the case of surgery, the wound bed is an attractive area for local immunotherapy. Even traditional, noninjectable hydrogels can be very useful in these scenarios. Nevertheless, thixotropic hydrogels continue to provide unique advantages in this area, particularly since certain formulations can be applied through spraying techniques to optimally cover all exposed surfaces. Gu and co-workers demonstrated the potential of sprayed immuno-stimulatory hydrogels for adjuvant therapy by using a fibrin gel loaded with CD47 antibody-carrying CaCO_3_ nanoparticles.^388^ As nanoparticles are released from the hydrogel, they break down in the acidic tumor microenvironment where they simultaneously release antibody and shift pH toward neutral. The pH normalization appears to rewire the local immune cells, as delivery of the nanoparticle alone is sufficient to deplete MDSCs and T regulatory cells in the tumor. Combined with CD47 antibody, which stimulates the phagocytosis of tumor cells, this treatment mounted a strong immune response that protected mice from postresection recurrence in the B16F10 model of melanoma. Furthermore, these gels were able to slow the growth of distant tumors in a model of incomplete resection, indicating the ability to mediate abscopal effects.

In the case of radiation therapy, injectable hydrogels are the preferable approach for mediating a local adjuvant therapy through minimally invasive means. Liu and co-workers explored this combination in a unique way by using a sodium alginate hydrogel to intratumorally deliver multicomponent therapy that simultaneously relieves tumor hypoxia, mediates local radiotherapy, and stimulates innate immune cells.^389^ This approach delivers catalase, an enzyme that generates oxygen from intratumoral ROS species, to reverse hypoxic conditions. However, this group took the innovative step of labeling catalase with the ^131^I radioisotope to provide local radiotherapy from their hydrogel. By combining the delivery of radioactive catalase with CpG, the treatment was able to mount a strong immune response in four tumor models: the murine 4T1 breast cancer, murine CT26 colon cancer, prostate patient-derived murine xenograft, and rabbit VX2 liver cancer models. The response was sufficient to eradicate tumors in all models with radioisotype therapy, but metastatic models required CpG and additional systemic anti-CTLA4 treatment to increase survival. This transformative approach leveraged hydrogel technology to deliver both the primary treatment (radioisotope therapy) and several additional local adjuvant therapies (antihypoxia enzyme and CpG). Notably, the radiation therapy was effective using a low dose of radioisotope, which may indicate that this approach could be used to more safely administer radiotherapy.

Along these lines, several innovative hydrogel therapies have been developed that bundle light-triggered phototherapy.^390−392^ In these cases, reactive elements in the hydrogel generate heat or cytotoxic byproducts when stimulated with light at a specific wavelength and can mediate immunogenic cell death similar to radiotherapy. Again, in these treatment regimens, the “primary” photothermal treatment leverages the hydrogel carrier to focus its effect within or near the tumor. The production of heat or chemical byproducts can also be leveraged to trigger the release of the secondary cargo, in this case immunostimulatory drugs. Nishikawa and co-workers took this approach using their injectable DNA polypod hydrogels to deliver light-reactive gold nanoparticles.^390^ After injection, the DNA gel could be irradiated with near-infrared wavelengths to generate heat and induce cell death. The heat also triggered release of DNA from the gels, which could be encoded to contain CpG motifs to simulate innate immune cells. Together, this therapy suppressed the growth of EG7-OVA tumors in mice.

Jia et al. recently explored using this style of multifunctional hydrogel as a supplement to surgical resection, essentially bringing together surgical, photothermal, and immunotherapeutic capabilities into one treatment regimen.^392^ This group used an injectable temperature-sensitive hydrogel to deliver nanoparticles loaded with a photosensitizer drug (ICG) and two immunostimulant drugs (CpG and R848). After resection, the hydrogel was injected into the surgical bed, where it conformed to the geometry of the incision as it gelled. Irradiation with near-infrared light initiated photothermal therapy to kill residual tumor cells and release tumor antigen. Phototherapy here also triggers release of CpG and R848, providing the components necessary for an endogenous cancer vaccine. Mice treated with hydrogels containing the immunotherapeutic drugs had more mature DCs and CD8+ T cells in tumor draining lymph nodes, which correlated with lower incidence of distant metastases following surgical resection in the 4T1 model of breast cancer. Overall, these approaches provide an innovative means to trigger effective immunogenic cell death, making the development of endogenous cancer vaccines more feasible. Critically, the ability for these treatments to mount abscopal effects to treat distant metastasis could make surgical debulking plus local immunotherapy a viable treatment for patients initially diagnosed with metastatic disease, who would normally not be candidates for surgical treatments.

### 3.6 Hydrogels for Immunomodulation Beyond Cancer Immunotherapy

There is no question that mobilizing the immune system toward treating disease is quickly becoming a pillar of biomedical research. The current massive expansion of research efforts toward immuno-oncology was in response to the remarkable, curative results from recent clinical trials.^393,394^ And with the global fallout from the SARS-CoV-2 pandemic, we expect to see another wave of explosive growth to develop next-generation immunotherapies for infectious disease applications. However, future research should also consider other important but often overlooked biomedical applications such as the treatment of autoimmune disorders. Here, we summarize some key findings for hydrogels in this broader immunotherapy space.

#### 3.6.1 Injectable Hydrogels as Vaccines for Infectious Disease

While the main focus of a previous section was cancer vaccines, it is critical to note that hydrogel carriers may be equally beneficial for vaccines against infectious disease. Given the devastating impact of the SARS-CoV-2 global pandemic, this area is likely to garner significantly more attention in the future. Nevertheless, there are sufficient data now to support the theory that systems capable of sustaining the release of antigen over prolonged periods of time are able to induce antibodies with improved specificity and neutralizing capabilities.^395^ The fundamental basis for this theory comes from studies using osmotic pumps to sustain the release of antigen over time in rhesus monkeys.^310^ In general, it appears that approaches that can mimic the antigen release kinetics typical to natural infection are able to prolong the process of somatic hypermutation in regional lymph nodes, yielding superior antibodies. While these results are promising, implantation of osmotic pumps is impractical in a clinical setting, particularly in areas of the world where infectious diseases are most prevalent. A potential solution to this problem will be injectable hydrogels, which can sustain release of cargo in an optimal way. Early studies showed that thermosensitive polymeric hydrogels can be used in place of typical carriers such as Freund’s adjuvant to deliver antigen.^396^ While the antibody response to these antigen-loaded gels is inferior to Freund’s adjuvant, it is important to note that Freund’s is innately immunogenic whereas the hydrogels used in this study were unlikely to provoke an immune response. This highlights the need to deliver immunomodulatory drugs alongside antigen in hydrogels to maximize their potential. For example, when delivering CpG alongside antigen, DNA hydrogels drove effective antibody responses, while producing less toxicity than Freund’s adjuvant or alum carriers.^250^

**Figure 26:**
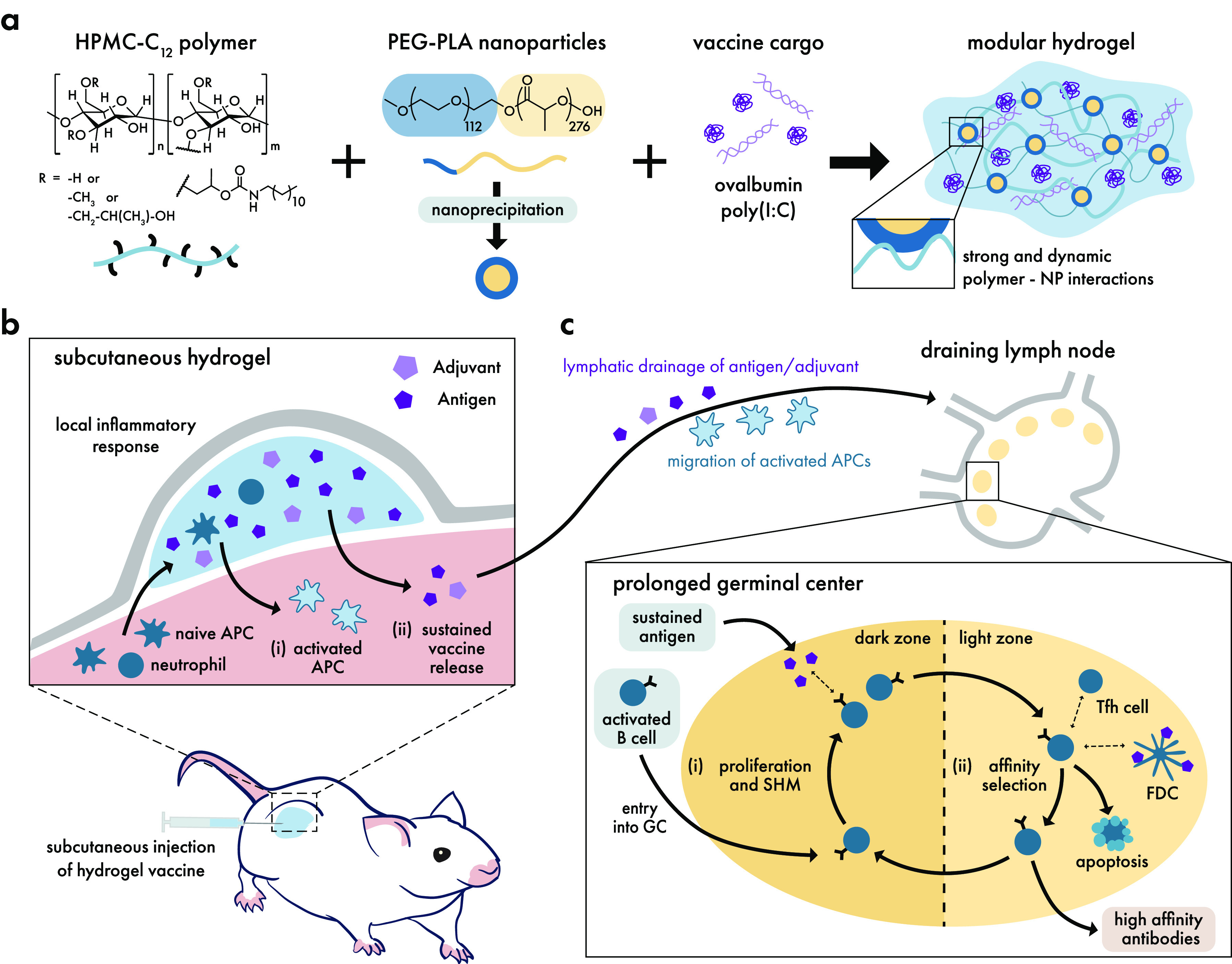
Sustained release of vaccines from hydrogels drives prolonged germinal center activity. (a) Injectable polymer–nanoparticle hydrogels are formed through dynamic interactions between modified hydroxypropyl methyl cellulose polymers and PEG-PLA nanoparticles, and bulky hydrophobic cargo can be loaded in the aqueous phase of the gel. (b) Subcutaneous injection of the gel creates a depot for vaccine components (antigen and adjuvant), encouraging infiltration by immune cells while simultaneously releasing antigen and adjuvant into the surrounding interstitial fluid. (c) Vaccine components and activated antigen presenting cells reach draining lymph nodes to drive an immune response. Maturation of antibodies occurs in the germinal centers of the draining lymph node, where sustained antigen exposure facilitates the process of somatic hypermutation. In our studies, these hydrogel vaccines improved the humoral response, leading to antibodies with significantly greater affinity toward their target molecules. Adapted with permission from Roth et al.^397^ Copyright 2020 American Chemical Society.

More recently, our group reported injectable polymer–nanoparticle hydrogels for vaccinations, which could significantly slow the release of incorporated antigens and adjuvants (Figure 26).^397^ Tuning the polymer and nanoparticle content of these gels influenced the relative release rates of ovalbumin and poly(I:C) adjuvant, with the optimized formulation releasing both components at similar rates, and sustained *in vivo* release over the course of 4 weeks. Slow-release hydrogel vaccines were able to drive prolonged germinal center responses in draining lymph nodes out to 30 days postprime, compared to bolus (low activity by 15 days postprime) and fast-release gel vaccines (low activity by 30 days postprime). Most notably, slow-release hydrogels induced antibodies with a 1000-fold increase in antigen-specificity, compared to bolus vaccination. Interestingly, cellular infiltration into the gels was strongly influenced by the presence of cargo, with empty gels containing 5-fold fewer immune cells than vaccine-loaded gels. Moreover, vaccine gels recruited more monocytes, macrophages, and dendritic cells—all cell types with professional antigen presenting capabilities—than empty gels. Among dendritic cells, the majority were migratory cDC2 cells that have been reported to be important activators of follicular helper T cells, which play a central role in antibody affinity maturation.^398^ Overall, these results largely support the predictions based on sustained release from prior osmotic pump studies and indicate hydrogels may be a promising path toward translating the benefits of sustained release vaccination into the clinic. Future work in this area may illuminate means to develop improved vaccines and also reveal critical biology related to the release kinetics of individual vaccine components.

#### 3.6.2 Hydrogels for Initiating Immune Tolerance

Aberrant activation of the immune system leads to an array of devastating diseases that include type 1 diabetes, multiple sclerosis, arthritis, and lupus. So far, there have not been many attempts to leverage hydrogel technologies to address these diseases. However, one of the few reports on this subject described promising results using a tolerogenic hydrogel vaccine for type 1 diabetes. Keselowsky and co-workers used a puramatrix peptide hydrogel to locally deliver PLGA microparticles loaded with insulin self-antigen.^399^ The hydrogel was also loaded with GM-CSF and CpG, which could attract immune cells and activate them. It is worth noting that in the context of this study, CpG was explored for its reported ability to induce tolerance, which is surprising given its usefulness in anticancer studies. This is perhaps an important reminder of the complexity of the immune system and the ability for molecules to have pleiotropic effects;^400−405^ that is when the same molecule can induce different (and at times opposing) effects depending on the biological context. After three subcutaneous injections, this insulin-tolerizing hydrogel vaccine protected 40% of NOD mice from developing type 1 diabetes. Although this study documented increased anti-inflammatory IL-10 due to the treatment, deeper details of the underlying mechanism remain unknown. Engineering tolerance through materials-based interventions is a fertile research area, and we anticipate future studies will provide important insights into the immune system and potentially transformative therapies for autoimmune disorders.

## 4 Hydrogels for Cellular Therapy

**Figure 27:**
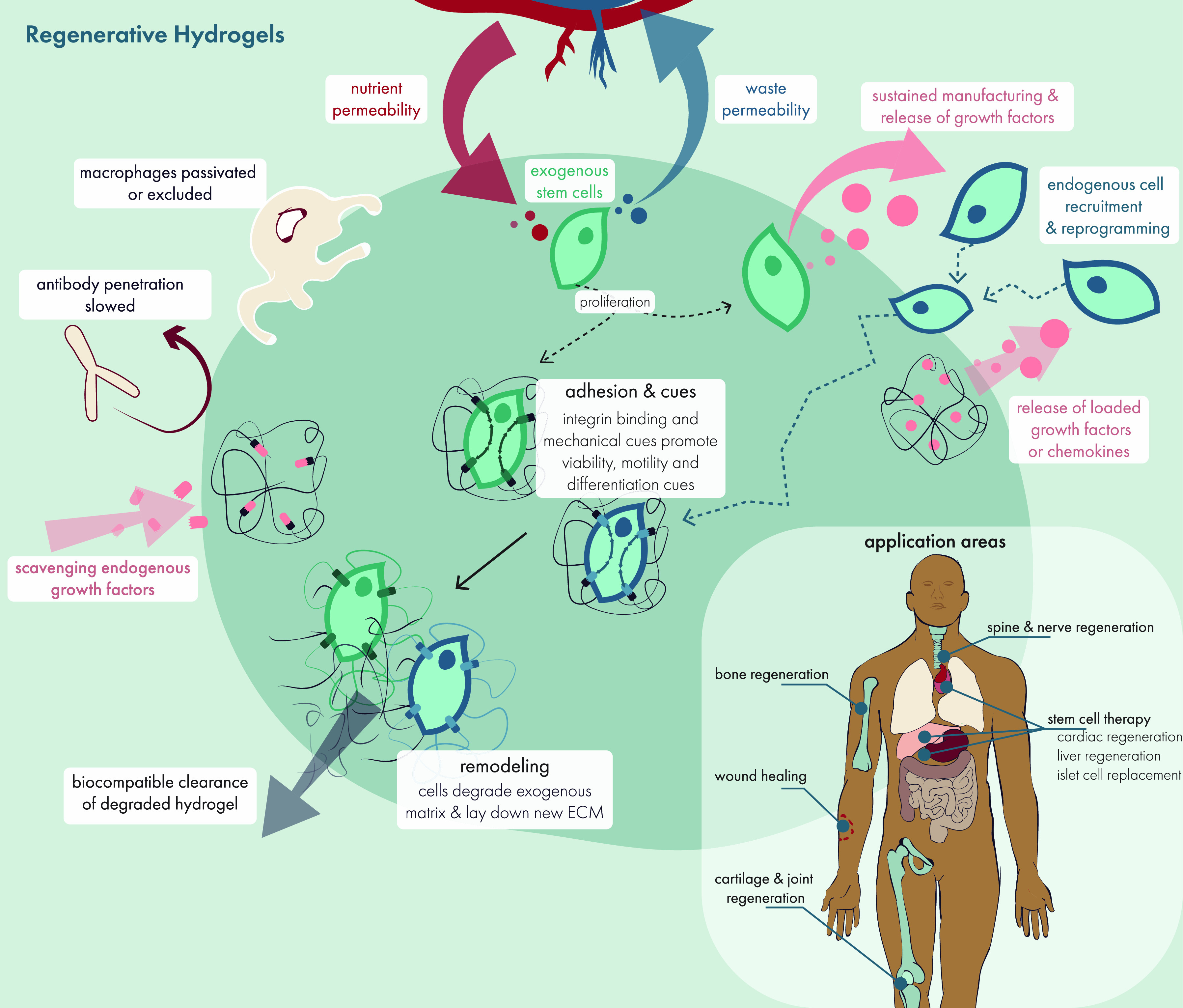
Hydrogels can act as both a scaffold for endogenous cells and a delivery vehicle for exogenous therapeutic cells, like stem cells. Successful hydrogel formulations can simultaneously mediate multiple functions to create a microenvironment conducive to tissue regeneration. The hydrogels must be sufficiently porous to allow nutrients to reach cells that are inside of them, as well as permit efflux of waste products as those cells metabolize nutrients and continue proliferating. Scaffolds that provide adhesion motifs to engage with cells can provide critical mechanical cues to bolster viability, proliferation, and motility. In certain cases, it is also important for the hydrogel to prevent elimination of exogenous cells by the immune system by, for example, excluding macrophages or antibodies. Hydrogels that can successfully protect their cargo can signal to the local environment either through paracrine signaling from exogenous cells or release of preloaded bioactive factors. Ultimately, these hydrogels also permit resident cells to degrade the hydrogel matrix and lay down their own extracellular matrix (ECM). Injectable regenerative hydrogels have applications for treating a wide range of diseases and injuries. Original illustration.

Aside from drug delivery, hydrogels can also be engineered into scaffolds for native and exogenous cells, providing three-dimensional templating and structure useful for tissue regeneration and adoptive cell therapy. For example, careful design of hydrogel materials to encourage beneficial cellular infiltration and expansion can drastically change biological outcomes in regenerative treatment (Figure 27). Current approaches tune the mechanical and chemical properties of hydrogels to more closely mimic native extracellular matrix, developing substrates with improved control over cellular growth and differentiation. Many hydrogel-based cellular scaffolds are composed of natural materials, such as collagen or alginate, but more recent work has focused on developing highly defined and mutable synthetic materials, such as polyethylene glycol. These hydrogels can be further augmented to deliver helpful biologics, such as chemokines or growth factors that drive cellular differentiation toward desirable end points. While many hydrogels recruit and support endogenous cells to accomplish their goals, there are also hydrogels that are proving quite useful for delivering exogenous therapeutic cells (e.g., stem cells or adoptive T cells).^406^ In this section, we review the properties that make hydrogels effective ECM mimics and excellent carriers for therapeutic cells. We also explore the ways that hydrogels can improve cell delivery before, during, and after injection compared to traditional liquid injections.^407,408^ And finally, we summarize the advances for specific and diverse cellular therapy applications.

### 4.1 Cell Adhesion to Hydrogels

To live, proliferate, and migrate, many cells require integrin engagement with a matrix material. Without this critical cue, most cells undergo a specialized form of programmed cell death known as “anoikis”, a term which means “a state of being without a home”. Cells are “at home” in the native ECM, where they can attach to cell-adhesive motifs distributed throughout the ECM network. To mimic this kind of cellular “home”, hydrogels can be engineered with ligands (e.g., peptides and certain polysaccharides) to promote cell attachment and enhance viability, and otherwise cue specific cellular programming. In general, inclusion of adhesion motifs promotes cell engagement with the scaffold and helps to increase cell viability and proliferation within the scaffold.^409−411^ These motifs are also useful for changing cell motility within the hydrogel,^23^ which can be useful for promoting cell egress or retention from hydrogels. In a sense, adhesion motifs open a channel of communication with cellular cargo or infiltrates. And depending on the type of motif, its density, or its spatial patterning, hydrogels are able to coax different behaviors out of cells in order to meet the particular goals of a given cellular therapy.

To unlock the potential for adhesion motifs in hydrogels, researchers generally search out the means to stably incorporate them into the polymer network. Naturally derived hydrogels (including collagen, gelatin, hyaluronic acid, and fibronectin) are intrinsically recognized by several cellular receptors and thus enable cell attachment without any further modification.^53^ In contrast, synthetic hydrogels must be modified to include cell adhesion motifs to promote attachment. Although this adds additional materials processing steps, it is also an opportunity to design matrices with novel motifs, or combinations of motifs, that may not be available from natural substrates. As a separate but equally interesting line of inquiry, engineered systems also allow us to more directly ask questions about the role of ligand/motif density in hydrogels and, as proxy, the native ECM.

Although there are many potential cell adhesion ligands, the majority of work has focused on a few amino acid motifs (e.g., the fibronectin-derived RGD sequence or the laminin-derived IKVAV sequence) that have been shown to be quite effective in promoting cell attachment.^410^ For example, 500 μM to 1 mM concentrations of RGD are effective in promoting stem cell attachment.^409^ Along these lines, bioactive nanofiber peptide amphiphiles modified with RGD promote increased viability of bone marrow derived stem and progenitor cells. In an *in vivo* model with bioluminescent cells, this RGD-modified material produced a 3.2-fold increase in bioluminescence compared to cell delivery using a saline bolus.^412^ In another example of the utility of RGD-modified gels for *in vivo* tissue regeneration, Heilshorn and co-workers developed the “Mixing Induced Two Component Hydrogel” (MITCH), which is composed of two protein engineered block copolymers that interact in a 1:1 stoichiometry.^413^ These peptides were engineered to contain an RGD motif to promote cell adhesion and viability, and they showed enhanced adipose stem cell retention in the subcutaneous space compared to unmodified alginate and collagen.^413^ Overall, the inclusion of cell adhesion motifs is a significant and quite effective approach for engineering the material–host interface. From a design perspective, these motifs provide a very broad parameter space for designing cellular scaffolds, even when considering that research has mostly focused on a subset of possible ligands. As materials synthesis techniques continue to improve and become more compatible with high-throughput discovery approaches, we anticipate interesting outcomes to studying materials libraries that probe novel ligands, combinations, and spatial patterns.

### 4.2 Hydrogel Degradability to Facilitate Cellular Remodeling and Motility

In addition to attaching to the hydrogel matrix, cells also reorganize the matrix by degrading the hydrogel mesh and depositing their own ECM, reshaping their microenvironment as they mature, proliferate, and migrate. For hydrogels to accommodate proliferation and migration, they must be engineered to degrade in a controlled manner. For example, a hydrogel can facilitate cellular migration by including protease-sensitive elements in the hydrogel network, which the cells can degrade “on command” by secreting the relevant proteases.^414^ Protease-sensitivity is a fairly universal approach to controlled degradation, but it is especially useful in chemically cross-linked systems.

On the other hand, cellular migration through dynamic hydrogels (particularly those lacking macroscopic porosity) is still not completely understood, but recent reviews have put forth several potential mechanisms by which these systems may permit cellular migration.^93,415^ In general, it is thought that the dynamic formation and dissociation of cross-links leads to transient openings or migratory pathways for encapsulated cells. Leading theories to develop design criteria for these systems therefore focus on the thermodynamics and kinetics of dynamic cross-link formation, which need to strike a balance that provides physical stability without preventing cell migration.^93^ If cross-link rearrangement occurs too quickly, for example, then cells may not have time to spread or migrate before cross-links are re-established. If it is too slow, the hydrogel cannot provide the adequate support needed to provide homogeneous cell encapsulation or retain cells at the injection site. With this in mind, understanding the thermodynamics of cross-link formation is a critical factor for designing dynamic hydrogels friendly to cellular migration or infiltration. However, assessing cross-link thermodynamics in the absence of cells may paint an incomplete picture; there is a good deal of evidence that the thermodynamics and kinetics of dynamic cross-link formation can be temporarily disturbed or altered by the mechanical forces exerted by migratory cells.^93^ Future studies observing single-cell migration through these systems may begin to shed more light on how specific cell types may be able to move through dynamic networks and to what extent these networks need to be degraded to facilitate migration.

Regardless of the network chemistry, eventual degradation is still a desirable trait since resorbable materials tend to be most biocompatible in the long run. Fortunately, many natural hydrogel materials such as collagen and gelatin degrade into safely metabolizable and excretable base components. Some natural polymers, however, are not innately degradable. For example, alginate is nondegradable in mammals, which lack the necessary enzyme alginase.^416^ And while ionically cross-linked alginate gels still dissolve *in vivo* due to the release of the divalent cross-linker ions, the dissolved alginate polymers are often larger than what can be cleared through the kidneys.^417^ This particular problem has been solved by partial oxidation of the alginate backbone, which yields a highly degradable polymer.^418^ Interestingly, this improved degradation also led to increased cell infiltration *in vivo*, highlighting the important relationship between degradability and cell motility.^419^

To make synthetic materials susceptible to degradation, hydrogels can be engineered to include specific stimuli-sensitive chemistries (e.g., degradation driven by changes in pH, ROS, proteases, or exogenous triggers) or hydrolytically degradable chemistries.^420^ One of the major benefits to the stimuli-responsive forms of engineered decomposition is that it can be tailored to the specific cell delivery applications being explored. For example, using an amine reactive cross-linker, Madl et al. were able to independently control degradability from stiffness in hydrogel materials.^421^ Using this system, the authors found that protease-mediated degradability directly aided in the maintenance of stemness of neural progenitor cells in different hydrogel materials, while stiffness played little role. Overall, hydrogels that facilitate cellular remodeling, either through direct degradation by cells or through other more passive mechanisms, may provide unique advantages for regenerative cellular therapies.

### 4.3 Tuning Diffusion and Porosity

Diffusion and porosity are key design considerations when engineering hydrogels as cellular scaffolds. Cells require sufficient oxygen and glucose in order to survive, and hydrogels present a literal barrier to these necessities. Studies have found that both the hydrogel mesh size and cell density are major contributors to diffusion of nutrients within a hydrogel. As a general rule of thumb, studies have shown that a mesh size of less than 15 nm and cellular densities greater than 4 million per mL begin to impede nutrient diffusion, but these studies are dependent on cell type size and characteristics.^422,423^ However, not all hydrogels have an easily defined mesh size, such as hydrogels based on transient dynamic cross-links, and in these cases it may require empirical viability and transport experiments to determine the limits on nutrient diffusion.^69^ From the point of view of translation, it is also important to consider how geometries may scale from preclinical to clinical studies. In these cases, a small volume of hydrogel may work well to deliver cells without the threat of nutrient-deprivation in preclinical murine studies, but human studies may require much larger volumes that will increase the distance that nutrients will need to travel within the gel.

In addition to limiting diffusion of nutrients, hydrogels can also limit diffusion of coencapsulated factors or drugs. In this scenario, hydrogels are a diffusion barrier, keeping exogenous growth factors local to the delivered cells to drive cellular growth or differentiation. Under these conditions, retention of cargo and intake of endogenous nutrients become opposing design criteria. Fortunately, there are ample strategies from the field of hydrogel drug delivery to decouple the diffusion of a delivered factor and ambient nutrients. For example, if a hydrogel carrier cannot slow diffusion of cargo enough through passive release alone, those factors may be conjugated directly to the hydrogel material to keep them local to the scaffold.^202,424^ This technique is very similar to the natural capability of endogenous ECM to bind to, and retain, specific growth factors. It is worth noting that cargo tethering, and other techniques discussed in the earlier drug delivery sections, could be leveraged to decouple the movement of endogenous and exogenous factors through the gel.

In addition to engineering around diffusion requirements, designing distinct microstructures (e.g., porosity) into hydrogels can influence cell function and hydrogel mechanical strength. Electrospinning has been used to make nanofiber-based hydrogels from hyaluronic acid. The unique fibrous morphology of these hydrogels influences chondrogenic differentiation and cell alignment for cartilage engineering applications.^425^ Microribbon-like elastomer-based hydrogels have been developed from wet-spinning gelatin to form hydrogels and using methacrylates to cross-link a swollen microribbon-based network.^426^ This method produces hydrogels with a highly distinct macroporosity and remarkable shock-absorbing mechanical properties. *In vivo*, these microribbon-like hydrogels demonstrated impressive results in cartilage tissue regeneration.^426^ Injectable granular hydrogels based off hydrogel microparticles are another emerging technique to introducing macroporosity to promote cell growth. Microparticles are fabricated using microfluidics, emulsions, or mechanical fragmentation and then concentrated to form a hydrogel. Cells can be encapsulated within microparticles or encapsulated between packed microparticles.^427^ Segura and coworkers showed that the injection of a granular hyaluronic acid hydrogel into a stroke-formed cavity reduces the inflammatory response while increasing peri-infarct vascularization compared to nonporous traditional hydrogel controls.^428^ These studies broadly highlight how certain physical traits (e.g., porosity) have wide-ranging effects on multiple properties of the gel, in this case nutrient transport, cell motility, and mechanical resilience.

### 4.4 Cell Delivery Using *In Situ* Gelation

Many hydrogel-based cellular scaffolds are designed to gel *in situ* after injection, which introduces some specific materials design constraints. Depending on the chemical gelation strategy, gelation can potentially lead to cytotoxicity if the chemistries used are not biorthogonal.^408^ For that reason, *in vitro* cell viability studies are normally performed prior to *in vivo* studies to evaluate if the material chemistries enable cell growth.^429^ The type of trigger for gelation can also determine the kinetics of gelation, particularly if it involves a period of equilibration with an environmental stimulus. Often, triggered gelation strategies involve changes in temperature,^430^ light, pH, or ion concentration^419^ after injection. For the popular thermogels, these formulations often include temperature-sensitive polymers with lower critical solution temperatures that gel at body temperature after injection. Poly(*N*-isopropylacrylamide) (PNIPAM) is a common polymer used in these hydrogel formulations due to its LCST phase transition at approximately 32 °C. So while liquid at room temperature, these systems quickly gel as they warm to 37 °C.^96,431^ Hydrogels composed of decellularized matrix often demonstrate temperature induced gelation at 37 °C while also presenting many natural bioactive molecular motifs that can promote cell attachment and growth.^432,433^ These materials can be directly harvested from donor tissue and decellularized, but there are issues with batch-to-batch variability between donors that complicate the clinical translation of decellularized scaffolds.

Many triggered hydrogel systems incorporate methacrylate-based chemistries to photopolymerize upon light or UV exposure. For example, many biopolymers such as alginate, hyaluronic acid, gelatin, and chitosan have been modified with methacrylate groups to enable triggered gelation with photopolymerization after injection.^434^ However, it is necessary to pay close attention to the cytotoxicity associated with radical initiators and prolonged UV exposure.^435^ And from a translational perspective, it is challenging to use light-triggered gels in deep tissues that are inaccessible to the short wavelengths typically used. As a result, light-triggered gelation may need to focus on developing infrared or near-infrared triggers, which can penetrate deeply into the body, in order to be translated for deep-tissue biomedical applications.

To successfully deliver cellular therapies, triggered gelation must occur quickly enough to prevent cell settling and retain cells at the transplantation site after injection.^436^ When delivering exogeneous cells, hydrogels that gel *in situ* after injection (as compared to dynamic hydrogels that are shear-thinning) do not possess the favorable mechanical qualities that stabilize and protect cells before or during injection, but they do provide mechanical protection of the cells immediately after injection and long-term. For example, once gelled, the hydrogels protect cells from being swept away from the high-pressure environments within the injection site.^437^ Longer term after injection, hydrogels help cells to persist at the delivery location by acting as scaffolds that support proliferation and growth in 3D, as discussed above.

### 4.5 Cell Delivery Using Dynamic Hydrogels

**Figure 28:**
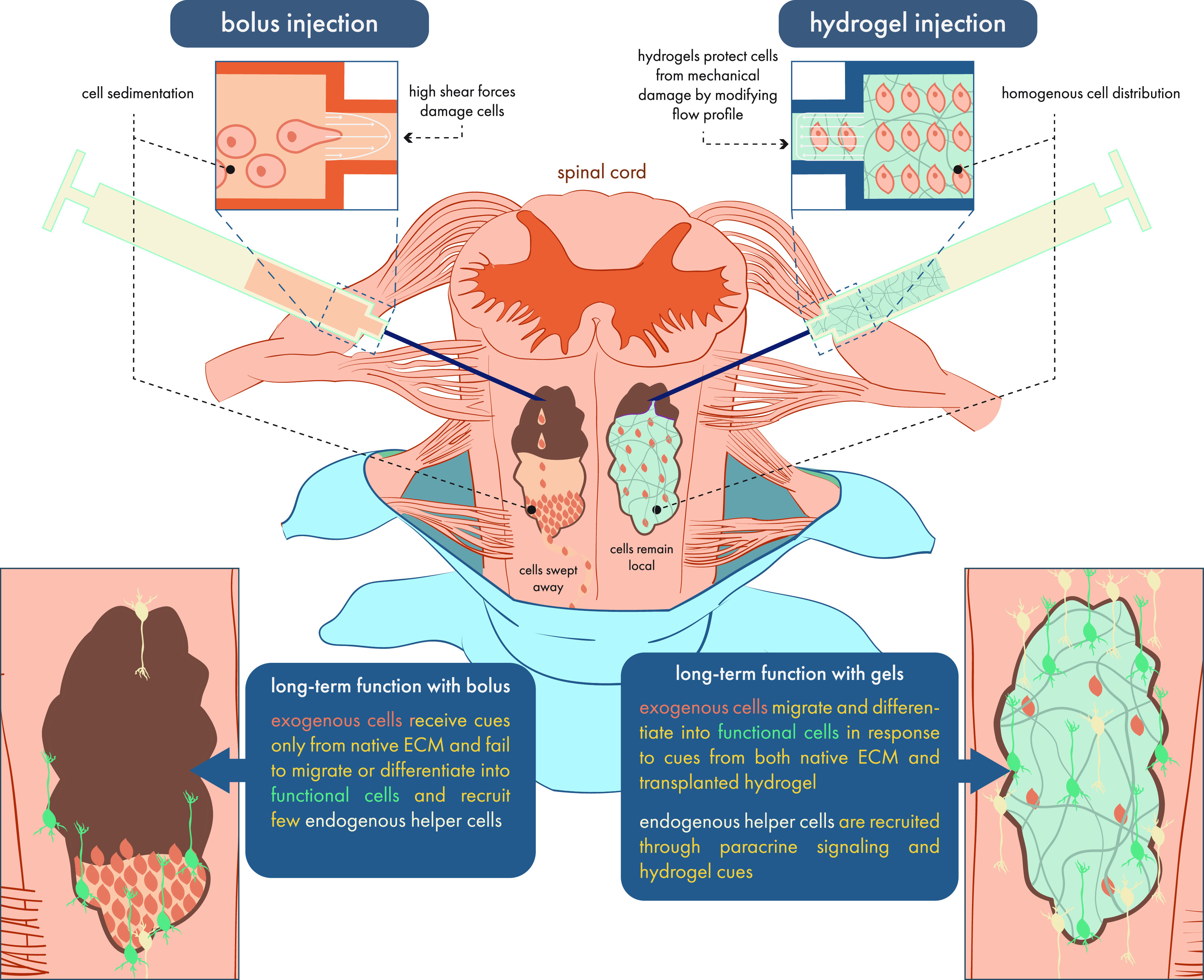
Dynamic hydrogels provide benefits before, during, and after injection of therapeutic cells. Bolus injections of cells suffer from cell settling in the syringe, which can lead to inhomogeneous dosing. In contrast, dynamic hydrogels are solid-like in the syringe before the application of pressure, which maintains cells homogeneously distributed throughout the medium. During injection, bolus formulations expose cells to high mechanical forces and shear that can compromise their viability. Dynamic hydrogels shield cells from those forces and increase the number of viable cells delivered to the target tissue. After the injection, cells administered by bolus administration have proliferated based solely on cues from the endogenous tissues, limiting their regenerative potential as well as the recruitment of endogenous cells. Dynamic hydrogels, on the other hand, can be designed to include molecular and mechanical cues that provide additional long-lasting stimulation for both exogenous and endogenous cells, driving greater function and proliferation. Original illustration inspired by the work of Heilshorn and co-workers.^407^

As discussed in prior sections, dynamic hydrogels have been designed that involve reversibly cross-linked networks, giving rise to shear-thinning and self-healing materials that can be injected even after gelation (Figure 28). Shear-thinning hydrogels for cell transplantation have been designed using chemistries including alginate, engineered protein assemblies, polymer–nanoparticle interactions, dynamic covalent bonds, and host–guest interactions.^438−441^ Like *in situ* gelation approaches, dynamic hydrogels improve cell viability and retention at the transplantation site. However, dynamic hydrogels can also maintain cell viability before and during injection due to their unique rheology. Before injection, dynamic hydrogels exhibit solid-like properties within the syringe or delivery device, which maintains cells homogeneously suspended throughout the medium, leading to more reproducible and consistent cell delivery.^437^ During injection, shear-thinning hydrogels protect cells from destructive shear and extensional forces exerted within syringe needles to prevent damage to cell membranes. This ability to safely shepherd cells through the injection process leads to improved viability after injection with dynamic hydrogels, compared to liquid carriers.^108,442^ Aguado et al. demonstrated this phenomenon by comparing hydrogel and liquid carrier injection methods, and they found that up to 40% of cells were destroyed during syringe needle injection with a liquid carrier, in contrast to the ca. 5% loss seen with hydrogels.^442^ The authors hypothesized that the plug flow profile of shear-thinning hydrogels within the syringe helped to protect cells from damaging mechanical forces. That being said, the mechanisms behind this phenomenon are still being understood and are an active area of ongoing research.^437,443^

Other types of shear-thinning materials include dynamic covalent hydrogels, which use covalent bonds capable of reversibly exchanging, dissociating, or switching.^444^ These materials are generally stronger than many physically cross-linked systems, which provides both opportunities and challenges for cellular therapies. Stiffer and stronger scaffolds can provide mechanical cues more suitable for certain cells (e.g., bone cells) or better retain cells in specific shapes and conformations. For example, Wang et al. designed a dynamic covalent hydrogel in which hyaluronic acid was modified with either hydrazide or aldehyde groups and mixed to form hydrogels containing a dynamic hydrazone bond.^445^ This material was able to be injected and quickly self-heal. These traits made this material compatible with 3D bioprinting techniques, where it was demonstrated that cells could be encapsulated in the hydrogel and printed to form various shapes. However, due to the slower bond-exchange kinetics of most dynamic covalent chemistries, these networks can require much more force to induce shear-thinning. This mechanical requirement can complicate the clinical translation of these materials, since these materials can be much more challenging to inject than many of the physical hydrogels we have discussed. Recent efforts to make these systems easier to inject may ultimately resolve this issue. One promising approach used a biocompatible and fast-diffusing small molecule catalyst to accelerate bond exchange during injection to improve injectability. After injection, the catalyst quickly diffuses out from the hydrogel, leading to slower bond rearrangement and a more robust hydrogel *in situ*.^97^

Overall, the ability for dynamic hydrogels to protect cells throughout encapsulation and delivery, plus their ability to spontaneously resolidify after injection, makes these systems especially compelling for translational development of cellular delivery and tissue regeneration. Further insights into how a dynamically cross-linked matrix influences cellular behavior within the gel, or infiltration of endogenous cells, may reveal other unique capabilities that could be leveraged for these applications.

### 4.6 Applications of Hydrogel Cellular Therapies

In the following sections we summarize the specific areas where hydrogels are accelerating cellular therapies. As discussed above, hydrogels can provide significant benefits for protecting therapeutic cells during storage, delivery, and postdelivery to achieve impressive outcomes. In many instances, hydrogels themselves provide a significant benefit for engaging with the endogenous tissues and cells, and we will discuss several acellular hydrogels scaffolds that promote remarkable outcomes without the aid of therapeutic cells. Nevertheless, we will also focus on hydrogel delivery of therapeutic cells to regenerate the tissue damage caused by conditions such as myocardial infarction, neurodegeneration, and osteochondral defects.^407,408^ We also cover emerging efforts to use hydrogel carriers to improve adoptive cell therapies such as CAR T cells and autologous DCs. In many of these instances, we will see hydrogels cleverly deployed to localize therapeutic effects of helpful cells, often by promoting their proliferation and sustaining their therapeutic functionality (e.g., differentiation into lost cell types, secretion of bioactive factors, or ability to remodel their environment). Put together, thoughtfully designed scaffolds matched to potent cellular therapies are likely to have a significant clinical impact.

#### 4.6.1 Engineering and Characterizing an Immunomodulatory Niche

**Figure 29:**
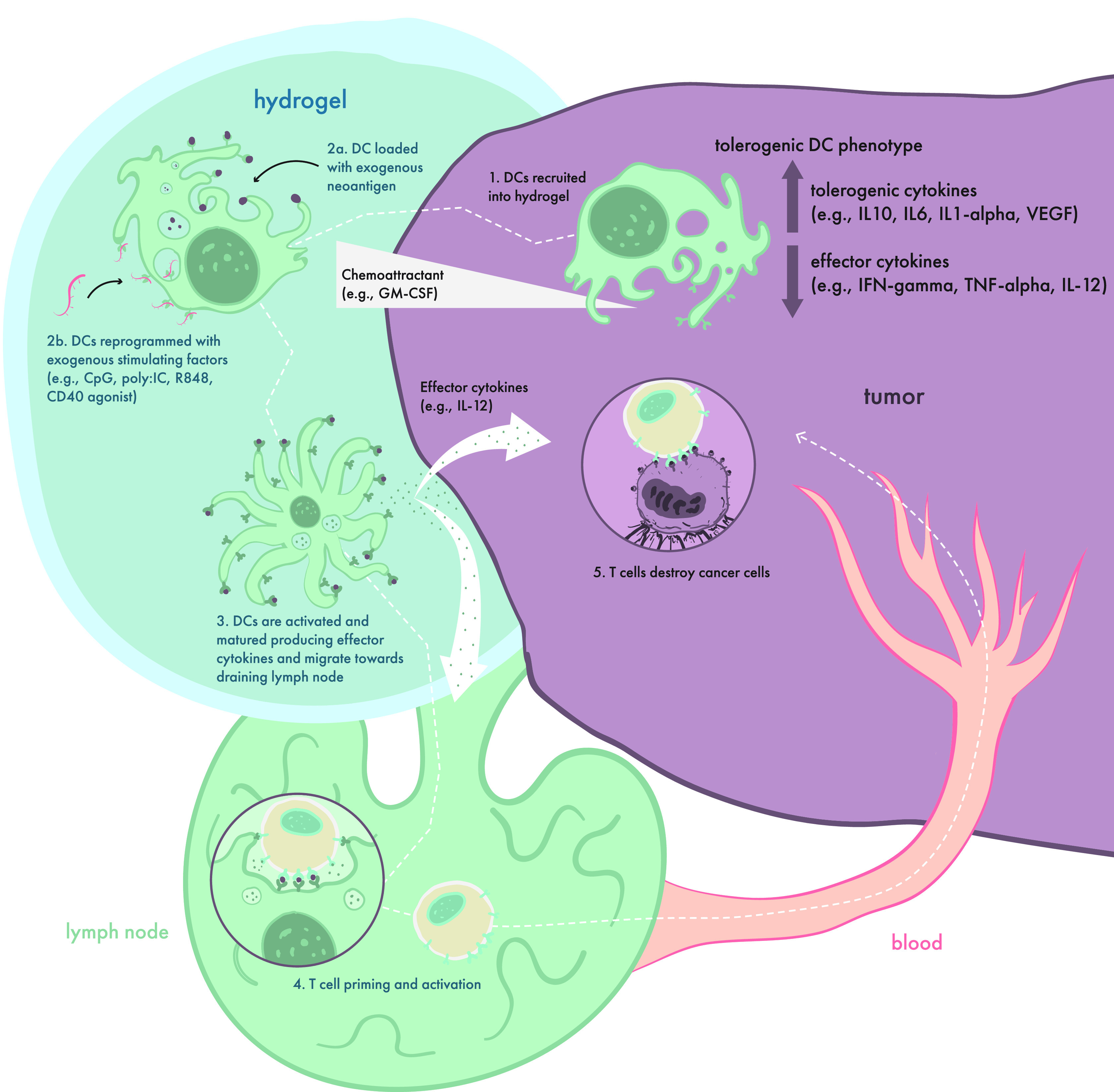
Hydrogels establish an immunogenic niche by recruiting specific immune cells, reprograming them, and then releasing them to carry out a biomedical function. Here we illustrate how a hydrogel can create an environment that can host immune cells and reprogram them to carry out antitumor functions. Dendritic cells (DCs) can be recruited to the hydrogel through the release of exogenous chemokines, like GM-CSF. Once inside the hydrogel, DCs can engage with cargo, such as exogenous tumor antigen or immuno-stimulatory adjuvants. Loaded with antigen and matured by the right adjuvant, DCs are activated and can migrate to the nearest lymph node, where they can initiate a new immune response against the tumor. Original illustration inspired by the work of Mooney and co-workers.^376^ While this demonstrates how hydrogels can create an immunostimulatory niche, similar techniques can be used to establish tolerogenic niches useful for applications such as treatment of autoimmune disease or reducing rejection in organ transplantation.

As discussed in the drug delivery section, hydrogel vaccines are highly promising, and there is growing evidence that they can generate safer and more effective results than bolus administration of the same drugs. The mechanisms behind this are still being unraveled, but one significant factor appears to be the infiltration of important immune cells into the hydrogel.^307,308^ Once inside the gel, it appears that a mixture of cues from the encapsulated drug and the hydrogel itself can stimulate these cells, while also minimizing signals from outside of the gel—such as immunosuppression from a nearby tumor (Figure 29). The result is the formation of a new immune microenvironment, often referred to as an immunomodulatory niche. Although most studies so far have focused on ways to increase the immunogenicity of the hydrogel’s immune microenvironment, there is also significant clinical value in determining ways to create immunosuppressive or tolerogenic niches, for example, for tissue regeneration applications where the host immune system may attack nascent stem cells or organoids.^446,447^ While the molecular levers and inputs are not yet fully understood, engineering an optimal immunomodulatory niche appears to provide better treatment outcomes for wide ranging biomedical applications.

Mooney and co-workers provided some of the most detailed insight into the ability of a cytokine (granulocyte-macrophage colony-stimulating factor or GMCSF) to recruit a critical class of dendritic cell (DC) into their hydrogel vaccines. Notably, they found that the dose of GMCSF could be too high, preventing those DCs from migrating back to regional lymph nodes to present their newly acquired antigen. More recent work is providing new insight into how additional immunostimulatory compounds help to shape the immunomodulatory niche. For example, Song et al. recently reported that poly(I:C), an agonist of the TLR3 pathway, both recruits and activates DCs into an injectable polypeptide hydrogel vaccine.^448^ Our recent work on hydrogel vaccines also found that antigen and poly(I:C) drove influx of endogenous APCs and in particular cDC2 migratory dendritic cells.^449^ Injectable polymer–nanoparticle hydrogels were also used to successfully recruit DCs in mouse models through the direct encapsulation and sustained release of CCL21.^450^

In addition to releasing chemoattractants to promote cell infiltration into a hydrogel niche, hydrogel scaffolds can also be engineered to promote cell infiltration. Both inclusion of adhesion motifs, such as RGD, but also degradability of the matrix can promote more endogenous cell infiltration.^419,451^ For example, Lueckgen et al. observed that inclusion of peptide cross-linkers susceptible to cleavage by matrix metalloproteinases enabled drastic increases of cell infiltration into the hydrogel depot.^419^ In general, learning how to recruit specific subtypes of immune cells is very valuable and helps to map out strategies applicable toward distinct immunomodulatory applications.

Infiltrating cells can also be reprogrammed based on the contents of the hydrogel, which can help to drive the immune response toward a particular biomedical goal. For example, an *in situ* gelling mesoporous silica rod formulation was developed that promotes sustained release of inflammatory cytokines that recruit DCs. Once in the hydrogel, DCs encounter encapsulated factors that reprogram them to be more immunogenic, eliciting a strong vaccine response.^376^ This same scaffold containing microparticles with encapsulated antigen and adjuvant has also recently been used to elicit cancer vaccine responses.^452^ Along these lines, an *in situ* gelling formulation based on dextran and 4-arm PEG cross-linking was also developed that released MIP3α and recruited DCs. To reprogram those DCs, this formulation included dual-mode DNA–siRNA microparticles that strongly activated DCs with immunomodulatory siRNA and plasmid DNA antigens.^453^

In addition to DCs, immune modulation of recruited T cells has been a valuable tool for fighting autoimmune diseases or transplant rejection. A pore-forming alginate hydrogel encapsulating GM-CSF and PLG particles containing peptide antigens was designed to induce a regulatory T cell response by delivering the peptides to DCs in a noninflammatory context to improve outcomes in a nonobese diabetic mouse model of type 1 diabetes.^454^ Remarkable antigen-specific CD4+ T cell accumulation was observed in the hydrogel with a large proportion being regulatory T cells. The pancreatic islets also contained large amounts of regulatory T cells, and disease progression appeared to be slightly delayed. While these results are preliminary, they indicate a promising path forward for tolerogenic immunomodulation using materials approaches.

Another important class of cell that can be reprogrammed by hydrogels is macrophages, which can exist along a spectrum of phenotypic states. Depending on a given biomedical problem, certain phenotypes are preferrable to mediate healing or to treat a disease. For example, macrophages can exist in a protumorigenic or antitumorigenic state. Jin et al. recently reported that inclusion of calmodulin in an injectable peptide gel could help repolarize macrophages toward an antitumor state in the local environment.^455^ Similarly, Gu and co-workers leveraged the ability to repolarize macrophages and other myeloid cells with their ROS-scavenging and pH-neutralizing hydrogel vaccines.^265,360,361^

From a design perspective, this “capture then reprogram” approach is very promising, but it requires thoughtful preparation of the hydrogel so that it fits into the biological processes that are being manipulated. Researchers must consider which cells need to be recruited, the desired residence time of cells within the hydrogel, and how to provide the necessary factors needed to successfully reprogram those cells. As seen with vaccines, recruiting and reprograming antigen presenting cells is not useful if those cells later fail to migrate to nearby lymph nodes. In most cases, the functions that manipulated immune cells need to carry out are outside of the hydrogel, so eventual cellular egress is a critical mechanism to consider. Likewise, recruited cells cannot be reprogrammed if the necessary cues are missing or made available at the wrong time.

It is likely that local immune microenvironment repolarization or reprogramming will emerge as a critical factor in the development of effective immunoengineering approaches. However, characterizing this niche and determining how individual components of these therapies (e.g., the hydrogel scaffold versus the cargo) influence outcomes is not trivial. At a minimum, these studies require careful immunohistochemical analyses to probe the presence and location of distinct immune populations. But to best understand what those populations are doing, more complex techniques such as flow cytometry and CODEX are needed. But even with these techniques, it is difficult to answer the highly specific questions the field is now asking. For example, how can researchers track the location, state, and activity of an antigen presenting cell that was recruited into a hydrogel and later migrated back into lymphatic tissue? Recently, Mooney and co-workers offered an approach that may be able to provide just such a capability, using techniques which are reasonably within reach to most groups performing materials and immunological research.^456^ The approach uses hydrogels loaded with particles carrying azido-modified sugars, which are readily internalized by DCs that infiltrate the gels. DCs metabolize the sugars and ultimately present azido groups on their surface, which can react with DBCO-modified labeling agents via bio-orthogonal click chemistry. As a result, the cells which engaged with hydrogels can be specifically labeled and analyzed alongside other cells using flow cytometric techniques. By combining metabolic labeling, bio-orthogonal chemistry, and flow cytometry, this technique allows researchers to begin specifically interrogating the altered functionality of cells which engaged with immunomodulatory biomaterials. Future techniques that allow *in situ* observation and imaging of these materials-influenced immune cells may provide the field with even deeper insight into the mechanisms at play in these systems.

#### 4.6.2 Hydrogels for Adoptive Cell Therapy

In addition to tissue regeneration, injectable cell scaffolds can be used for applications in immunology and immunotherapy. Many of the principles developed for effective cellular scaffolds in regenerative medicine can be similarly applied for immune cells, but few studies with injectable materials have been pursued. As discussed above, hydrogels provide the ability to form a type of immunological niche that begins to mimic what occurs in lymphatic tissue. In addition to cells, activating drugs and signaling molecules can be added or conjugated to hydrogel materials to promote specific cellular processes. This realization has led to efforts to further engineer these depots to behave as artificial lymph nodes or APCs. To date, researchers developing hydrogels for adoptive cell therapies have focused on the delivery of dendritic cells (DCs) and T cells.

Biomaterial-assisted immune cell delivery has focused a great deal on DCs, which are integrally involved in orchestrating the humoral immune response. Antigens and adjuvants can be colocalized in hydrogels providing a rich environment for the maturation of either endogenous or exogenous DCs. For example, alginate hydrogels have been used to codeliver DCs and stimulatory chemokines to establish an inflammatory milieu *in situ* of concentrated DCs and their secreted factors *in vivo*.^457^ In this study, increased T cell infiltration was observed with increasing numbers of exogeneous DCs in the hydrogel. In another study, this approach was used to codeliver DCs and stimulatory cytokines and improved survival in a difficult-to-treat syngeneic model of melanoma.^61^

Researchers have also investigated the expansion and delivery of T cells for adoptive cell therapies, including the delivery of CAR-T cells.^458^ Due to the lengthy cell expansion timelines before treatment, focus has been on developing 3D hydrogel culture systems that speed up T cell expansion prior to treatment. This has led to interest in hydrogels or self-assembled scaffolds that can mimic what occurs in lymphatic tissue. This kind of biomimicry requires carefully engineered surface chemistries to mediate complex biological signaling. For example, the surface chemistry of a silica microrod scaffold has a profound effect on cellular behavior, with the scaffold promoting or dampening inflammatory responses depending on if it was coated with PEG or integrins.^459^ In a follow up study, Cheung et al. leveraged this observation to engineer the surface of the MSRs toward a biomimetic APC-like surface.^460^ By coating MSRs in a lipid bilayer functionalized with cytokines and antibody agonists (IL-2, anti-CD3 and anti-CD28), this platform was able to much more efficiently engage and prime effector T cells *ex vivo*—indicating potential for use in bioreactors for adoptive cell therapies. Similarly, hyaluronic acid hydrogels cross-linked with polyethylene-glycol diacrylate were engineered with conjugated anti-CD28 and anti-CD3 antibodies to rapidly expand T cells in 3D.^461^ Overall, these studies indicate that 3D culture platforms could drastically reduce the time and space needed for the *ex vivo* expansion needed for adoptive cell therapies.

Hydrogel carriers are also providing new insights into the mechanobiology of T cells, which may shed light on critical cues relevant to *in vivo* function as well as *ex vivo* cell expansion. For example, a recent study by Majedi et al. reported that the stiffness of an alginate hydrogel had a dramatic effect on T cell motility and degree of activation, even when the porosity of the materials is held constant.^462^ This study found that stiff gels (∼44 kPa) could significantly improve T cell activation compared to soft gels (∼4 kPa). T cells show enhanced proliferation and activation, measured by the release of cytokines, and expression of surface activation markers (CD25). Similar effects have been observed in 2D,^463^ but these 3D studies imply stiffer hydrogels may lead to improved *ex vivo* expansion, which currently creates a significant lag time between patient cell acquisition and subsequent treatment. These studies may also provide insight on the design of T cell delivery gels that are effective at maintaining T cell activity *in vivo*, which could improve outcomes for patients receiving adoptive cell therapy.

**Figure 30:**
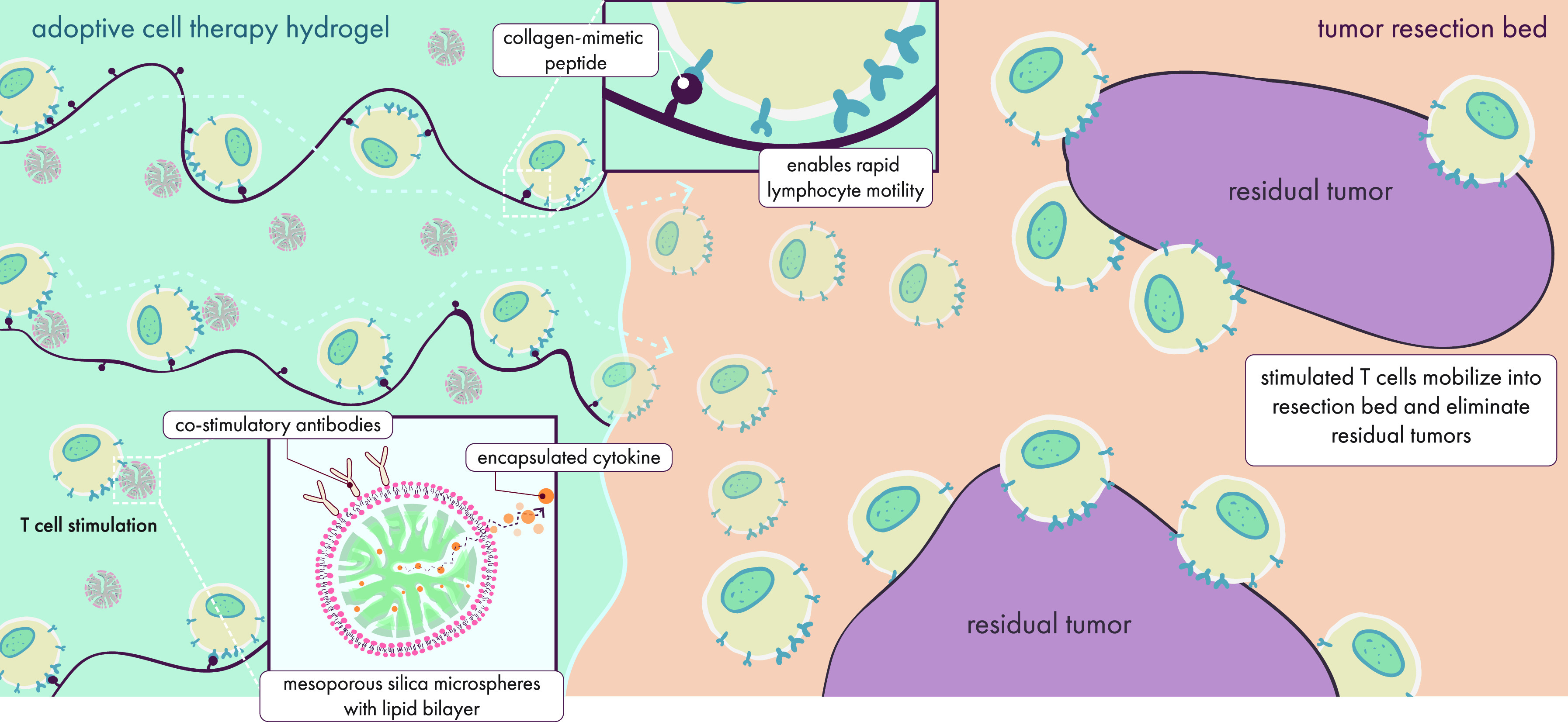
An army in a hydrogel. Adoptive cell therapy with hydrogels can overcome several challenges, such as delivery of T cells to cancerous tissue and maintaining their activity. Researchers have developed hydrogels functionalized with collagen mimetic peptide along the gel matrix, which facilitates rapid motility of encapsulated T cells through the medium. By coencapsulating T cells with microspheres that simultaneously display costimulatory antibodies and sustain the release of effector cytokines, this platform also performs as an artificial antigen presenting cell, thereby maintaining T cell activity. Original illustration inspired by the work of Stephan and co-workers.^23^

The Stephan group has notably developed alginate implants for the local delivery of T cells (Figure 30). These IKVAV-functionalized alginate hydrogels were embedded with microspheres functionalized with anti-CD3, anti-CD28, and anti-CD137 antibodies and loaded with IL-15 cytokine. When the T-cell-seeded alginate implant was placed at the tumor site, remarkable efficacy and T cell expansion was observed in treating tumor resection and inoperable tumor mouse models.^23^ In a follow-up, STING agonists were delivered in the alginate implants along with adoptive T cells.^464^ This codelivery method enabled eradication of tumor cells that did not express the T-cell-targeted antigen, eliciting global tumor immunity and treatment of heterogeneous tumors. In a study by Figdor and co-workers, injectable RGD-functionalized polyisocyanopeptide (PIC) hydrogels were used to both expand and deliver T cells. Interestingly, this hydrogel material was found to elute T cells to the organs and blood similarly to bolus controls.^465^ It is important to note that the goals of these materials are different from the delivery of regenerative stem cells, where it is often beneficial for the exogenous cell to remain in the scaffold. For T cell delivery, these hydrogels need to be able to facilitate quick egress from the gels before the cells become nutrient deficient (which is exacerbated by the high cell densities administered in these therapies). From the research thus far, inclusion of adhesion motifs may be a critical design component to facilitate this type of rapid motility.

Injectable materials could be developed to be easily administered at tumor sites without surgery, where they can release cells for local treatments. An outstanding question is whether local adoptive cell therapy may potentially reduce immune related toxicities, which have led to fatal complications in the clinic. These approaches may also improve biodistribution of therapeutic T cells to target tissues, as a high proportion of T cells localize to the lungs and spleen with current intravenous methods of delivery. This could be especially impactful for the treatment of solid tumors, which currently fail to respond to CAR T therapies in part due to poor penetration into tumors.^466,467^ Regional delivery of CAR T cells as a bolus is already indicating this kind of benefit,^468^ and further benefits may be possible with hydrogels engineered to tune the CAR T cell responses with specific codelivered stimulatory and signaling molecules. For example, hydrogels could be engineered to decrease CAR T exhaustion for prolonged and stronger treatment responses, potentially with fewer cells than is currently possible. As hydrogels for adoptive cell therapy continue to be explored, we may soon see efforts to deliver other cell types, such as NK cells.

#### 4.6.3 Stem Cell Mediated Tissue Regeneration

Stem cells are a major cell type used in regenerative medicine applications due to their ability to differentiate into many distinct tissue lineages. Hydrogels can be powerful tools for controlling stem cell differentiation by providing specific mechanical and chemical signaling cues.^409^*In vitro* 3D cell culture studies have provided compelling evidence that hydrogels’ mechanical and chemical cues have a powerful effect on the fate of encapsulated stem cells.^469,470^ For example, 3D culture in hydrogels reveals the strong effect that rheological properties (e.g., stiffness and stress relaxation) have on stem cell differentiation.^469^ More specifically, compliant materials generally promote soft tissue lineage differentiation (neural and fat cells), while stiffer materials lead to hard tissue lineage differentiation (bone cells).^471^

The most commonly used stem cells in the clinic are pluripotent stem cells, such as induced pluripotent stem cells (iPSCs) or mesenchymal stem cells (MSCs).^472^ These cells can differentiate into many cell types including osteogenic, adipogenic, chondrogenic, and neural cell types, making them highly attractive for widespread applications. Many fundamental materials-focused cell delivery studies have used pluripotent stem cells as their model cell type due to their widespread applicability.^69^ Because stem cells require engagement of adhesion peptides to survive, many engineered hydrogels include natural materials, such as gelatin, that promote cell adhesion and attachment. Clever chemistries have been used to induce controlled gelation of gelatin-based materials such as the design of a gelatin-hydroxyphenylpropionic acid-based gel, which is cross-linked by hydrogen peroxide and horseradish peroxidase.^430^ This hydrogel induced stiffness-dependent differentiation of hMSCs toward neuronal cell fates. While we have a detailed understanding of *in vitro* culture conditions for differentiation, efforts to leverage these observations for *in vivo* studies face the added complexity of interfacing with the body’s own mechanical and chemical environments, as well as an often unfriendly response from the immune system. Nevertheless, *in vivo* validation of these fundamental studies is needed in order to further advance translation of stem cell therapies.

#### 4.6.4 Paracrine Signaling from Cellularized Hydrogels

In addition to local regrowth, encapsulated cells can send paracrine signals that trigger biological processes and recruit other cells to their location or activate further regenerative processes. From this perspective, transplanted cells can act like local living “drug machines”. Hydrogels can be used to control the diffusivity and release rate of these signals and thus spatially shape the strength of these signaling processes. For example, this approach has been particularly effective in engineering the bone marrow niche. Collagen hydrogels were used to investigate the effects of autocrine vs paracrine cues on hematopoietic stem cell (HSC) fate transitions.^473^ These studies selectively investigated the effects of matrix diffusivity and niche cell coculture with inhibitory cocktails of autocrine or paracrine signals, demonstrating the importance of hydrogel design.^473^ Another study used methacrylated gelatin hydrogels to understand the effects of diffusion-regulated paracrine signals from MSCs to HSCs for engineering the bone marrow niche.^474^ MSCs in particular are known and used for their abundant paracrine signaling,^472^ and biomaterials must be designed carefully in order to preserve this critical function. For example, *in vitro* studies have found that MSCs encapsulated in hydrogels with an average pore size of 125 μm are more susceptible to triggered paracrine signaling than MSCs encapsulated in gels with pores averaging 10 nm.^475^ Future studies ought to evaluate the effect of paracrine signals from hydrogels *in vivo*, in particular the duration of *in vivo* paracrine signaling and its capacity to orchestrate the desired responses from endogenous tissues. For example, it would be valuable to determine if paracrine signaling from MSCs could reprogram or alter the foreign body response to implanted biomaterials and provide a novel way to engineer material–host interfaces.

#### 4.6.5 Traumatic Wound Healing

One of the most attractive applications of regenerative medicine is wound healing, which involves complex cascading responses from many cell types. If healing does not proceed optimally, this can result in damaged or scarred tissues. Therefore, technologies that can stimulate the right responses at the right time and in the right place are quite valuable.^476−478^ Along these lines, hydrogels not only act as release mechanisms of regenerative factors but also act as scaffolds for infiltrating restorative cells, providing both chemical and mechanical cues with spatiotemporal control.

Given the complexity of the wound healing process, there have been numerous strategies to evoke improved healing outcomes with hydrogels. In one study, Ma and co-workers developed a series of injectable, adhesive, and conductive hydrogels based on quaternized chitosan-*g*-polyaniline (QCSP) and benzaldehyde group-functionalized poly(ethylene glycol)-*co*-poly(glycerol sebacate) (PEGS-FA), and they found that these hydrogels were effective antibacterial and electroactive dressings for cutaneous wound healing *in vivo.*(479) By optimizing the cross-linker concentration, the authors found remarkably improved blood clotting and wound healing in a full thickness skin defect model, which corresponded with upregulation of local growth factors.

Injectable peptide amphiphile nanofiber materials have also demonstrated the ability to control hemorrhages.^480^ This material was designed to bind to tissue factors as a way to treat noncompressible torso hemorrhage. Notably, this study presented an interesting strategy; it used gels as a means to interface with and manipulate endogenous tissue factors *in situ,* circumventing the need to load exogenous factors. In another study, using recombinant sequence design, a set of partially ordered polypeptides (POPs) was developed that demonstrated unique thermal hysteresis and the ability to form viscoelastic networks above threshold temperatures.^481^ In a fascinating time series experiment, cellular infiltration was investigated *in vivo*. The analysis of the recruited cells indicated that the POP depots undergo a wound healing response with an initial, mild inflammatory phase that resolves over time, followed by angiogenesis and proliferation of nonimmune cells.

#### 4.6.6 Bone and Cartilage Repair

Bone and cartilage engineering aims to improve the quality of life of patients suffering a wide range of issues that include congenital defects, traumatic injury, and age-related degeneration. Since bone is a highly stiff material, the hydrogels used in this application area tend to be quite stiff as well. For injectable systems, hydrogels that are a liquid during injection but demonstrate triggered *in situ* covalent gelation and a final high elastic moduli have been most successful.^429^ Stiffer hydrogels have been found to yield higher cell retention of exogenously delivered cells and to induce differentiation of MSCs toward osteogenic pathways.^482^ Bone also presents a highly unique growth factor and mineral composition, and hydrogels containing compatible chemical signals such as calcium phosphate or bone morphogenic proteins have shown enhanced osteogenic differentiation and biointegration. Similarly, when phosphate groups were conjugated to a PNIPAM-based hydrogel, delivery of MSCs was improved in a rat cranial defect model, showing enhanced osteogenic differentiation, biomineralization, and host integration.^483^ Hydroxyapetite has also been incorporated in hydrogel materials with cells to mimic the bone’s structure and demonstrated improved osteogenic differentiation.^484^ From these studies, it would seem that for bone regeneration it is beneficial to engineer hydrogels to closely match the mechanical and mineral composition of bone.

Cartilage is the connective tissue that covers bones and joints, and it often encounters high-friction environments within the body, like the knee. From a clinical perspective, cartilage loss remains the primary reason for disability among adults and is an enduring biomedical challenge.^485^ Stem cells have potential for regenerating lost cartilage given their potent ability to expand and undergo chondrogenesis, but since cartilage defects are often irregular shapes with slippery interfaces, cells alone quickly disperse from the injection site.^486^ Many studies have focused on *in situ* and chemically cross-linked hydrogels that can withstand frequent agitation^435,487^ and involve delivery of MSCs, ASCs, and chondrocytes to replace lost cells. In particular, thermoresponsive hydrogels involving PNIPAM and Pluronic have been effective in enhancing cell delivery to these tissues.^431,488,489^ In another exogenously triggered approach, Evseenko and co-workers used light-triggered gelation of methacrylated chitosan-based materials to codeliver growth factors and chondroitin sulfate, which improved chondrogenic differentiation and enhanced cartilage integration in a rat chondral defect model.^490^ Although there has been extensive work in this area, most studies have focused on *in vitro* demonstrations of improved chondrocyte differentiation with few examples of improved outcomes *in vivo*.^430^ Future work that specifically evaluates the function of these materials *in vivo* will provide valuable insight into the clinical impact of these interventions.

Cell delivery appears to provide consistent benefits in cartilage regeneration, but inclusion of additional growth factors may be the way to further enhance outcomes. In one study by Stupp and coworkers, a material based on peptide amphiphiles was modified with RGD to promote cell adhesion of encapsulated MSCs. The amphiphiles were also modified with affinity sites for TGFB-1, a helpful growth factor that promotes the differentiation of MSCs into chrondrocytes. This material supported the 3D chondrogenic differentiation of MSCs *in vitro*. When injected *in vivo*, the material promoted regeneration in a full thickness chondral defect treated with microfracture in a rabbit model.^290^ Surprisingly, the strength of the effect was the same with or without exogenous TGFB1. The ability for hydrogels functionalized with the TGFB1 affinity site, but not loaded with the exogenous growth factor, to mediate the same efficacy as gels loaded with exogenous TGFB1 is a very intriguing result. It implies either that the growth factor is unnecessary or that simply adding affinity sites allowed the hydrogel to adequately concentrate this factor from the endogenous pool of TGFB1. Further studies into this effect would provide valuable information with major translational implications for these scaffolds.

#### 4.6.7 Cardiovascular Regeneration

Heart disease remains the leading cause of death in the United States, with coronary heart disease currently causing 1 in every 7 deaths. With such a pressing need, vascular tissue regeneration has been an area of intense research and where hydrogels have contributed to significant clinical advances. We will briefly review key examples here, but for a thorough and in-depth discussion we recommend the following reviews.^407,491,492^ Myocardial infarction (MI), more colloquially known as a heart attack, has been the principle focus for cardiovascular regenerative materials. MI occurs when insufficient blood supply leads to damaged tissues in the heart that pose significant long-term risk of death. Hydrogels acting as supportive scaffold materials have proven highly effective in decreasing the size of the infarcted area, reducing scarring, and promoting angiogenesis. In a pioneering study, the Christman group developed a myocardial ECM-based biomaterial that would gel upon injection to prevent scar formation after MI.^493^ In particular, this material prevented post-MI negative left ventricular remodeling by enhancing systolic function and contractility. The ECM-based material appeared to promote muscle growth and blood vessel formation in the infarcted areas, compared to the thin and fibrotic controls in large animal models. Subsequent work has revealed that the mechanical properties of these scaffolds play an important role in cardiovascular regeneration. For example, a hyaluronic acid methacrylate-based hydrogel was used to investigate the effect of biological and mechanical support from hydrogels as treatment in ovine MI models.^494^ Hydrogels with higher moduli showed significant improvement and decreasing infarct size compared to controls.

Growth factors such as VEGF are also powerful promoters of angiogenesis in damaged heart tissue, which can improve outcomes. Along these lines, Li and co-workers reported that conjugating VEGF directly to an aliphatic polyester gel material was more effective than including free VEGF for promoting angiogenesis.^495^ Heparin-presenting peptide amphiphiles that gel upon injection have also been used to load paracrine factors from incubation with stem cells and then release these paracrine signals upon injection in a chronic rat ischemic hind limb model causing extensive limb revascularization.^496^

Delivery of cells can help to further regenerate damaged myocardium in MI and vascular endothelium in peripheral artery disease (PAD). Many studies have shown that the delivery of hMSCs, epithelial cells, or adipose derived stem cells after MI and PAD can improve cardiovascular regeneration. Along these lines, several studies have confirmed that alginate- and calcium-based hydrogels are effective in promoting cell retention and improved impulse conduction in murine MI models and ischemic tissue models.^497,498^ Similarly, another study delivered MSCs using a thermosensitive hydrogel formulation and found that this delivery method reduced fibrous scarring and enhanced angiogenesis after MI.^499^ Consistent with results from other cellular hydrogel therapies, studies have found that incorporation of RGD into alginate scaffolds can drastically improve acute retention of cells in cardiovascular tissue.^500^ Likewise, engineered codelivery of growth factors, such as VEGF or FGF, has been found to further promote cellular engraftment and growth and yield increased vasculogenesis in damaged tissues.^501^

Going forward, the administration methods that are the most viable in the clinic ought to be considered in the design of novel regenerative hydrogel formulations. For example, catheters allow for a much less invasive delivery of cells to the heart, so moving forward, dynamically cross-linked shear-thinning gels may be more suitable for this application compared to triggered gelation methods (e.g., temperature-triggered gelation) to reduce the risk of premature gelation and clogging.^502^ However, it is worth noting that the properties required for injection through a catheter are quite different from injection from a syringe, as discussed in section 2. Future studies may benefit from extensive rheological characterization of candidate materials to identify those capable of this translationally relevant administration method.

#### 4.6.8 Regenerating the Nervous System

Injury or disease of the spinal cord (SCI) and brain leads to devasting consequences to a patient’s quality of life and cognitive functioning, and interventions that can restore partial or complete function are badly needed. Several studies utilizing hydrogels as cellular scaffolds for applications in the nervous system have revealed their potential for regenerating these unique tissues. In particular, the Stupp group has done pioneering work on peptide amphiphile (PA) nanofibrous materials for neuroregeneration and shown that when these materials are modified with the cell adhesion epitope IKVAV, they can prevent scar formulation after spinal cord injury. In an *in vivo* model PA materials decreased astrogliosis, decreased cell death, and enhanced the number of oligodendroglia at the site of injury, leading to behavioral improvements.^503^ This PA IKVAV-modified hydrogel was also shown to promote plasticity of serotonergic fibers after spinal cord injury in mouse and rat models.^504^

Delivery of neural stem cells or neural progenitor cells in hydrogel materials has also been highly effective for SCI and stroke. Due to the highly sensitive nature of neural tissue and the low cell retention typically achieved during cell delivery, very small volumes of hydrogel material (<25 μL) have been used to precisely deliver several million cells at a target location intracranially or in the spine.^437^ Hyaluronic acid and methylcellulose-based materials (HAMC) have been successfully used as delivery vehicles for neural stem and progenitor cells and iPSCs in spinal cord injury models in rats with reduced scarring, inflammation, and even animal recovery.^505^ When mixed, these biopolymers form a physically cross-linked, injectable dynamic hydrogel.^76,505^ Like with other cellular therapies, it appears that inclusion of growth factors and adhesion motifs further improves the therapeutic efficacy of cell delivery. For example, growth factors including PDGF have been codelivered and conjugated to hydrogels, which has led to increased cell retention, neuronal differentiation, and decreased off-target teratoma formation. Other hydrogels containing hyaluronic acid (which can bind to surface receptors on cells) have successfully shown improved cell retention for ischemic stroke therapy.^506,507^ Recently the shear-thinning hydrogel for encapsulation and long-term delivery, or “SHIELD”, hydrogel was shown to improve Schwann cell transplantation in a cervical contusion model by 700%.^508^ This hydrogel contains a copolymer of PNIPAM and a multiarm PEG that interacts with a C7 protein containing the RGD integrin binding motif. Overall, significant strides are being made with hydrogels for neuroregeneration, and in particular the benefits of highly organized and functionalized scaffolds appear to be considerable.

#### 4.6.9 Other Applications in Regenerative Medicine

Advances in understanding cellular biology have led to novel therapies for less common diseases and applications ranging from vision loss to cosmetic defects. For example, retinal degradation has been treated through the improved delivery of retinal stem cells in the hyaluronic acid and methylcellulose (HAMC) hydrogels from the Shoichet group.^509^ Lipoaspirate was used as a natural gel material for delivering adipose derived stem cells to repair adipose tissue deficits.^510^ Additionally, muscle stem cell transplantation is improved through use liquid crystal peptide-based materials.^511^ Notably, this approach enhanced cell engraftment and improved proliferation in murine models, in theory by aligning muscle stem cells with the liquid crystals. As we will see in the clinical translation section, there are even injectable hydrogels currently being evaluated for their ability to regenerate hearing function. In many ways, it appears that the regenerative potential for hydrogels is limitless. The current breadth of applications provides strong support that these systems can be tailored to benefit virtually any tissue type in the human body. However, each tissue in the body has its own unique properties that need to be taken into account during development of a regenerative hydrogel, highlighting the critical role of collaboration with biologists and clinicians in the early design stages—particularly for materials designed to regenerate tissues where there is a lack of pre-existing literature.

## 5 Other Biomedical Applications of Hydrogels

Although drug and cell delivery are perhaps the most extensively studied applications of hydrogels in medicine, there are numerous additional and important research areas. In particular, there are important implications for hydrogels in surgical situations, both during and after procedures. In particular, hydrogels with hemostatic capabilities are proving to be quite impressive for controlling bleeding during surgery, which also has implications for treating acute trauma. Sprayable hydrogels are also capable of preventing the formation of surgical adhesions, a painful and very common complication from surgery. Hydrogels are also being explored for their ability to coat and improve the biocompatibility of a host of different medical implants and devices. These coatings are becoming increasingly multifunctional and hold significant promise for next-generation biosensors. This section is dedicated to these exciting and emerging application areas.

### 5.1 Hydrogels for Surgical Applications

Hydrogels have been investigated for the treatment and prevention of adhesions following surgical operations. Surgical adhesions, or postoperative adhesions, are fibrous bands of scar tissue that form between internal organs and their surrounding tissues as a result of natural healing processes following surgery.^512,513^ Adhesions occur in upward of 95% of patients and, each year, put more than 19 million patients at risk for adhesion-related complications in the United States alone.^514−517^ These complications place significant burden on the US healthcare system, leading to billions in treatment-related costs each year.^518^ The two most common commercial products for adhesion prevention are solely indicated for use in the abdomen and are solid, resorbable membranes composed of hyaluronic acid (HA) and carboxymethylcellulose (CMC) in the form of a film (Seprafilm, Sanofi/Genzyme) or a woven fabric (Interceed, Ethicon).^519^ In practice, these barriers are often difficult to administer over the target tissues to adequately provide surface coverage, which is necessary to prevent adhesion formation between the tissues and organs of interest. Furthermore, these sheet-like barriers have been reported to become easily dislodged on account of natural tissue movement and cannot fully cover the surface of target tissues with irregular surfaces or those that are heavily folded, such as the small intestine, leaving these surfaces vulnerable to potential adhesion formation.^520^

Numerous groups have investigated sprayable polymer solutions comprised of chitosan, HA, and/or CMC to circumvent the difficulties associated with the application of solid barriers.^521,522^ These sprayable polymer solutions undergo *in situ* polymerization to form covalent hydrogels with tunable mechanical properties, and they have been shown to increase the local residence time in the body to aid in effective adhesion prevention.^523−526^ Li et al. demonstrated reduced peritoneal adhesions following the administration of an *in situ* cross-linking hydrogel treatment comprised of N,O-carboxymethyl chitosan (NOCC) and aldehyde hyaluronic acid (A-HA). Chitosan specifically has shown excellent hemostatic properties, meaning that the material prevents and stops bleeding by promoting clot formation.^527^ The hemostatic properties of the NOCC/A-HA hydrogel, due to the inclusion of chitosan in the hydrogel composition, could be a particular advantage of this hydrogel system for preventing postsurgical bleeding, which is a potent stimulus for adhesion formation.^528^ Future studies conducted with this system ought to investigate this hypothesis regarding hemostatic properties and subsequent adhesion prevention.

*In situ* polymerization of hydrogels for adhesion prevention has been widely investigated with polyethylene glycol (PEG)-based materials and translated into human trials. Napoleone et al. evaluated the efficacy and safety of CoSeal for the prevention of pediatric cardiac adhesions. CoSeal was applied via a “product-specific gas-driven spray device” that covered the visible surface area of the heart and great vessels in 76 pediatric cardiac surgery cases, prior to sternal closure.^529^ The results of this study reported consistently low adhesion classifications with 85% of adhesions categorized as “filmy and avascular”.^529^ However, the study design was observational and lacked an appropriate control arm. Additionally, 6 adverse events were reported as potentially associated with the application of CoSeal (cardiac tamponade and cardiac fibrillation).^529^ These safety concerns were addressed through protocol amendments and improvements in the CoSeal application technique. While further controlled studies with CoSeal are warranted given the overall positive safety profile and observed adhesion reduction, the authors comment on FDA concerns regarding study design and measurable end points that could introduce translational challenges in future development. Later, Banasiewicz et al. investigated a SprayShield adhesion barrier system composed of a PEG ester amine solution and a buffer solution that undergo rapid *in situ* polymerization upon mixing. A total of 30 subjects underwent restorative proctocolectomy with ileal J-pouch-anal anastomosis and were randomized to receive SprayShield via an air-assisted sprayer or no treatment at the end of the operation prior to closing.^530^ While adhesions occurred in 37.5% of subjects treated with SprayShield with an average adhesion severity score of 0.9 compared to 66.7% of subjects with an average adhesion severity score of 1.3 in the control group, SprayShield did not demonstrate a significant reduction in adhesion formation due to the small number of subjects enrolled in the trial.^530^ While participating investigators reported that SprayShield was easy to use and the observed safety outcomes suggested no association between adverse events and the investigational adhesion barrier, a larger clinical study, statistically powered to detect differences of clinical relevance is needed to better assess the safety profile and potential efficacy of SprayShield.

Tissue adherence capabilities are another adhesion barrier design parameter that appears to be an important consideration for these types of surgical interventions. Yang et al. demonstrated the benefits of a tissue-adhesive hydrogel composed of o-nitrobenzyl alcohol (NB), modified carboxymethyl cellulose (CMC-NB), and glycol chitosan (GC) that underwent a photoinduced imine-cross-linking reaction to form a hydrogel adhesion barrier (CNG hydrogel). The aldehyde groups generated from the CMC-NB react with the amino groups distributed on GC or tissue surfaces to form a hydrogel adhesion barrier that covalently attaches to the tissue.^531^ In this work, the CNG hydrogel was compared to a previously studied hydrogel composed of hydroxybutyl chitosan (HBC) that weakly adheres to tissue via a noncovalent, physical attachment.^532^ The administration of the CNG hydrogel was completed within 5 min utilizing a syringe for the initial material deposition and light irradiation to cross-link the hydrogel. The published results indicate the CNG tissue-adhesive hydrogels were better at preventing inter- and intratissue postsurgical adhesions. In contrast, the poorly tissue-adhesive HBC group saw inconsistent levels of postsurgical adhesions, suggesting that consistency of the outcome could depend on hydrogel-tissue adhesion strength. This variability may be due to the potential slippage and dislodgement of the HBC hydrogel from the site of application as a result of weak tissue adherence, which would lead to more exposed tissues than seen with CNG hydrogels.

While tissue adhesive properties that allow for covalent attachment of hydrogel to tissue can prevent hydrogels from becoming dislodged from the site of application, these materials can still fracture similarly to commercially available sheetlike barriers resulting in poor adhesion prevention.^530^ Additionally, other potential side-effects of *in situ* polymerization include cross-linking of the native tissues, resulting in greater adhesion formation due to the nonbioorthogonal nature of the chemistries used for hydrogel cross-linking.^530^ In these cases, the material can tightly adhere two tissues together and create similar problems associated with postoperative adhesions. Finally, covalent hydrogel materials are able to swell significantly, reaching upward of 400% volumetric expansion. This type of expansion can be severely problematic when using materials for thoracic or cardiac surgeries where expansion can cause cardiac tamponade or mechanical compression of the heart.^529,533^

**Figure 31:**
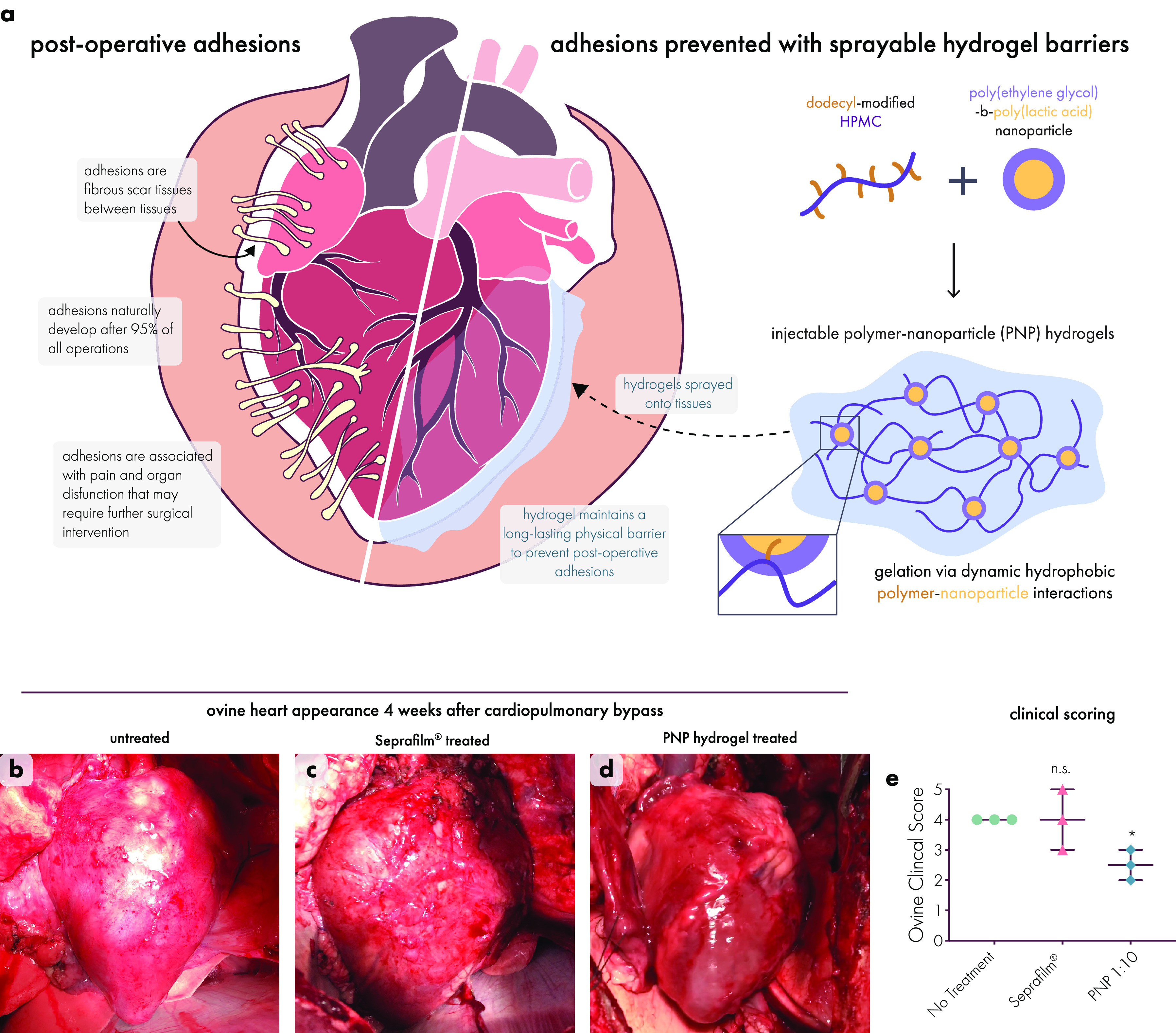
(a) Schematic representation of postoperative adhesions, fibrous bands of scar tissue that form between internal organs and tissues. Dynamically cross-linked polymer–nanoparticle (PNP) hydrogels can be applied between organs and tissues, preventing adhesion formation by maintaining lubricity between tissues and allowing internal structures to move naturally. (b) Representative image of a dissected untreated ovine heart following a cardiopulmonary bypass operation. (c) Representative image of a dissected Seprafilm-treated ovine heart following a cardiopulmonary bypass operation. (d) Representative image of a dissected PNP 1:10 hydrogel-treated ovine heart following a cardiopulmonary bypass operation. (e) Blinded clinical scoring of adhesion formation for each treatment group 4 weeks following cardiopulmonary bypass. Data presented as mean ± s.d. (*n* = 3). Original illustration inspired by the work of Stapleton et al.^41^ and photographs reproduced with permission from ref (41). Copyright 2019.

More recently, our group improved upon the limitations associated with covalent hydrogel systems and investigated the use of a noncovalent, transiently cross-linked dynamic hydrogel platform to prevent surgical adhesions (Figure 31). In this study, a polymer nanoparticle (PNP) hydrogel resulted in a 85% and 38% reduction in adhesion formation in both rodent and ovine models of surgical adhesions, respectively.^41^ The PNP hydrogel system did not swell, was easily administered via spraying, persisted at the site of interest for at least 2 weeks, and exhibited dynamic mechanical properties allowing for natural tissue movement.^41^ Additionally, the reported system addresses other translational hurdles, such as scalability due to facile manufacturing requirements and clinical adoption due to the simple route of administration. Further studies exploring additional surgical indications, such as abdominal or pelvic surgery, would broaden the translational potential of this technology.

Hydrogels also provide a unique opportunity for a combinatorial approach to postoperative adhesion prevention. Similar to the adhesion barrier composed of chitosan that provides hemostatic properties while simultaneously providing a physical adhesion barrier to the tissues of interest, these materials can be engineered to provide a multifunctional approach to adhesion prevention. Recently, small molecular inhibitors of hypoxia inducible factor 1 alpha (HIF1a) resulted in significant prevention of adhesion formation in mice undergoing peritoneal adhesion induction.^534^ While these small molecule inhibitors demonstrated promising results, these treatments were administered via repeated dosing to achieve efficacy. Loading these small molecule inhibitors into hydrogel adhesion barriers as therapeutic cargo could be a transformative approach to adhesion prevention by simultaneously addressing the physiologic formation of adhesions while maintaining a physical barrier between the tissues of interest.

Hydrogels have also demonstrated promising results as hemostatic agents and surgical sealants. Uncontrollable or excessive bleeding following trauma or during surgery is a major cause of global morbidity.^535^ For example, repair of aortic rupture and cardiac bleeding following cardiac injury or penetration wounds are critical clinical challenges.^536^ Surgical operations may also require the ability to seal and/or connect tissues together or stably incorporate implantable devices into native tissues.^537^ Currently, sutures and staples are the most widely used clinical method for both restoring hemostasis and/or reconnecting tissues during operations.^538^ Not only are these methods not feasible in emergency situations outside of surgical units, these techniques are challenging and time-consuming. In general, these methods can increase the risk of infection, do not provide an immediate, leak-free seal, and are difficult to deploy during minimally invasive procedures where certain regions of the body are not readily accessible.^536,539^ Additionally, piercing tissues with sutures and/or staples can cause further tissue damage, especially fragile, previously damaged tissue.^540,541^ While the use of current hemostatic agents can reduce blood loss and increase survival rates, these agents are still associated with poor adhesion strength to wet tissue, toxicity, inadequate gelation times, and inflexible bonding mechanics.^542^ Likewise, surgical sealants have been demonstrated to more effectively seal wounds than suture alone, reducing patient blood loss and risk of infection.^543^ However, these sealants also suffer from overall weak tissue adhesion and the inability to adequately reconnect tissues in dynamic environments, such as areas that experience variable contraction and blood flow.^544,545^

**Figure 32:**
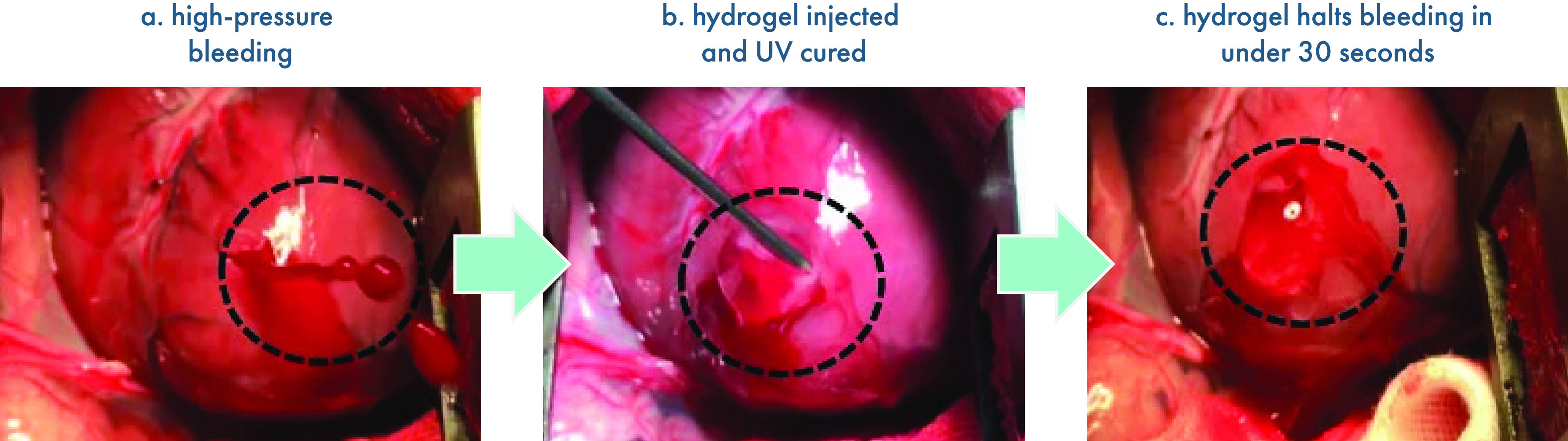
(a) Gross view of a representative ventriculus sinister puncture in a pig heart via a 6-mm (inner diameter) needle, which causes immediate high-pressure bleeding. (b) Gross view of the injected hydrogel administered to cover the punctured cavity and rapidly cure following UV irradiation. (c) Gross view of the cessation of bleeding (within 30 s) from the ventriculus sinister puncture and coverage of the punctured cavity as a result of hydrogel administration. Adapted with permission from the work of Ouyang and co-workers.^552^ Copyright under Creative Commons CC BY 2019.

Recent advances with *in situ* polymerizable hydrogels for hemostatic agents or surgical sealants have generated exciting results that address many of these limitations, including enhanced tissue adhesion properties, biocompatibility, favorable mechanical properties, and faster, controlled gelation times.^546−550^ For example, *in situ* polymerizable hydrogel systems can conform to the complex geometries of traumatic wounds or folded tissues, thereby improving surface coverage. Specifically, Annabi et al. developed a methacryloyl-substituted tropoelastin (MeTro) elastic hydrogel surgical sealant with biocompatible and tunable adhesive properties. This study demonstrated that the MeTro elastic hydrogel can not only effectively seal blood vessels and lung tissue in small animal models of tissue injury but also effectively seal and prevent leakage in a preclinical, large animal porcine model of lung leakage.^551^ The MeTro system relies on UV light for cross-linking to control polymerization and application of the material, preventing uncontrolled, rapid polymerization. While these results are promising, future studies exploring the degradation profile of this material and the long-term effects on wound healing would further enhance the translational potential of this platform. For hemostatics, Hong et al. reported a biomimetic hemostatic agent that polymerizes and strongly adheres to wet tissue within seconds after UV photoactivation. Remarkably, this hydrogel stopped high-pressure bleeding from pig carotid arteries with 4∼5-mm-long incision wounds and from pig hearts with 6-mm-diameter cardiac penetration holes within 20 s, without the need of suture (Figure 32).^552^

These studies yielded exciting results for hemostatic materials, and future studies should work on resolving key translational challenges for these materials. In particular, reliance on *in situ* polymerization poses some difficulties for translation that will eventually need to be addressed. For example, with light-triggered systems, reliance on UV light places limitations as to where these technologies can be deployed. Beyond being mutagenic, UV light does not penetrate deeply into tissues and requires that the wound be physically accessible to the light source, which may not be feasible for internal or deep wounds. Overall, the development of materials with alternative light triggers (e.g., near-infrared) would further enhance the translational potential hemostatic hydrogels for minimally invasive surgeries or emergency trauma where access to UV light is not possible. In addition, engineering *in situ* polymerizable materials to adequately cross-link in clinically relevant timespans will be important so that materials stick where they are placed and do not prematurely gel in the applicator device. Polymerization techniques also need to be evaluated for potential off-target effects. Uniformity of cross-linking in the deposited film may also be important to optimize, particularly in spray-mix systems where two reactive components are aerosolized simultaneously. Alongside the continued development of *in situ* polymerizable systems, it may be useful to also explore the rheological advantages of dynamic hydrogels in the development of these types of hemostatic materials.

### 5.2 Hydrogel Coatings for Medical Devices

Hydrogels can be engineered to be antimicrobial and/or antifouling and can be used as coatings for medical devices to act as “skin”-like scaffolds on electronic devices and sensors. Hydrogel coatings provide an interface with the human body that can improve device function and biocompatibility. The capabilities of hydrogels can be tailored to the specific application by modulating key properties, such as their morphological (e.g., porosity), chemical (e.g., reactivity and stability), and mechanical properties (e.g., flexibility, compressibility, stiffness).^20,553^ These coatings are especially useful for long-term implants because they can improve their biocompatibility, enshrouding the underlying material with a surface that is antifouling, antimicrobial, and nonimmunogenic.^554^ Overall, these capabilities make hydrogels an ideal medium for engineering what we will call the “host–device interface”, optimizing device function and improving device lifetime in the body. Here, we summarize current techniques for incorporating hydrogels into devices, summarize several key advances for using hydrogels as antimicrobial and antifouling coatings, and highlight recent advances using these hydrogels in implantable electronic devices.

#### 5.2.1 Integrating Hydrogels into Devices

Hydrogels can be readily applied onto a variety of surfaces through chemical (e.g., polymerization directly onto the surface) or physical attachment (e.g., spray coating). Importantly, many of these methods are capable of functionalizing diverse geometries and shapes. Many relevant techniques are often industrially scalabe, such as dip coating, spin coating, spraying, or doctor blading, which can be used on a number of devices and surfaces.^555,556^ For example, Xie et al. demonstrated that acrylate-based films can be applied directly on surfaces through directly brushing or spraying a film that self-generates to form a hydrogel “paint”.^557^ Along the same lines, Pan et al. applied hierarchical nanostructured hydrogels through inkjet printing and spray coating onto paper and glass.^558^

Toward longer term adhesions of coating to a device, hydrogels can be covalently attached to surfaces, which can be activated or modified to present reactive functional handles. Silicones and metals are commonly oxidized or modified with small molecules (e.g., [3-aminopropyl]triethoxysilane) to create a reactive surface through which prepolymer mixtures can then be introduced and cross-linked onto the activated surface.^559^

**Figure 33:**
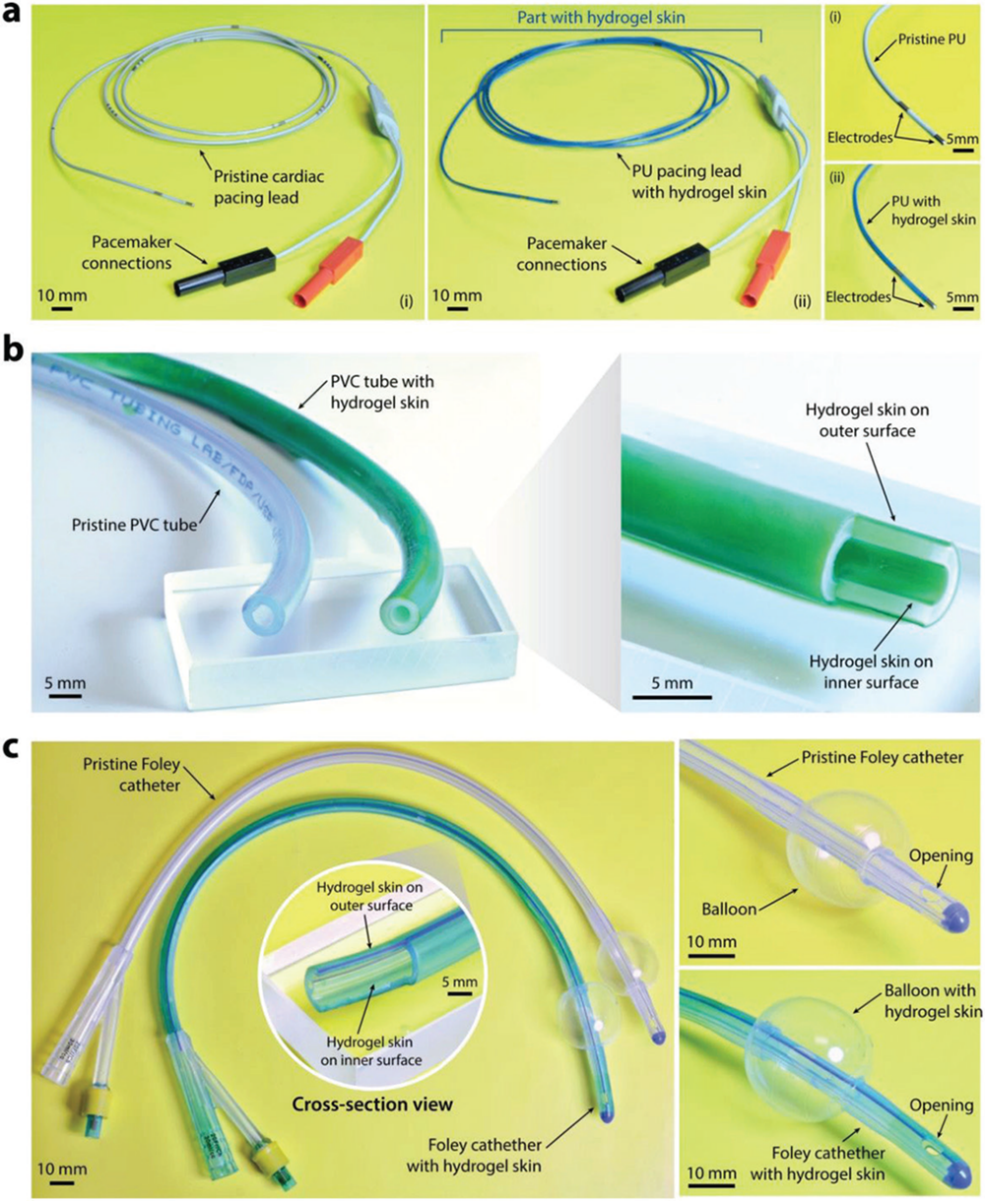
Hydrogels can be used to coat complex geometries. Hydrogel skin coatings on (a) polyurethane pacemakers (hydrogel skin in blue), (b) PVC tubing (hydrogel skin in green), and (c) Foley catheters (hydrogel skin in green). Reproduced with permission from Zhao and co-workers.^560^ Copyright 2016.

Development of “hydrogel skins” has enabled coatings onto complex geometries and architectures (Figure 33),^560^ with applications for polymeric devices of arbitrary shapes ranging from pacemaker leads to soft robots.^561,562^ This style of direct-functionalization is possible for other substrate materials, including metals. For example, Zambonin and co-workers successfully functionalized metal substrates by directly polymerizing a film on their surface using electrosynthetic techniques.^563^ This versatility indicates that hydrogel coatings are a viable option for functionalizing everything from specialty medical devices such as orthopedic implants to everyday materials such as contact lenses.

Although significant progress has been made to develop methods to incorporate hydrogel coatings on diverse materials, the lifetime and degradation of these coatings requires quite a bit of optimization. For example, the individual requirements of the implant will dictate the time scales that hydrogels need to persist in the body. Consider the needs of a glucose sensor, which are rather distinct from those of a hip joint replacement. Methods that fully and robustly characterize coating lifetime and degradation behaviors are needed to allow for clinical translation and to enhance device function. On top of these methods, strategies need to be developed to adapt or tune material properties to meet the challenges faced *in vitro*. For example, the constant mechanical forces that implants face could damage or physically degrade a coating and prevent use of the device beyond its electronic and internal capabilities. While synthetic polymers (e.g., brushes, films, or self-assembled monolayers) are widely used for bulk material casings or coatings for implantable devices, and appear robust to these forces, the mechanical integrity of synthetic and naturally derived polymeric hydrogels on surfaces is not as well understood. Adhesion to surfaces under shear forces (particularly relevant for stents and blood sensors) brings about limitations in their lifetime as well as concerns over swelling, driving a need for novel strategies to toughen hydrogels and assays to properly study degradation mechanisms and lifetime under physiologically relevant conditions. While many advancements have been made on the materials side, future work to translate these technologies relies on preclinical and clinical collaborations to precisely define standards for function, durability, and biocompatibility. These standards can then guide further engineering and characterization of these materials so that they perform clinically valuable functions and exhibit clinically relevant *in vivo* lifetimes. Toward this, durability and self-healing are properties to look out for, which can restore materials that are damaged through physical sliding or deformation due to wear and tear brought on by the patient’s everyday life.^564^

#### 5.2.2 Hydrogels as Antimicrobial Coatings

Hydrogel coatings provide novel strategies to combat one of the biggest challenges for implantable devices—infections. In 2011 alone, the United States saw nearly 185,000 cases of hospital-acquired infections associated with medical devices.^565^ Fortunately, there has been considerable work toward use of hydrogels to mitigate these device-associated infections, which arise with a wide range of devices such as catheters and ventilators. Mistakes in aseptic technique during hospital procedures and host responses from endogenous bacteria that come in contact with implanted devices lead to bacterial accumulation and biofilm formation, causing infections that are difficult to treat. In many cases, infected implants must be entirely removed and replaced, which can lead to long periods of disability and pain for patients. Even with removal, the infections often require the administration of powerful antibiotics to patients and as a result contribute to the ongoing crisis of bacterial drug resistance. Even under impeccable sterile conditions, some bacterial adhesions are unavoidable, which means that devices intrinsically pose an infection risk.

For prophylactic prevention of adherent matter or organisms, antimicrobial hydrogels may serve as “passive” coatings with inherent antimicrobial properties or moieties.^566−568^ These include hydrophilic and zwitterionic moieties that lead to strong surface hydration that can resist protein adsorption, which have benefited a number of medical devices.^569^ Cationic materials are intrinsically antimicrobial, and catheters functionalized with hydrogel coatings made from cationic polycarbonate and antifouling polymer poly(ethylene glycol) have shown resistance against Gram-positive (*S. aureus, enterococcus*) and Gram-negative (*E. coli, A. bacumanii*) bacteria and fungi (*C. albicans, C. neoformans*).^570^

As discussed previously, hydrogels can be formulated to deliver drugs using passive and active mechanisms, and hydrogel coatings can use these strategies to provide local and sustained release of antibacterial compounds to prevent implant infections. For example, the porosity of hydrogel coatings can be tuned to create “active” coatings that release small molecules or particles embedded in the scaffold. One strategy being explored clinically is the encapsulation of intrinsically antimicrobial silver nanoparticles into polyacrylamide-based hydrogels to prevent infections from *E. coli* and *S. aureus*.^571^ Another “active” antibacterial coating was developed by Schneider et al. using a bactericidal hydrogel that carried peptide MAX1, which assembled into beta-hairpins with activity against Gram-positive (*S. epidermidis, S. aureus, S. pyogenes)* and G negative (*K. pneumoniae, E. coli*) bacteria.^572^ While the mechanism of these peptide-functionalized coatings is not fully understood, these materials are thought to disrupt the bacterial membrane via electrostatic interactions with the negatively charged bacterial membrane^573^ and bacterial DNA.^574^ Strategies that combine both innately antimicrobial components, such as these peptides, and antibiotic drugs may be particularly powerful and have recently been reviewed for coating titanium implants.^575^

If hydrogel coatings can reduce the prevalence of device-associated infections, the clinical impact for patients would be considerable. Continued development of these materials against virulent bacterial strains is critical and will need to exploit all the properties hydrogels have to offer: meshes that can encapsulate drugs over long terms (at least days);^574^ mechanics that affect adhesion of the gel to the device; and the ability to respond dynamically to stimuli (e.g., temperature and inflammation). Novel properties that may further enhance these coatings include the ability to undergo wettability changes for self-cleaning^576^ and the use of mechanical vibration to displace the attachment of proteins, cells, and bacteria.^577,578^

#### 5.2.3 Hydrogels as Anti-Fouling Coatings

Although device-associated infections are a serious source for morbidity due to medical device implantation, rejection of the implant by the body is a major factor limiting both the lifetime and functionality of implants. The most common way the body rejects an implant is through the foreign body response, a complex set of molecular events that begins with fouling of the surface and ends with the formation of a fibrotic capsule as the body works to eliminate macroscopic foreign materials.^579−581^ The success of antimicrobial hydrogel coatings, which in part depends on prevention of biofilm formation, has led to research into hydrogels as antifouling coatings. Antifouling materials seek to prevent the nonspecific adhesion of molecules onto device surfaces, which occurs instantaneously when materials come in contact with complex fluids. All implanted materials are prone to fouling that can lead to thrombosis, inflammation, and occlusion. Fouling initiates with nonspecific adhesion of proteins, which undergo conformational changes upon adsorption onto the surface that reveals parts of the protein normally hidden in the native conformation. These revealed sites can then mediate interaction with parts of the immune system. For example, some proteins, such as fibrinogen, serum albumin, complement, and lysozyme, opsonize a surface, which is to say they undergo dynamic interactions and rearrangements that cause platelets and cells to agglomerate and aggregate onto the fouled surface.^66^ Immune cells such as macrophages are also sensitive to these misfolded proteins and are quickly recruited to the site and secrete inflammatory cytokines. In the context of a pathogen or parasite, this entire process is quite beneficial. But in the context of a long-term implantable device, the foreign body response leads to the eventual deposition of collagen matrices that create an isolating capsule around implants, the formation of remarkably corrosive foreign-body giant cells, and the recruitment of myo-fibroblasts that exert significant deformational compressive forces on implants.^581−584^ To prevent this formidable defense mechanism from destroying or disabling medical implants, materials approaches look to head it off at the very first step—fouling.

Perhaps our best strategy for developing antifouling materials has been the development of materials with specific interactions with water molecules, namely very strong interactions to create a protective layer of water to sterically hinder access to the underlying material.^569^ As our fundamental understanding has evolved, the field has pinpointed parameters including surface packing, hydrophilicity, electrical neutrality, and flexibility of polymer chains as contributors to these antifouling properties.^585,586^ On a mechanistic level, materials with a tight hydration layer establish a physical and energetic barrier to prevent nonspecific adhesion and increase the free energy barrier required for proteins to adhere.^569^ Because of their hydrophilicity, hydrogels in particular are great candidates for creating this barrier both with water and potentially other solvents.

Although quite different for the human body, biomedical antifouling technology may learn a great deal from efforts in the maritime industry to develop antifouling surface coatings on underwater structures, which are prone to adhesions from plants, algae, barnacles, and other seafaring organisms. These accumulations increase the drag of moving vessels and require considerable upkeep. In particular, hydrogel surfaces for antifouling marine surfaces have shown resilience against bacteria and barnacles,^587^ and these fully cross-linked networks are suspected to be environmentally benign. These studies suggest that hydrogels are promising as antifouling surfaces even in highly complex and relatively harsh environments and could provide clues for developing similar coatings for medical implants.

In the medical and biological space, hydrogels have reduced protein and microbial adhesion to devices in serum and whole blood.^588^ Toward materials development, screening of combinatorial alginate-based hydrogels containing triazole analogs identified formulations that reduce fibrosis and lead to reduced immune cell recruitment, indicating potential applications for coating devices.^589^ Notably, poly(ethylene) glycol^570,590^ and zwitterionic hydrogels, incorporating sulfobetaine, carboxybetaine, and phosphorylcholine moieties,^591^ are gold standard polymeric materials that have been successful in both biomedical and marine spaces. As a biomaterial, they have reduced platelet adhesion on devices that are in contact with blood,^592,593^ and they have reduced the foreign body response, promoted angiogenesis,^593^ and improved pharmacokinetics when coupled to proteins.^594^ As research into antifouling hydrogels continues, it will be important to determine the time scales over which they can protect devices, as well as how that time scale may change based on the area of the body they are exposed to (e.g., blood versus subcutaneous versus brain).

It is worth noting that hydrogel coatings could further improve the biocompatibility of relatively rigid devices, say in the context of a neural electrode, by providing a much more compliant interface with soft brain tissues. However, it is unclear whether a balance must be struck between the durability of the coating and efforts to match the mechanical properties of its surrounding tissues. Along these lines, efforts to decouple the antifouling capabilities of hydrogels from their mechanical properties may be valuable for developing protective and biocompatible coatings for diverse types of tissues.

#### 5.2.4 Hydrogel Integration with Implantable Electronics

Hydrogels were initially developed as passive coatings that protect a device, with little or no contribution toward the function of that device. More recently, hydrogel coatings with function-enhancing capabilities have been explored for electronic sensors, serving as a conduit between electronic sensing components and the body without disrupting device performance. As the desire for real-time monitoring of analytes *in vivo* for personalized medicine has increased, so has the desire for materials that allow for integration of implantable sensors with the human body.

These coatings stand to improve two of the most highly used medical devices, continous glucose sensors and insulin pumps, which both suffer from short lifespans that limit their clinical utility. To maintain healthy insulin levels, insulin pumps require frequent replacements of the implanted components and regular calibration via finger prick glucose sensors. Onerous maintenance, replacement, and calibration of the devices leads to high patient burden and tissue scarring, driving the desire for implanted devices that could reliably and wirelessly transmit patient data to the patient or healthcare provider directly. Current FDA-approved devices such as Eversense’s 90-day Continuous Glucose Monitoring System have used polymeric materials to encase their implantable devices, but twice a day finger-pricks are still needed to calibrate the system. Hydrogels offer a promising alternative to protect these devices and reduce the foreign body response that leads to device failure. Notably, acrylate-based hydrogels have been applied for continuous glucose sensing.^595,596^ Zwitterionic hydrogels have also demonstrated the ability to extend the lifetime of glucose biosensors over 12 days in blood due to their antifouling properties that reduce accumulation and aggregation of proteins that obstruct the sensor.^597^ PEG hydrogel is often used to interface between sensors and tissue to mitigate foreign body response,^598^ with poly(acrylamide) (PAAm)/PEG hydrogels acting as monitors of glucose themselves.^31,599^ Recently, our group described the use of copolymer hydrogels comprising *N*,*N*-diethylacrylamide and *N*-hydroxyethyl acrylamide, which were selected from a combinatorial copolymer hydrogel library of over 170 acrylamide-derived formulations that was evaluated in a highly parallelized platelet fouling assay. When used to coat electrochemical sensors, the leading polyacrylamide-derived hydrogel improved device performance and lifetime when compared to PEG-coated devices, showing significant resistance to blood fouling.^600^

The challenges in developing continuous implanted glucose sensors are fairly common issues for implanted sensors in general, particularly where constant and prolonged sensing is needed. For example, neural electrodes used to develop brain–computer interfaces for the physically disabled also struggle with the brain foreign body response, which leads to glial scarring and loss of signal. Along these lines, Rao et al. demonstrated that PEG-containing PU coatings could improve the biocompatibility of neural electrodes.^601^ An emerging trend has led to the rise of hydrogel coatings that can play an *active* role as a component of the device themselves, especially as electrically conductive hydrogels.^532,558,602^ Properties of conductivity, self-healing, and stimuli-responsiveness are exploited in poly(NIPAM-*co*-β-CD) hydrogels that are promising for applications such as artificial organs and as pressure-dependent sensors.^603^ PAm-LiCl hydrogels have been integrated with cephalopod-(bio)inspired materials that serve as electrodes for dynamic optical and tactile sensing^604^ as ionic conductors capable of operation at high frequencies^605^ and as actuators.^606^ Overall, the use of hydrogel coatings may represent an important step closer toward maintaining long-term and reliable connections between sensors and the tissues they are probing.

#### 5.2.5 Future Directions for Hydrogel Coatings

Implants face multifaceted and complex interactions with the body and are often expected to simultaneously manage numerous processes including infections, the foreign body response, and whatever the device’s specific function happens to be. Hydrogel coatings can be useful for implants seeking to achieve this level of multifunctionality, but it is likely that coatings will need to take on multiple roles as well. Incorporating small molecules and antithrombogenic drugs into an antifouling hydrogel, for example, may provide added benefits for implanted stents. Achieving fully integrated multifunctional hydrogel coatings will require novel strategies and materials, as well as deeper understanding of what occurs at the host–device interface. For example, physical modifications of hydrogel topography (e.g., bioinspired patterning such as biomimicking the micropatterning of a lotus leaf or snail shell) of materials may provide new ways to prevent fouling.^607^ Substrate roughness also appears to affect adhesion of matter onto the surface of materials and can be tuned during synthesis.^574^ Although we now have many ways to incorporate hydrogels onto devices, there remains uncertainty on the mechanical properties that hydrogel coatings require in order to simultaneously withstand the forces from everyday movement, prevent degradation, and integrate well with surrounding tissues. The design of materials and devices will require optimization on all fronts, as a number of these factors may influence the antifouling properties of these systems.

Finally, advances in materials and exploiting properties depends ultimately on a deep understanding of the mechanisms underlying their use and failure. For example, most antifouling/microbial efforts are aimed at preventing the initial attachment of proteins and matter on the surface, yet we only have a limited understanding of the mechanism of adhesion, which may include factors beyond energetic and kinetic considerations. Computational studies to elucidate more mechanisms at play at the host–device interface could provide valuable new insights. In biological fouling, albumin, lysozyme, and fibrinogen are often pinpointed as major contributors to fouling, though not all proteins or species that contribute to fouling may be identified in such complex fluids. Surface-sensitive techniques such as AFM, XPS, FTIR, SPR, and QCM are often used to study material surfaces and protein adhesion, but materials need to be assessed in environments that more closely mimic that of their application. In analysis of surfaces, time-of-flight secondary ion mass spectrometry (ToF-SIMS) allows high-resolution images and analyses of surfaces. Cryo-TOF-SIMS/SEM systems have been used in analysis of wood tissue *in planta*; implementing this for biological samples could provide important compositional information about changes in surface chemistry after implantation.^608^ A push toward more complex testing assays in *in vivo* or physiological conditions is critical for optimizing and defining design criteria for hydrogel coatings for biomedical devices. Coupling a greater understanding of these mechanisms with the growing capabilities of modern materials science may well unlock even greater benefits for hydrogel coatings in the years to come.

## 6 Clinical Translation of Biomedical Hydrogels

Consistent with their numerous biomedical capabilities, hydrogels have been used clinically for some time. This is not to say, however, that the clinical translation of hydrogels is trivial or straightforward. While significant infrastructure has been developed to reliably fabricate more traditional hydrogels (e.g., covalent gels) for applications ranging from contact lenses to bandages, the emergent generation of hydrogels can be considerably more complex from both a physical and chemical point of view. These complexities can introduce challenges when trying to meet federally required current Good Manufacturing Practices (cGMP) and Quality System Regulations (QSRs). These challenges can be further exacerbated for emergent hydrogel formulations that rely on nanoparticles or other forms of nanotechnology, since manufacturing standards for biomedical nanotechnologies are still not especially well established. Nevertheless, exciting progress in the clinical translation of novel formulations, and particularly injectable formulations, is evident from currently ongoing clinical trials and will be the focus of this section. Here, we will evaluate the major application areas where clinical work is ongoing and compare these clinical technologies to the emergent technologies being implemented in the preclinical studies discussed in our prior sections. We round out the discussion of clinical translation with manufacturing considerations that may often be overlooked during the early stages of preclinical hydrogel development, which we hope will be useful for researchers with an eye toward eventual translation.

### 6.1 Injectable Hydrogels Currently in the Clinic

Hydrogels have been part of the clinical landscape for some time, and their current usage in the clinic was recently reviewed and analyzed by Mitragotri and co-workers.^78^ Their meta-analysis showed that the plurality of hydrogels in clinical trials is for ocular applications, such as soft contact lenses. However, outside of ocular therapies, the remaining hydrogels spanned a diverse set of applications including pain management, tissue regeneration, wound healing, cosmetic procedures, cancer therapy, and urinary disorder treatments. By and large, ocular hydrogels and wound healing dressings are composed of noninjectable hydrogels. Here, we discuss how the clinical applications and therapeutic strategies of injectable hydrogels differ and relate to the preclinical research discussed in prior sections.

Injectable hydrogels are a quickly progressing area of biomedical research, having already led to numerous approvals in the US and in Europe.^78^ Overall, a significant portion of current clinical trials seek to determine what additional benefits and disease indications are possible for already-approved formulations. Nevertheless, several trials of novel formulations are paving the way to the clinic for more experimental and multifunctional injectable hydrogels. Table 1 contains the active, recruiting, and not yet recruiting clinical trials using injectable hydrogels, as of early 2020.

**Table 1:** Injectable Hydrogels in Clinical Trials as of January 2020

Indication	Study Title	Phase	Material	ID
Bladder Carcinoma	TracelT Hydrogel in Localizing Bladder Tumors in Patients Undergoing Radiation Therapy for Bladder Cancer	N/A	PEG hydrogel	NCT03125226
Oropharyngeal Cancer	TraceIT Tissue Marker to Mark the Primary Resection Bed Margins of Oropharyngeal Cancers	1	PEG hydrogel	NCT03713021
Rectal Tumors	Organ-sparing With TraceIT for Rectal Cancer Radiotherapy	N/A	PEG hydrogel	NCT03258541
Rectal Cancer				
Advanced Cancer				
Pancreatic Adenocarcinoma	Radiopaque Hydrogel in Patients Undergoing Radiotherapy for Pancreatic Cancer	N/A	PEG hydrogel	NCT03307564
Prostate Cancer	Single Fractions SBRT for Prostate Cancer	N/A	PEG hydrogel	NCT04004312
Pancreatic Adenocarcinoma	Radiopaque Hydrogel Spacer in Patients Undergoing Radiotherapy for Pancreatic Cancer	N/A	PEG hydrogel	NCT03998566
Mucositis Oral	MucoLox Formulation to Mitigate Mucositis Symptoms in Head/Neck Cancer	2	Undisclosed mucoadhesive polymer	NCT03461354
Head and Neck Cancer				
Colorectal Cancer	Local Immunomodulation Combined With Radiofrequency Ablation for Unresectable Colorectal Liver Metastases (LICoRN-01)	1/2	Undisclosed mucoadhesive hydrogel	NCT04062721
Osteoarthritis, Knee pain	New Hydroxyethyl Cellulose Hydrogel for the Treatment of the Pain of Knee Arthrosis (PROMGEL-OA)	N/A	Hydroxyethyl cellulose hydrogel	NCT04061733
Osteoarthritis	Intra-articular Polyacrylamide Hydrogel in Knee Osteoarthritis	N/A	Polyacrylamide hydrogel with silver ions	NCT03897686
Osteoarthritis, Knee	Treatment of Knee Osteoarthritis With PAAG-OA (ROSA)	N/A	Polyacrylamide hydrogel	NCT04045431
Osteoarthritis, Knee	PAAG-OA Treatment for Knee Osteoarthritis	N/A	Polyacrylamide hydrogel	NCT04179552
Osteoarthritis, Knee	Aquamid Reconstruction for Osteoarthritis of the Knee	N/A	Polyacrylamide hydrogel	NCT03067090
Heart Failure	A Pivotal Trial to Establish the Efficacy and Safety of Algisyl in Patients With Moderate to Severe Heart Failure (AUGMENT-HFII)	N/A	Alginate	NCT03082508
Dilated Cardiomyopathy				
Heart Failure With Reduced Ejection Fraction				
Sensorineural Hearing Loss	FX-322 in Adults With Stable Sensorineural Hearing Loss	2	Undisclosed	NCT04120116
Noise Induced Hearing Loss				
Sudden Sensorineural Hearing Loss				
Chronic Kidney Disease	A Study of a Renal Autologous Cell Therapy (REACT) in Patients With Chronic Kidney Disease (CKD) From Congenital Anomalies of the Kidney and Urinary Tract (CAKUT).	1	Gelatin thermogel	NCT04115345
Congenital Anomalies of Kidney and Urinary Tract				
Uterine Fibroid	Safety and Efficacy of ActamaxAdhesion Barrier in Women Undergoing Laparoscopic Abdominopelvic Surgery/Myomectomy	N/A	Undisclosed	NCT03450421
Lung Biopsy	Effect of Autologous Blood Patch Injection Versus BioSentry Hydrogel Tract Plug in the Reduction of Pneumothorax Risk Following Lung Biopsy Procedures	3	PEG hydrogel	NCT02224924
Latent Autoimmune Diabetes in Adults	Injections of Glutamic Acid Decarboxylase (GAD) for LADA Type of Diabetes	2	Aluminum hydroxide	NCT04262479
Urinary Incontinence	BOTOX Intravesical Instillation in Participants With Overactive Bladder and Urinary Incontinence (APOLLO)	2	Undisclosed	NCT03320850
Overactive Bladder With Urinary Incontinence				

Of the 20 trials identified, 25% were for bone, joint, or cartilage repair applications. These treatments implement hydrogels as tissue scaffolds,^609^ using either polyacrylamide or hydroxyethyl cellulose hydrogels to treat conditions that include arthrosis and osteoarthritis. Notably, these interventions generally do not aim to deliver drugs or therapeutic cells with hydrogels, with the exception of Argiform, a poly(acrylic acid) (PAA) gel formulated with antibacterial silver ions. But in general, the injectable hydrogels being tested in the clinic for bone/cartilage regeneration are resorbable and provide structural support or scaffolding for endogenous cells as they degrade. PAA gels in particular appear to foster invasion by endogenous cells, which ultimately leads to integration with and resorption by the body.

Within the bone/join/cartilage repair application area, three naturally derived hydrogel formulations have already been approved in the US and Europe. In contrast to the materials currently undergoing trials, these hydrogels engage through specific receptor–ligand interactions and are used to deliver drugs in some cases. One of these is the hyaluronic acid-based EUFLEXXA, which acts as a mechanical scaffold but also has natural ligand–receptor interactions through CD44 and other proteins to more actively engage endogenous cells. The other two formulations are collagen-based and are used to deliver growth factors (BMP-2 in the INFUSE system and OP-1 in the OP-1 Putty system). Whether passive scaffolding or more active/drug eluting strategies prove to be more effective has yet to be determined.

The plurality of injectable hydrogel clinical trials identified (40%) pertained to cancer, evaluating indications that included pancreatic, bladder, rectal, prostate, and head and neck cancers. While this is a large percentage, the majority of these trials are seeking to find additional utility for the already approved SpaceOAR and TraceIt systems, both PEG-based hydrogels developed by Boston Scientific for imaging and radiotherapy applications. Nevertheless, the therapeutic approaches across these clinical trials varied widely, and included improving tumor imaging, reducing side effects from radio or chemotherapy, and a local adjuvant immunotherapy.

The iodinated TraceIt hydrogel system has been successful for improving tumor imaging following resection. The hydrogel persists in tissues for up to three months, where it provides a high contrast in image-guided radiation therapy, which can allow physicians to more selectively treat tissues where residual tumors are most likely to reside. This product has received clearance for clinical use as a radiographical marker for soft tissues, and current clinical trials seek to determine additional indications and applications in cancer imaging and radiotherapy. The SpaceOAR system was developed and approved as a tool for shielding vulnerable tissue from damage during radiotherapy of prostate cancer. In addition to these gels being radio-opaque, they serve as physical spacers between cancer tissues and other delicate organs. Both systems continue to be explored in clinical trials as a means to physically obstruct radiation in order to shield sensitive healthy tissues during cancer radiotherapy. This particular approach may be useful for prostate, rectal, and pancreatic cancers, where the tumors are adjacent or near to delicate organs. Notably, these products appear to be providing significant clinical value simply by persisting in the locations where they are administered. Modification of their formulations to make them radio-opaque or to include a tracer for imaging also allows for much safer and effective radiotherapy. Notably, these trials are also demonstrating that precise hydrogel injection to areas near deep-tissue tumors is possible using ultrasound or other image-guided techniques. These observations ease concerns that injectable hydrogel therapies would be difficult to adapt to treating nonsuperficial tumors.

Despite the significant amount of preclinical research into drug delivery systems, there are few trials evaluating such systems in the clinic. This is perhaps due to the fact that prior to immunotherapy, local drug delivery was of limited usefulness to the types of cancer patients who enroll in clinical trials in the first place—those with unresectable and metastatic cancer. The recent groundswell in local cancer immunotherapy work may presage a wave of immunomodulatory hydrogels entering into clinical trials in the near future. However, this may be complicated by scalable manufacturing and GMP requirements, which are always a challenge for new therapeutic approaches.

The success or failure of early clinical trials with immunomodulatory hydrogels will be of intense interest to the field, such as the upcoming LICoRN-01 trial that will evaluate the combination of chemotherapy, radiotherapy, and local immunomodulatory hydrogels in patients with unresectable colorectal cancer. Following a round of chemotherapy and radiofrequency ablation therapy, two metastatic lesions will be intratumorally injected with a muco-adhesive hydrogel containing GMCSF and a TLR agonist. The preclinical data supporting this approach indicated a tolerable and effective therapy in a murine model of colorectal cancer.^610^ The results of this trial will provide valuable insight into the translatability of local immunomodulatory hydrogels and the technical challenges of intratumoral injection and of producing pharmaceutical grade immuno-modulatory hydrogels at scale. Of special interest will be the extent of the abscopal effect or the antitumor effect on distant, untreated lesions. Immunomodulatory hydrogels will need to mount abscopal effects for continued cancer clinical trials to be feasible. However, it cannot be discounted that there may be significant interest in hydrogel-based vaccines in the wake of the SARS-CoV-2 pandemic, which may ultimately provide some of the earliest and most comprehensive data on immunomodulatory biomaterials in humans.

The remaining trials span a fairly diverse clinical landscape and echo many of the topics discussed in the preclinical sections. Two studies are evaluating hydrogels for surgical applications. One study is evaluating the sprayable Actamax system as a way to prevent adhesions following certain laproscopic surgeries, and the other study uses an injectable hydrogel plug (BioSentry) to close the wound left in lungs after a biopsy. Tissue regeneration trials are especially interesting, such as the Algisyl trial that seeks to determine the safety and efficacy of an injectable alginate hydrogel as a scaffold for left ventrical regeneration following heart failure. Algisyl is already approved in Europe, and this trial may provide entry into the US market. Delivery of therapeutic cells is being evaluated in the REACT trial which aims to deliver renal cells to patients in a gelatin hydrogel to treat chronic kidney disease. And regenerative drug delivery is being evaluated in the FX-322 trial, which uses a poloxomer-based hydrogel to deliver a proprietary blend of small molecules to stimulate regrowth of hearing cells. Overall, current clinical trials are exploring diverse and wide-ranging capabilities of injectable hydrogels.

### 6.2 Manufacturing and Scale Up Considerations for Translation

Scaling and manufacturing hydrogels for commercial products remains a challenging process. Despite the critical nature of scalable manufacturing in bringing these technologies into the clinic, there is surprisingly little attention provided to this topic in the literature. This may be due to a general lack of interest in these topics, difficulty for academic groups to explore scaled-up manufacturing, insufficient communication between academic and industry partners, or the lack of research funding. More likely than not, all of these contribute to the dearth of studies to improve the process engineering of these biomaterials. Here, we briefly summarize several regulatory and manufacturing considerations that should be taken into account when developing hydrogels with translation in mind.

Typically, hydrogels are classified by the United States FDA as a device, biologic, or drug depending on the application. The quickest and most inexpensive regulatory pathway would be as a device with a 510(k) designation.^611^ Devices have quicker approval processes (around 5 years), but if the hydrogel is delivering a drug or cells, it is most often classified as a combination product requiring 7–10 years for approval and $50–300 million for development and testing.^189^ Most hydrogels are currently fabricated in small batches for preclinical studies, but large-scale reactions and processes must be designed and optimized through officially recognized Good Manufacturing Processes (GMP) before approval and commericalization.^78^ For widespread utilization, hydrogels should be able to be safely fabricated on the kiloton scale.^612^ The considerable challenge of this scale up should not be underestimated, even from moving from small to large animal preclinical work. From our own experience, the volume of sprayable hydrogel used in preclinical studies of adhesion barriers ranged from 0.25 mL per subject for rat studies to 50–75 mL for ovine studies.

The chemical components making up the hydrogel may also affect scaling and manufacturing processes. If hydrogels contain chemical moieties that degrade due to hydrolysis or other processes over time, proper storage and formulation processes must be anticipated and accounted for. For example, can a new hydrogel formulation be cryopreserved and lyophilized without damaging the product or encapsulated drugs? Does the formulation remain stable at room temperature under mild agitation, or does it require refrigeration? If refrigeration is required, is 4 °C sufficient or are freezing temperatures (and if so does it require −20 or −80 °C) needed? These are all essential questions for commercial feasibility, yet they are rarely explored in either *in vitro* or preclinical studies.

Individual components of hydrogels might present unique regulatory challenges, especially as nanotechnology and biologicals are incorporated into next-generation formulations. So while the advantages of nanoparticles in hydrogel formulations are readily apparent in the preclinical literature, there is the issue that nanomedicines have generally been difficult to translate to the clinic.^613^ Similarly, many hydrogels used for preclinical studies rely upon natural biopolymers, such as alginate, cellulose, or collagen, but often these biopolymers exhibit batch-to-batch variation that may complicate the ability to satisfy robust quality control metrics.^53^

Beyond the difficulty in producing their individual components, hydrogels that require defined macroscale architecture (e.g., macroporosity) can be difficult to produce at larger scales. Recent work on this issue has led to some progress, with Mikhalovsky and co-workers reporting cryogelation methods that increase the scale from a few milliliters up to 400 mL.^614^

One key challenge of scaling hydrogel products is maintaining sterility, which is required to receive approval for commercial products. Due to the high-water content in hydrogels, it is challenging or impossible to sterilize hydrogel products by traditional methods, such as autoclaving, without damaging the product.^189^ Often the only viable method is to sterilize components and processes themselves before hydration. This of course requires all subsequent steps be performed under aseptic conditions, which presents a considerable process challenge. Some techniques for sterilization include filtration, radiation (gamma-rays and e-beams), and heating procedures.^615^ Of course, care should be taken to make sure that at least one of these techniques is compatible/nondestructive for the various components of a novel hydrogel therapy.

There are a variety of additional pitfalls and challenges for translation that could be evaluated from early design stages. For example, depending on the nature of gelation for hydrogel synthesis, large quantities of heat or other byproducts may result and must be safely handled. Along these lines, hydrogels that form through self-assembly and simple mixing procedures may have an advantage during scaled up manufacturing.^612^ And as discussed in the prior section on injectable hydrogel rheology, careful assessment of hydrogel rheology can identify what applications a formulation can feasible accomplish when translated to clinically relevant geometries (e.g., forces required to inject through a syringe or catheter of different gauge/lengths).

Most biomedical materials literature is goal-oriented toward clinical translation, and the field has amassed reports that painstakingly characterize the therapeutic efficacy and mechanisms of novel biomaterials, such as hydrogels. Of course, biomedical translation depends upon this efficacy, but translation also depends on the material’s ability to be manufactured at scale and to meet regulatory standards. Nevertheless, this type of assessment is rare in the literature. This is not to say that all biomaterials research should be limited to materials that would be readily scaled and manufactured based on today’s infrastructure. After all, studies with highly tunable but difficult-to-translate materials can be very helpful and may elucidate generalizable, materials-agnostic design criteria for specific biomedical applications. Rather, it is to say that increased transparency in current fabrication capabilities of biomaterials could identify current bottlenecks, thereby elevating their importance and unlocking research and funding to resolve them.

## 7 Opportunities for Hydrogels beyond Biomedical Applications

While previous discussions primarily focused on hydrogels for biomedical applications, many of the principles discussed throughout this review are analogous and transferrable to diverse nonbiomedical applications including agriculture,^616,617^ water remediation,^618^ oil recovery,^619,620^ water storage,^621^ biofuel production,^622,623^ and cosmetics.^624,625^ In this section, we briefly summarize several exciting areas for hydrogel technology outside of medicine and point out areas where desired functionality overlaps with the capabilities being developed for biomedical hydrogels. It is our hope that this discussion may inspire materials researchers to consider opportunities across a variety of societally impactful but underexplored application areas.

The unique materials properties of hydrogels are often acquired through relatively low (<10%) amounts of solids, enabling simple processing and low costs that are required for commercial applications, which often can necessitate hundreds of millions of gallons of product a year.^626,627^ Additional functional complexities such as active ingredient encapsulation, triggered and controlled cargo release, and degradation rate can all be engineered through appropriate chemical and mechanical design of the hydrogels.^628−630^ Altogether, these attributes combine to make hydrogel technology not only broadly useful but also translationally feasible for diverse commercial and industrial applications.

Biofuel production and biofabrication are a particularly interesting area where there is considerable overlap with hydrogels used to manipulate cells. However, instead of mammalian cells, the ability to manipulate microbes opens the door to completely novel capabilities. For example, Johnston et al. engineered methacrylate-based hydrogels to immobilize microbes that would not otherwise be compatible in liquid suspension.^622^ Careful design of the hydrogel chemistry and cross-linking enabled these materials to be processed through extruders, to immobilize and stabilize microbes, and to also allow for repeated lyophilization–rehydration cycles without cryoprotectants. These properties allowed for on-demand microbial production of small molecules and active peptides, and the hydrogels demonstrated up to a year of continuous fermentation of yeast to produce ethanol.

In the field of cosmetics, hydrogel mechanical properties are essential for providing long-lasting benefits. Along these lines, Yu et al. engineered an elastic cross-linked polymer layer that mimicked the mechanics of youthful skin.^624^ In this example, the hydrogel structure allowed the topically applied material to be breathable without irritation, while having the elastic properties of youthful skin. Furthermore, the authors conclude that in addition to the isotropic stresses applied by the hydrogel, the hydration properties of the hydrogel also contributed to improvements in skin mechanics and appearance.

One particularly promising area for hydrogels is environmental engineering, where the biocompatible and hydrophilic properties of this technology allow for some remarkable capabilities. Importantly, this is an area that may have considerable implications for the world as it adapts to the consequences of a warming climate. In particular, hydrogel technology provides new options for water remediation. A critical design criteria for water remediation is the adequate mass transport of water through the hydrogel, which is similar to the considerations for nutrient transport in a variety of hydrogels for cellular therapies. In one recent study, Kumarasamy et al. created a polymer resin using fluorophilic and charged functional groups to rapidly and selectively remove polyfluorinated alkyl substances from water.^631^ In this example, upon exposure to water, the resins form hydrogels where the network structure allows for rapid mass exchange throughout the material, while exposing the water to the fluorophilic and charged functional groups for rapid sorption.

Agriculture accounts for 69% of annual water usage worldwide with 40% of the global population projected to be living in areas of severe water stress by 2050.^600,632^ Hydrogels have played an important role in facing these challenges by increasing soil water holding capacity and minimizing water runoff. Specifically, these hydrogels are formed through swelling of superabsorbent polymers (SAPs), which can result in fluid absorption up to 1000 times their dry weight.^626,627^ These SAPs are often delivered as powders or granules and can be formed through physical or chemical cross-linking of synthetic or natural polymers. Appropriate choice of materials, structure, and chemistry depends on judicious balancing of the target functionalities: water absorption capacity, rate of absorption, swelling size, durability (operation and storage), toxicity, biodegradability, and cost.

Synthetic SAPs use monomers such as acrylic acid, methacrylic acid, siloxanes, and various acrylamides to form chemically cross-linked, swellable materials. These materials benefit from having a vast chemical space for tunability, typically large swelling capabilities, and mechanical robustness (stiffness and elasticity to retain structure under soil compression) at low concentrations.^626^ For example, Woodhouse and Johnson demonstrated that dry polyacrylamide, poly(vinyl alcohol), and starch copolymer mixed into sand all enhanced water efficiency (g of dry plant matter produced per kg of water) and increased the number of days until plants wilted to 16, up from 3 days.^633^ These water enhancing properties have also found utility in wildfire retardant strategies as “short-term” retardants.^634−637^ In these strategies the increased retention of water allows for treatment of buildings and fuel in the path of encroaching fires, but the overall efficacy is limited since the water rapidly evaporates (<1 h) in wildfire conditions.^636,638,639^

Beyond water enhancement, synthetic SAPs have also demonstrated utility in erosion prevention and ecological soil restoration.^640^ These polymers are designed to functionally mimic humus and engineered to be hydrophilic and capable of binding specific soil cations. For example, researchers use a polyacrylate polymer to remediate soils contaminated by copper from fungicides, reducing the amount of copper to 0.11 times the control with 0.1% of polymer blended in the soil.^641^ The ability to tune the density of hydrophilic and ion-binding moieties provides a flexible strategy toward tailoring synthetic SAPs for specific soil conditions and remediation approaches. However, despite these many advances, many SAP-based hydrogels (e.g., polyacrylates) are often limited in biodegradability and renewable production, raising concerns about environmental and human toxicity.^642−645^ Like in many biomedical applications, a vast proportion of agricultural uses of SAP-based hydrogels require biodegradability (e.g., microbial degradation and hydrolysis), biocompatibility (e.g., nontoxic, no accumulation, minimal changes in soil chemistry), and renewability (e.g., sustainable synthesis), leading researchers to explore natural polymers as alternatives.

Similar to hydrogels in biomedical applications, researchers can cross-link the hydrogel network through many of the traditional chemical and physical cross-linking methods. One example is carboxymethylcellulose (CMC), which has been popular for engineering naturally derived SAPs due to its high-water-absorbency ability and swelling rate thanks to its abundant hydroxyl and carboxylic acid groups.^646,647^ In this example, CMC was mixed with clay particles to form hydrogels that increased the time to release 50% of the encapsulated herbicide from <1 h for the commercial standard to ∼2 to 500 h, illustrating the analogous engineering strategies and criteria to biomedical applications.^647^ These similarities have inspired researchers to expand the use of hydrogels beyond soil remediation and water enhancement to more ambitious and complex cargo (e.g., pesticides, herbicides, fungicides, fertilizers, retardants) delivery.

Analogous to delivery of biotherapeutics, controlled delivery of cargo for agriculture and sustainability provides several benefits over site retention, release kinetics, and triggered release strategies. As in drug delivery, cargo release can be categorized as passive release or active release. In passive release, researchers often leverage chemical potential gradients or natural degradation to drive both water and cargo delivery to the surrounding soil. For example, Cheng et al. use an acrylic acid-based hydrogel chemically cross-linked by urea and *N,N’*-methylenebis(acrylamide) that passively released nitrogen (in the form of urea) to the surrounding soil over ∼40 days.^648^ In this system, the poly(acrylic acid) backbone facilitated enhanced water-swelling, while the hydrolysis rate of urea dictated the extended release time frame of N delivery to the soil.

**Figure 34:**
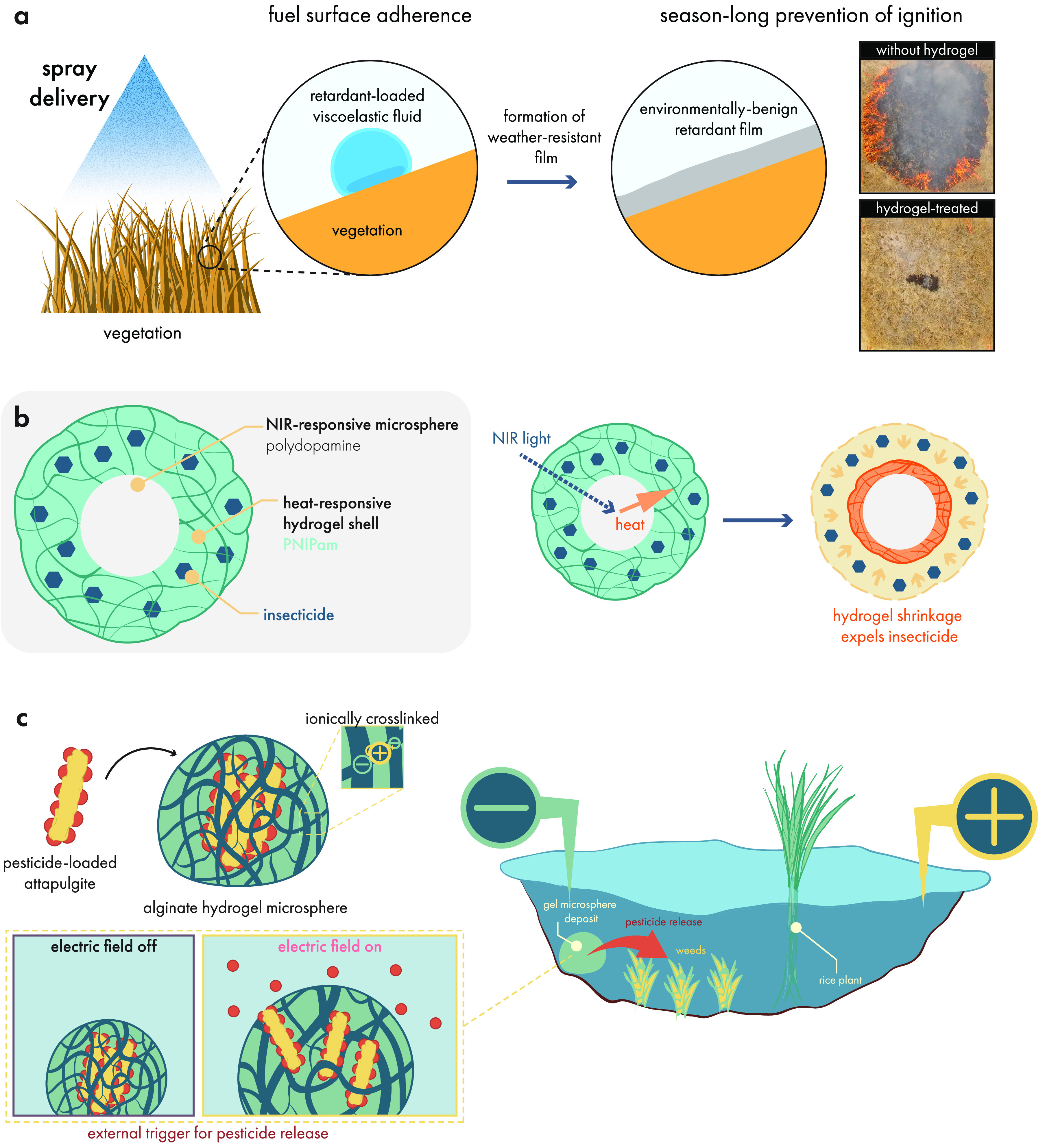
Many functions that have been developed toward biomedical applications can be used to solve analogous problems outside of the clinic, particularly in environmental engineering and agriculture. (a) Retardant loaded viscoelastic fluids are able to be deployed by traditional spraying methods onto vegetation. The engineered rheological properties allow the retardant to have enhanced adherence, surface wetting, and retention on wildland vegetation. As the materials dry, a weather-resistant retardant film is formed on the vegetation, providing season-long prevention against wildfire ignitions. Adapted with permission from Yu et al.^21^ Copyright 2019. (b) Microspheres combining a NIR-responsive polydopamine core with a heat-responsive PNIPAm hydrogel shell, which holds the insecticide. The polydopamine core is able to absorb photons in the NIR region and produce thermal energy that triggers shrinkage of the PNIPAm shell. Once the hydrogel shell shrinks, the insecticide cargo is released. Original illustration inspired by the work of Xu et al.^649^ (c) Attapulgite and calcium alginate carrier system that enables electrical-triggered release of pesticides. Attapulgite facilitates adsorption of the pesticide, while the calcium alginate mixture creates the cross-linked hydrogel. Once an electric field is applied, the migration of charged species leads to release of the charged pesticide (glyphosate) to the surrounding environment. Original illustration inspired by the work of Zhang et al.^650^

Our group recently reported another passive delivery strategy for wildfire prevention, where the hydrogel’s mechanics enhanced adherence of fire retardants on target wildland fuels (Figure 34a).^21^ In this example, hydroxyethyl cellulose and methylcellulose were cross-linked with colloidal silica particles to form viscoelastic fluids that could be sprayed and adhered onto vegetation.^21^ In these studies, the enhanced mechanical (e.g., relative elasticity, extensional viscosity, and dynamic yield stress) and physicochemical properties (e.g., surface tension and spreading coefficient) provided by the cellulose–silica particle network enhanced adherence of fire retardants on wildland fuels from 44% to 70% after spraying and was able to completely prevent ignition of dry grass even after half an inch of rain.^21^ Notably, the ability for a dynamic hydrogel to be sprayed and coat complex shapes was an essential capability for this approach and suggests that sprayable hydrogels may be especially important environmental interventions.

Active release strategies open the doors to a vast multitude of available stimuli and chemical methods to trigger release. This broad landscape of design strategies has led researchers to develop many creative ways to create hydrogels for triggered release of nutrients, pesticides, and other agrochemicals.^628−630^ The primary draw of these triggered release strategies is to enhance delivery efficiency (e.g., timing, location, and dosing) and reduce pollution from leakage, surface migration, or off-target delivery. Along these lines, Xu et al. combine the near-infrared (NIR)-responsive polydopamine (PDA) with the temperature-responsive PNIPAm to form microspheres for a temperature-responsive release of pesticides to improve site accuracy and efficiency of delivery (Figure 34b).^649^ While the PDA core converted photons to thermal energy, the PNIPAm shell encapsulated the pesticide and shrunk when exposed to elevated temperatures, which released the pesticide. Using pH as their stimulus, Xiang et al. demonstrated controlled pesticide release using an attapulgite, PDA, and calcium alginate hydrogel.^651^ In this system, the attapulgite was modified with PDA, which coordinated with the pesticide. This mixture was then mixed into an alginate solution, which was subsequently cross-linked with calcium to form hydrogel spheres. The pH-responsiveness originated from dissolution of the calcium alginate network due to ion exchange of the cross-linking calcium with sodium when increasing pH from 5.5 to 8. Similarly, many poly(acrylic acid) -based hydrogel systems are capable of incorporating pH triggers for cargo release due to the pH sensitivity of the hydrogen bonds that hold the network together.^652^ Beyond temperature and pH, researchers have also explored electrical stimuli for triggered release, citing high energies for temperature triggers and harmful soil chemistry effects of pH triggers.^650,653,654^ In one example, Zhang et al. demonstrated in water tank and pot (rice plants and weeds) experiments that they could use attapulgite and calcium alginate to form an electrically triggered hydrogel sphere for releasing glyphosate, a commonly used pesticide (Figure 34c).^650^ The electric field induced Coulombic forces on the anionic calcium alginate network, enlarging pores and allowing the negatively charged glyphosate to release from the hydrogel.

Overall, this section provides a brief introduction into the nonbiomedical applications of hydrogels, which includes opportunities in a wide range of sustainability related applications and cosmetics. Many of the materials design strategies for these applications are analogous to hydrogels used in the biomedical field and suggest that concepts developed for biomedical applications may be transferrable to diverse challenges and vice versa. This being said, the constraints in cost, environmental compatibility, and delivery strategies ultimately offer a very different challenge for fields such as environmental engineering. In particular, the multitude of available materials design strategies frequently leads to tenuous rationalization of starting materials, chemistry, and complexity in exchange for demonstration of feasibility. For this reason, commercial hydrogel products in agriculture and sustainability are still limited in scope, with hydrogels capable of multifaceted functionality out of reach due to the high costs of synthesis and unscalable production. This reality not only offers ample opportunity for creative innovation in hydrogel fabrication but also demands for interdisciplinary collaborative teams between materials engineers, environmental scientists, and industry partners for hydrogel technologies to be realistically applied across a spectrum of commercial applications.

## 8 Conclusions

Although the applications discussed throughout this review are very diverse, there are recurrent themes that tie together these efforts. The concept of controlled trafficking of molecules though hydrogels, for example, is highly relevant for drug, cell, and pesticide delivery. Within this theme of controlled release, there are some inconsistencies in drug delivery strategies with hydrogels that are worth assessing. One of the main strengths of these approaches is to localize treatment to the vicinity of the hydrogel, yet many studies have evaluated the efficacy of gels distant from the target tissue. In these systems, the hydrogel is acting like a long-term infusion of drugs, which could be beneficial if the drug is largely nontoxic (e.g., passive immunization applications). But for toxic drugs such as chemotherapy, the question of tolerability is considerable, and these studies ought to evaluate toxic side effects. While peritumoral or intratumoral injection of chemotherapeutic hydrogels is not feasible for metastatic disease, it is an appropriate approach for adjuvant therapy or in the context of cancer immunotherapy, which can drive systemic responses from local immuno-modulation.

Perhaps the most ubiquitous theme across the studies discussed here is the interdisciplinary skillset required to unlock the potential of hydrogels in each focus area. Whether being developed for vaccines, surgeries, or wildfire prevention, each application places highly specific demands on hydrogels. Identifying these demands is often not trivial and requires effective communication between materials scientists and their collaborators in these subject areas. Usually, adapting to the requirements of a given application necessitates innovation on the materials end, bringing together diverse specialties such as chemistry, bioengineering, and mechanical engineering. Testing and proving the value of these materials then requires materials groups to become literate in the conventions and techniques of one or more unfamiliar disciplines, such as cancer biology, immunology, surgery, or microbiology. Especially as modern medicine continues to become increasingly reliant on highly advanced proteomic, transcriptomic, and genomic techniques, engineering hydrogel therapies to mediate complex biological interventions will require high levels of expertise in systems biology, genetics, and biochemistry. Thus, as biomaterials become more sophisticated, we anticipate that highly effective and interdisciplinary research teams will be essential to both develop and translate these technologies to solve society’s most urgent biomedical problems.
